# In vivo epigenetic editing of *Sema6a* promoter reverses transcallosal dysconnectivity caused by *C11orf46/Arl14ep* risk gene

**DOI:** 10.1038/s41467-019-12013-y

**Published:** 2019-09-11

**Authors:** Cyril J. Peter, Atsushi Saito, Yuto Hasegawa, Yuya Tanaka, Mohika Nagpal, Gabriel Perez, Emily Alway, Sergio Espeso-Gil, Tariq Fayyad, Chana Ratner, Aslihan Dincer, Achla Gupta, Lakshmi Devi, John G. Pappas, François M. Lalonde, John A. Butman, Joan C. Han, Schahram Akbarian, Atsushi Kamiya

**Affiliations:** 1 Friedman Brain Institute and Department of Psychiatry, Icahn School of Medicine at Mount Sinai, New York, NY 10029 USA; 2 Department of Psychiatry and Behavioral Sciences, Johns Hopkins University School of Medicine, Baltimore, MD 21287 USA; 3 Department of Pharmacology and System Therapeutics, Icahn School of Medicine at Mount Sinai, New York, NY 10029 USA; 4 Department of Pediatrics, New York University School of Medicine, New York, NY USA; 5 Human Genetics Branch, National Institute of Mental Health, Bethesda, MD 20892 USA; 6 Diagnostic Radiology Department, The Clinical Center of the National Institutes of Health, Bethesda, MD 20892 USA; 7 Unit on Metabolism and Neuroendocrinology, Eunice Kennedy Shriver National Institute of Child Health and Human Development, National Institute of Health, Bethesda, MD 20892 USA; 8 Departments of Pediatrics and Physiology, University of Tennessee Health Science Center, and Children’s Foundation Research Institute, Le Bonheur Children’s Hospital, Memphis, TN 38103 USA

**Keywords:** Biochemistry, Biogeochemistry, Histocytochemistry, Mutagenesis, RNAi

## Abstract

Many neuropsychiatric risk genes contribute to epigenetic regulation but little is known about specific chromatin-associated mechanisms governing the formation of neuronal connectivity. Here we show that transcallosal connectivity is critically dependent on *C11orf46*, a nuclear protein encoded in the chromosome 11p13 WAGR risk locus. *C11orf46* haploinsufficiency was associated with hypoplasia of the corpus callosum. *C11orf46* knockdown disrupted transcallosal projections and was rescued by wild type C11orf46 but not the C11orf46^R236H^ mutant associated with intellectual disability. Multiple genes encoding key regulators of axonal development, including *Sema6a*, were hyperexpressed in *C11orf46*-knockdown neurons. RNA-guided epigenetic editing of *Sema6a* gene promoters via a dCas9-SunTag system with C11orf46 binding normalized SEMA6A expression and rescued transcallosal dysconnectivity via repressive chromatin remodeling by the SETDB1 repressor complex. Our study demonstrates that interhemispheric communication is sensitive to locus-specific remodeling of neuronal chromatin, revealing the therapeutic potential for shaping the brain’s connectome via gene-targeted designer activators and repressor proteins.

## Abstract (web summary)

Although many neuropsychiatric risk genes are known to contribute to epigenetic regulation of gene expression, very little is known about specific chromatin-associated mechanisms that govern the formation and maintenance of neuronal connectivity. Here, the authors report that transcallosal connectivity is critically dependent on C11orf46/ARL14EP, a nuclear protein encoded in the chromosome 11p13 WAGR risk locus, and that RNA-guided epigenetic editing of hyperexpressed Sema6a gene promoters in C11orf46-knockdown neurons resulted in normalization of expression and rescue of transcallosal dysconnectivity via repressive chromatin remodeling.

## Introduction

A wide range of neurodevelopmental disorders manifesting in infancy and early childhood (including intellectual disability and autism spectrum disorder) or young adulthood (including schizophrenia) are associated with disrupted interhemispheric communication, which in some but not all of the affected cases is accompanied by structural alterations of the corpus callosum, the brain’s largest commissure^1–3^. The formation of interhemispheric connectivity involves a highly orchestrated multi-step process, including midline zipper glia promoting hemispheric fusion and midline crossing of pioneer fibers, followed by ingrowth of large numbers of transcallosal axons interconnecting the left and right cerebral cortex^4^. Early occurring disruptions of commissural development could manifest as agenesis of the corpus callosum^4^. However, alterations affecting later phases of development, including defective or misdirected axonal growth cone navigation with failure to cross the midline, or faulty axonal ingrowth into the contralateral cortex, could be responsible for the partial thinning, or developmental hypoplasia of the corpus callosum, extending either across its entire rostro-caudal axis or subportion thereof^4,5^. In addition, proper region- and lamina-specific axonal innervation and arborization within contralateral cortex are dependent on the functional activity of cortical projection neurons^6^. Furthermore, late occurring defects of transcallosal connectivity may not result in overt macroscopic defects of brain morphology including its commissures. Such a complex developmental program of commissural connectivity is, perhaps unsurprisingly, associated with a heavy footprint in the genetic risk architecture of neurodevelopmental disease. In fact, a number of chromosomal copy number variations (CNVs) have been linked to callosal agenesis^7^.

Of note, many cases with a chr. 22q11.2 micro-deletion or -duplication^8^ as one of the most frequently diagnosed CNV in neuropsychiatric disease cohorts are affected by callosal hypo- and hyperplasia^8^ and furthermore, interhemispheric dysconnectivity phenotypes have been associated with a rapidly increasing list of specific point mutations in cell surface signaling genes encoding key regulators of neurite outgrowth and connectivity, such as the *Roundabout guidance receptor 1* (*ROBO1*)^9^ or the *L1 cell adhesion molecule* (*L1CAM*)^10^. Much less is known about the role of nuclear signaling molecules for disease-relevant commissural phenotypes. For example, orderly formation of transcallosal connectivity critically depends on proper gene dosage and activity of a subset of chromatin regulators such as the transcriptional repressor *switch-insensitive 3 family member A* (*SIN3A*)^11^ and the *Forkhead box G1* (*FOXG1*) transcription factor^12^. However, molecular and cellular mechanisms linking the neuronal epigenome to interhemispheric connectivity remain poorly explored.

Here, we describe *C11orf46/ADP ribosylation factor like GTPase 14 effector protein* (*ARL14EP*), a small (35 kDa) nuclear protein encoded in the chr. 11p13 Wilms Tumor, Aniridia, Genitourinary Abnormalities, intellectual disability (formerly referred to as Mental Retardation) (WAGR) risk locus, as a novel chromatin regulator of transcallosal connectivity. Importantly, we show that C11orf46 is, as an RNA-guided Cas9 fusion protein, suitable for targeted epigenomic promoter editing to drive the expression of specific regulators of axonal development in immature transcallosal projection neurons, thereby offering a molecular tool to affect interhemispheric communication.

## Results

### C11orf46 is critical for transcallosal connectivity

A recent study identified 50 novel genes associated with autosomal recessive forms of intellectual disability, including open reading frame *C11orf46*^13^. Importantly, moreover, this gene is located in the chr. 11p13-14 neurodevelopmental risk locus and deleted in some cases of WAGR syndrome (Online Mendelian Inheritance of Man (OMIM) #194072), a rare copy number variant disorder with its core features caused by haploinsufficiency for the *Wilms tumor 1* (*WT1*) and *Paired box 6* (*PAX6*) homoebox gene^14^. Of note, *C11orf46* is positioned in between *PAX6* and the *Brain derived neurotrophic factor* (*BDNF*, chr. 11p14.1) gene which is included in the deleted segment in approximately half of patients with WAGR syndrome and associated with obesity and more severe cognitive and behavioral deficits^14^. However, there is evidence that, independent of *BDNF*, additional coding genes in chr. 11p13-14 could contribute to neurodevelopmental disease. For example, exome sequencing studies in Middle Eastern pedigrees associated homozygous *C11orf46* (chr11:30,323,051-30,338,458, GRCh38/hg38) point mutations with cognitive disease and intellectual disability^13^. To begin the neurological exploration of *C11orf46*, we studied differences in brain morphology between WAGR microdeletion cases encompassing the *C11orf46* gene and those with less extensive chr. 11p13 segment haploinsufficiency.

**Fig. 1:**
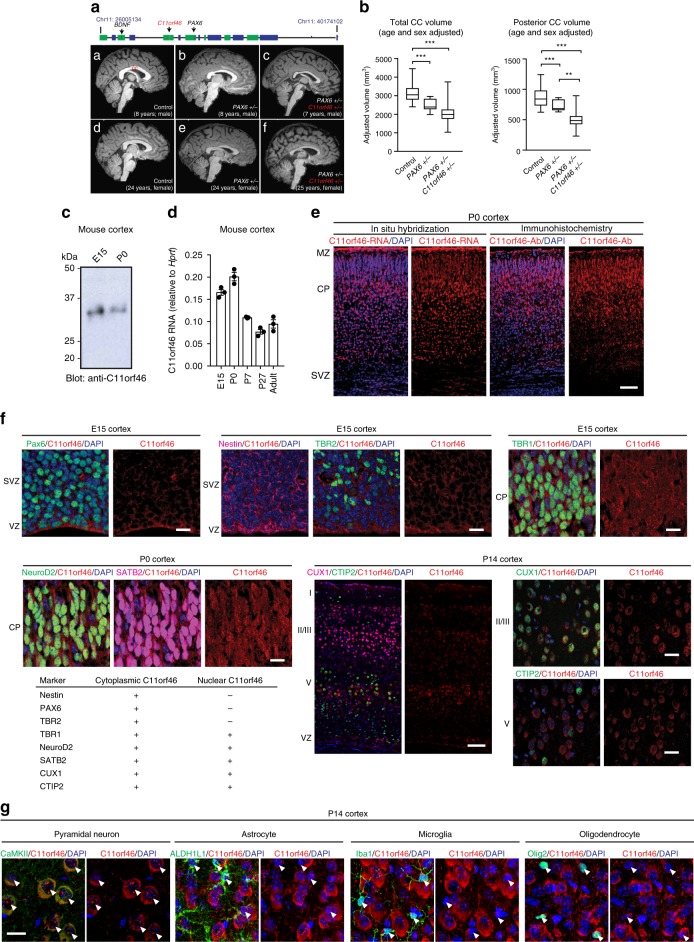
C11orf46 is a neuronal nuclear protein important for callosal development. **a** (top) Genomic map of chr. 11 WAGR deletion locus. (A–F) Representative sagittal T1 MRI images from midline were shown. A) 8-year-old male control; B) 8-year-old male isolated *PAX6+/−;* C) 7-year-old male heterozygous 11p13 deletion with *PAX6+/−* and *C11orf46+/−;* D) 24-year-old female control; E) 24-year-old female isolated *PAX6+/−;* F) 25-year-old female heterozygous 11p13 deletion with *PAX6+/−* and *C11orf46+/−*. **b** Hypoplastic corpus callosum (CC) in patients with 11p13 deletion encompassing *C11orf46*. ANCOVA including age and sex as covariates, compared CC volumes of control (*n* = 23), isolated *PAX6+/−* (*n* = 12), and heterozygous 11p13 deletion with *PAX6+/−* and *C11orf46+/−* (*n* = 27). *P*-values are shown from two-sided post hoc LSD comparisons: **P < 0.01, ***P < 0.001. Box and whisker plots represent the distribution of unadjusted CC volume. The inner bar indicates median value. The upper and lower box ends represent first and third quantile. The upper and lower whisker ends represent the maximum and minimum values. **c** C11orf46 protein in mouse cortices at embryonic day 15 (E15) and postnatal day 0 (P0). **d** mRNA expression of C11orf46 in the mouse cerebral cortex during prenatal and postnatal development (mean ± SEM). *n* = 3. **e** (Left) *C11orf46* mRNA (red) is predominantly expressed in the cortical plate (CP) in the somatosensory cortex at P0. (Right) C11orf46 protein (red) is predominantly expressed in the CP at P0. Blue, nucleus. Scale bar, 50 µm. MZ, marginal zone; SVZ, subventricular zone. **f** C11orf46 (red) is expressed in the cytoplasm, but not in the nucleus in Nestin-, PAX6-, and TBR2-positive cells, whereas C11orf46 is expressed in the cytoplasm and in the nucleus with a punctate pattern in TBR1-, NeuroD2-, SATB2-, CUX1-, and CTIP2-positive neuronal cells. (bottom left) Cell type specific C11orf46 expression are summarized in the table. Scale bar, 10 and 100 μm (low magnification images). **g** Representative images of C11orf46 protein staining with cell type-specific markers (white arrowheads) at P14. C11orf46 is predominantly expressed in CaMKII-positive neurons, but not Olig2 (oligodendrocyte), ALDH1L1 (astrocyte), Iba-1 (microglia) -positive cells. Scale bar, 20 μm

To this end, we examined magnetic resonance T1-weighted images (MRI) in health control subjects (*n* = 23), isolated *PAX6+/−* subjects (*n* = 12), and individuals with WAGR-region deletions involving *PAX6+/−* and *C11orf46+/−* (*n* = 27). Indeed, *C11orf46* haploinsufficiency was associated with significant differences observed in total corpus callosum (CC) and posterior CC volumes but not for other individual portions of the CC. After adjusting for age and sex, patients with isolated *PAX6*+/− compared to controls had significantly smaller total and posterior CC volumes [ANCOVA: F(4,57) = 29.43, *P* < 10^−8^; post hoc two-sided LSD mean difference (95% CI): −705 (−1046 to −364) mm^3^, *P* = 0.0001] and posterior CC volumes [*F*(4,57) = 36.38, *P* < 10^−10^; −180 (−282 to −78) mm^3^, *P* = 0.0009], a finding consistent with the role of *PAX6* in morphologic brain development^15,16^. However, patients with combined *PAX6/C11orf46*+/− had an even smaller posterior CC volume compared to both patients with isolated *PAX6*+/− [−173 (−284 to −62) mm^3^, *P* = 0.003] and controls [−353 (−436 to −270) mm^3^, *P* < 10^−10^] (Unadjusted volumes are shown but *P*-values from ANCOVAs adjusting for covariates are depicted in the figure; estimated marginal means are provided in the figure legend) (Fig. 1a, b). Because of the established role of *BDNF* in brain development and intellectual functioning^17^, we also considered a potential confounding effect of *BDNF+/−*. We observed no significant differences in the *PAX6/C11orf46*+/− patients with (*n* = 17) and without (*n* = 10) *BDNF+/−* for total or posterior CC volumes [246 (−122 to 614) mm^3^, *P* = 0.19 and 41 (−73 to 156) mm^3^, *P* = 0.47, respectively, for unadjusted comparisons; 281 (−84 to 647) mm^3^, *P* = 0.13 and 50 (−61 to 162) mm^3^, *P* = 0.37, respectively, on ANCOVA adjusting for age and sex] (Supplementary Fig. 1a). Lymphoblastoid cell lines from *C11orf46* haploinsufficient WAGR cases have approximately 50% lower C11orf46 mRNA levels, indicating that the expression of the remaining intact allele does not undergo compensatory upregulation (Supplementary Fig. 1b). Therefore, loss of one *C11orf46* copy in the context of chr. 11p13-14.1 microdeletions appears to be more highly detrimental for neurodevelopment and associated with more severe hypoplasia of the callosal commissure.

Having linked *C11orf46* haploinsufficiency to callosal hypoplasia, we next explored C11orf46 expression patterns in mouse brain. In the cerebral cortex, C11orf46 quantification at the level of mRNA, and protein with a custom-made anti-C11orf46 antibody (Supplementary Fig. 1c) showed its expression across a wide age window, with higher levels in the pre- and post-natal developmental periods as compared to the adult brain (Fig. 1c–f). C11orf46 was mainly expressed in the cortical plate (CP) with minimal labeling in the proliferative subventricular zone (SVZ) at mRNA and protein levels (Fig. 1e). To examine cell type-specific expression pattern of C11orf46 during brain development, C11orf46 was co-stained with multiple cell type-specific markers. Notably, C11orf46 is expressed in a nuclear punctate pattern in NeuroD2-positive cells, but not in PAX6 and TBR2-positive cells, although we observed cytoplasmic expression of C11orf46 in cells expressing these markers (Fig. 1f and Supplementary Fig. 2a). Nuclear and cytoplasmic expression of C11orf46 was also observed in neuronal markers, such as TBR1, SATB2, CUX1, and CTIP2-positive cells (Fig. 1f and Supplementary Fig. 2a). In Nestin-expressing radial glial cells, C11orf46 is expressed in the cytoplasm, but not in the nucleus (Fig. 1f and Supplementary Fig. 2a). Collectively, these results suggest that nuclear C11orf46 is primarily expressed in post-mitotic neurons. Additional double-labeling experiments confirmed that C11orf46 is predominantly expressed in Calmodulin-kinase II (CaMKII)-positive glutamatergic neurons but not in AlDH1L1-positive astrocytes or Olig2-positive oligodendrocytes and precursors, or Iba1-positive microglia (Fig. 1g and Supplementary Fig. 2b). C11orf46 immunoreactivity in GABAergic neurons was much weaker than those in glutamatergic neurons (data not shown). We conclude that in the developing cerebral cortex, C11orf46 is primarily expressed in post-migratory glutamatergic cortical neurons.

**Fig. 2:**
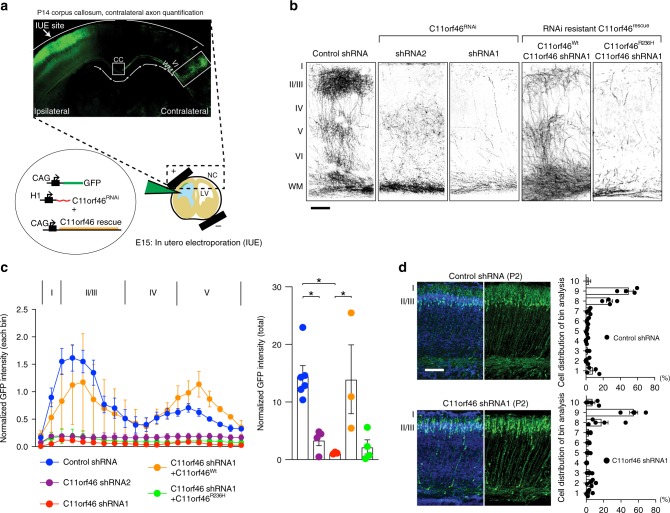
Suppression of C11orf46 impairs callosal development and interhemispheric connectivity. **a** (top) Schematic representation of in utero electroporation of GFP expressing plasmid into somatosensory cortex at E15. (bottom) P14 somatosensory cortex expressing GFP in pyramidal neurons at IUE injection site (Ipsilateral side) and axonal projections between layer I–VI (Contralateral side). **b** Knockdown of C11orf46 in pyramidal neurons of layer II/III (Ipsilateral) show impaired axonal terminal arborization in the contralateral somatosensory cortex at P14. C11orf46 shRNA1 had a stronger axonal arborization impairment than shRNA2 when compared with control shRNA (first 3 panels); this axonal deficit was partially rescued by co-expressing RNAi resistant wild-type C11orf46 (C11orf46^Wt^), but not by R236H mutant (C11orf46^R236H^) (last 2 panels). Scale bar, 100 µm. **c** Layer distributions (left) and total axonal density (right) of callosal axons in the contralateral site of the electroporated brains were shown as normalized immunofluorescence intensities of callosal axons. *F* (4, 15) = 7.9591, *P* = 0.0012 was determined by one-way ANOVA with post hoc Bonferroni test. Three to six mice per condition. *P < 0.05. Impaired axon terminal arborization across all cortical layers elicited by C11orf46 knockdown was restored by overexpression of C11orf46^Wt^, but not by overexpression of C11orf46^R236H^. **d** C11orf46 knockdown did not have an effect on radial neuronal migration at P2. Scale bar, 50 μm. Bar graphs indicate mean ± S.E.M (**c**, **d**)

To explore the role of C11orf46 in developing cortex, we knocked down its expression by in utero electroporation (IUE). We delivered C11orf46 (shRNA1, shRNA2) or control short hairpin RNAs (shRNAs) together with green fluorescent protein (GFP) expression plasmid using our published protocols^18–21^. We selected embryonic day 15 (E15) for IUE procedure with two reasons: first, C11orf46 is highly expressed at E15 (Fig. 1c, d). Second, at this stage IUE mostly targets progenitor cells in the ventricular zone (VZ) which later differentiate into cortical layer II/III pyramidal neurons^22,23^, many of which become transcallosal projection neurons^24^ as our cell type-of-interest given the clinical phenotype reported above. Indeed, E15 IUE *C11orf46* knockdown resulted in severe arborization deficits in GFP-labeled callosal axons projecting into the contralateral cortex across all cortical layers at postnatal day 14 (P14) (Fig. 2a–c). This phenotype was highly dependent on the level of *C11orf46* knockdown. More severe axonal arborization deficits by C11orf46 shRNA1 (Fig. 2b, c) was associated with stronger knockdown effect on C11orf46 compared with shRNA2 (Supplementary Fig. 3a). Importantly, these axonal arborization deficits were partially rescued by co-expressing shRNA resistant wild-type C11orf46. Strikingly however, an shRNA resistant C11orf46 (R236H) mutant protein carrying a disease-associated non-synonymous point mutation that consists of arginine to histidine substitution at codon 236^13^ did not rescue the transcallosal phenotype induced by *C11orf46* knockdown (Fig. 2b, c and Supplementary Fig. 3b). We also found reduction of thickness of corpus callosum that was elicited by knockdown of *C11orf46*, which is reversed by co-expression of RNAi-resistant wild-type C11orf46, but not by C11orf46 (R236H) mutant (Supplementary Fig. 3c). Furthermore, we observed no difference in percentage of SATB2 and CUX1 positive cells in GFP-labeled neurons in *C11orf46* knockdown brains, compared to those in controls, suggesting that *C11orf46* knockdown did not affect the number of cortico-cortical projecting neurons (Supplementary Fig. 3d). Importantly, the observed axonal phenotype resulting from C11orf46 knockdown was highly specific, because orderly radial neuronal migration into the cortex, when assessed at P2, was indistinguishable from control (Fig. 2d).

### C11orf46 regulates genes important for axonal connectivity

**Fig. 3:**
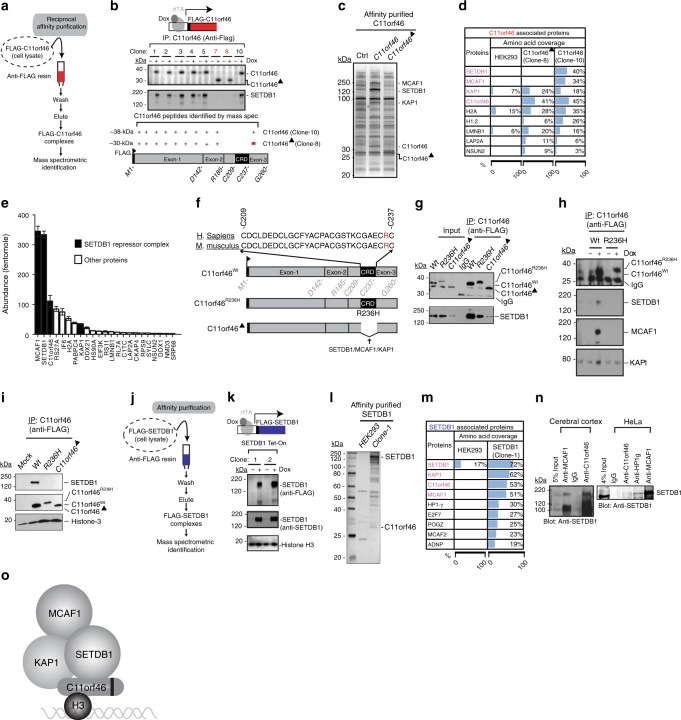
C11orf46 binds to the SETDB1 complex. **a** Flow diagrams for immunoaffinity purification of C11orf46 followed by mass spectrometric analysis. **b** Eight clones of inducible HEK293 cell lines expressing FLAG-tagged C11orf46 (clone 1–5 and 10) and a truncated form C11orf46 lacking amino acids 209–237 at the C-terminal region which corresponds to cysteine rich domain (CRD) of C11orf46 (C11orf46∆) (clone 7 and 8) in the presence of doxycycline (Dox+). SETDB1 was co-precipitated with C11orf46, but not with C11orf46∆. **c** Silver staining of proteins co-precipitated with C11orf46 (clone 10) or C11orf46∆ (clone 8). SETDB1, MCAF1 and KAP1 were detected in co-precipitate from C11orf46, but not of C11orf46∆. **d** Top scoring proteins co-precipitated with C11orf46 in cell lines overexpressed with C11orf46, C11orf46∆, and control cells are shown with % of amino acid sequence coverage. **e** Protein abundance in affinity purified C11orf46 complexes. Notice prominence of SETDB1-MCAF1-KAP1 repressor proteins (black). Bar graphs indicate mean ± S.E.M. *n* = 3 independent quantification. **f** Schematic diagrams indicating position of C11orf46 arginine to histidine substitution at codon 236 (R236H) in conserved cysteine rich domain (CRD; amino acids 209–237) between human and mouse. **g**, **h** Co-immunoprecipitation experiments using protein lysates from HEK293 cells overexpressing FLAG-tagged C11orf46, C11orf46∆, or C11orf46^R236H^ showed that absence of CRD domain and R236H mutation is associated with weakened or non-detectable C11orf46-SETDB1 binding and partial loss of binding to other components. R236H mutation does not fully disrupt C11orf46-KAP1 binding. **i** R236H mutation and CRD deletion of C11orf46 do not affect binding to histone H3. **j** Flow diagrams for immunoaffinity purification of SETDB1 followed by mass spectrometric analysis. **k** Inducible SETDB1 expression system in HEK293 cell lines (48 h after Dox treatment). **l**, **m** Silver staining of proteins co-precipitated with SETDB1. Both endogenous C11orf46 and SETDB1 binding partners were detected by mass spectrometry. Top scoring proteins co-precipitated with SETDB1 in cell lines overexpressed with SETDB1 and control cells are shown. **n (**left) C11orf46-SETDB1 co-immunoprecipitation in human cerebral cortex. (right) SETB1 co-immunoprecipitates include C11orf46, HP1γ, and MCAF1 in HeLa nuclear extract. **o** Schematic summary of above data representing working model of C11orf46-SETDB1 complex in relation to histone H3

Having shown that C11orf46 is widely expressed in cortical projection neurons and essential for transcallosal connectivity, we next wanted to gain initial insights into the molecular mechanisms underlying C11orf46 actions on the corpus callosum development. In the single report published to date on C11orf46 function, the protein was renamed ADP-Ribosylation Factor-Like 14 Effector Protein (ARL14EP), and ascribed with a role in major histocompatibility class (MHC) II antigen presentation in immune cells^25^. Therefore, we were very surprised about the predominant, if not exclusive, nuclear localization of C11orf46 in our immunohistochemical assays in the developing CP (Fig. 1e, f). However, a recent study listed C11orf46/ARL14EP among several hundred proteins serving as nuclear export cargo for the RanGTPase-driven exportin CRM1^26^ and moreover, large scale total proteome protein–protein interaction mappings consistently identified chromatin-regulatory proteins as C11orf46 binding partners, including the SET domain bifurcated histone lysine methyltransferase 1 (SETDB1)/KMT1E (SETDB2/KMT1F)-MBD-containing-associated factor (MCAF1) histone lysine 9 (K9) methyltransferase repressor complex^27–30^ (referred to as KMT-RC hereafter). To further examine C11orf46-associated proteins by affinity purification coupled to mass spectrometry (Fig. 3a), we generated inducible HEK293 cell lines expressing Flag-tagged C11orf46 (Fig. 3b). We produced two types of inducible clones, one for full length FLAG-tagged C11orf46 (clones 1–5 and 10) and one for a truncated form lacking amino acids 209–237 at C11orf46’s C-terminal region which corresponds to cysteine rich domain, CRD, (C11orf46∆) (clone 7 and 8) (Fig. 3b and Supplementary Fig. 4a, b). Interestingly, nuclear proteins outranked all other C11orf46 binding partners, with the top scoring SETDB1-KRAB associated protein (KAP1)-MCAF1 chromatin repressor complex^31,32^ (Fig. 3c–e and Supplementary Table 1), which is consistent with proteome interaction databases^27–30^. These findings were specific for full length C11orf46 because the truncated form C11orf46∆ showed only very weak, or no interactions with nuclear proteins including the SETDB1-KAP1-MCAF1 repressor complex (Fig. 3d). Given the *C11orf46*^*R236H*^ point mutation is located in the CRD domain which is required for protein binding of C11orf46 with SETDB1 (Fig. 3b–d), we wondered whether R236H may affect the interaction of C11orf46 and the SETDB1 complex. To test this hypothesis, in addition to inducible HEK293 cell lines expressing Flag-tagged C11orf46 and Flag-tagged C11orf46∆, we generated cell lines for inducible expression of FLAG-C11orf46^R236H^ (Fig. 3f). We observed that deletion of CRD domain dramatically weakened the interaction of C11orf46 and SETDB1, whereas the R236H substitution abolished C11orf46-SETDB1 interaction (Fig. 3g, h). Binding of C11orf46 with MCAF1 and KAP1, other members of the SETDB1 protein complex, were also severely decreased by R236H substitution (Fig. 3h). Notably, both C11orf46 with R236H mutation and that with deletion of CRD domain still kept binding affinity with histone H3 (Fig. 3i). Also, purified, *E*. *coli* derived, human C11orf46 specifically detected histone H3 tail modifications in a histone peptide array (Supplementary Fig. 4c–e). Similarly, reciprocal affinity purification with FLAG-tagged SETDB1 using inducible cell lines confirmed C11orf46 as a top ranking binding partner together with other members of the complex, including KAP1 and MCAF1 (Fig. 3j–m and Supplementary Table 2). Additional co-immunoprecipitation experiments with mouse anti-C11orf46 antibody in HeLa cells, and in homogenates of human cerebral cortex confirmed that C11orf46 assembles with SETDB1, MCAF1 and other regulators of repressive chromatin, including heterochromatin-associated protein 1gamma (HP1γ) (Fig. 3n and Supplementary Fig. 4f). These results suggest that C11orf46 may function as a chromatin regulator in the SETDB1 complex (Fig. 3o).

### Suppression of *C11orf46* altered expression of axonal genes

**Fig. 4:**
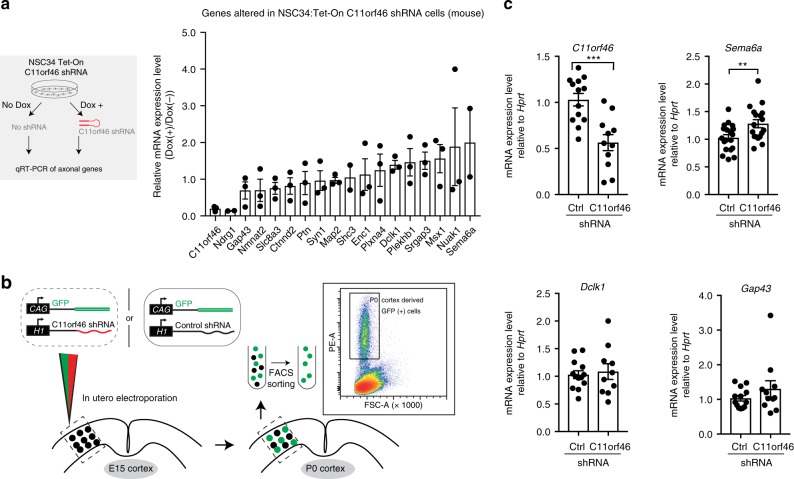
Selection of targeted genes by C11orf46-SETDB1 complex. **a** Mouse neuroblastoma cells NSC34 inducibly expressing C11orf46 shRNA upon doxycycline addition (schematic in left panel); selected subset of 17 differentially expressed genes implicated in axonal growth and development was tested in RNA expressed from NSC34:Tet-On C11orf46 shRNA cell (right panel). **b** Neuronal progenitors received control or C11orf46 shRNA and GFP marker plasmids at somatosensory cortex by IUE at E15; four days later at P0 GFP-positive cells were collected by fluorescence-activated cell sorting (FACS). **c** *C11orf46* transcript levels were reduced by 50% in sorted GFP-positive cortical neurons electroporated with C11orf46 shRNA1 as compared to GFP-positive neurons with control shRNA (P = 0.0003). Note increase of *Sema6a* transcript upon C11orf46 knockdown (*P* = 0.0085). *P < 0.05, **P < 0.01, ***P < 0.001 determined by Student’s *t*-test test. *n* = 10–19 independent mice per condition. All data were normalized by the *Hprt* transcript. Bar graphs indicate mean ± S.E.M (**a**, **c**)

Having shown that C11orf46 is a neuronal nuclear protein bound to repressive chromatin regulators, we hypothesized that the trans-callosal dysconnectivity resulting from *C11orf46* knockdown in fetal mouse cortex and of *C11orf46* deletions in WAGR patients could reflect dysregulated expression of genes critical for axonal growth and development. In order to obtain first insights into C11orf46-sensitive gene expression changes in WAGR deletion cases, we analyzed a RNA-seq dataset of lymphoblastoid cell lines obtained from the aforementioned 2 WAGR cases with *C11orf46* haploinsufficiency compared to 2 controls, including one WAGR case with a shorter microdeletion that retained intact *C11orf46* and one healthy control subject with no loss of genes for the entirety of chr. 11p13-14.1 (Supplementary Fig. 1b). We identified 227 differentially expressed genes (182 down-regulated genes and 45 upregulated genes) (FDR < 0.05, see Methods; Supplementary Fig. 5 and Supplementary Table 3) that include 41 genes which have KAP1 binding sites^33^ (Supplementary Table 4). Notably, among these 41 genes, we found that 17 genes are reportedly involved in axonal development (Fig. 4a, Supplementary Table 4 genes marked in bold), while the remaining 24 genes, such as CAP2 encoding an adenylyl-cyclase associated protein, are linked to various other cellular functions (Supplementary Table 4). However, given that the observed gene alterations were likely confounded by deletion of other proximal genes with *C11orf46* at WAGR locus, we switched in our next set of experiments to neuron-like (neuroblastoma) NSC34 Tet-On cells expressing C11orf46 shRNA to further examine C11orf46-regulated genes. Nonetheless, we found that expression levels of the 17 axonal growth and development genes were not consistently affected in response to C11orf46 shRNA-mediated *C11orf46* knockdown in the neuroblastoma cell line. However, multiple putative C11orf46 target genes, including *Semaphorin 6a* (*Sema6a*), *Doublecortin-like kinase 1* (*Dclk1*), *SLIT-ROBO Rho GTPase activating protein 3* (*Srgap3*), and others showed increased expression after *C11orf46* knockdown in the NSC-34 (Fig. 4a). Therefore, we wanted to confirm in neurons, in vivo, C11orf46’s role as a transcriptional regulator during brain development, focusing on the three candidate genes with KAP1 binding sites proximal to transcription start site (TSS) (*+/−*750b)^33^, including two genes (*Sema6a*, *Dclk1*) with expression changes in response to *C11orf46* knockdown in the NSC-34. *Dclk1* is essential for axon tract formation across the anterior commissure in the ventral forebrain and the corpus callosum^34^, *Sema6a* encodes a PLXNA4 protein ligand^35,36^, and our third candidate gene, *Gap43*, encodes a growth cone protein essential for commissural axon guidance^37^ (Supplementary Table 4). To confirm C11orf46’s role as a transcriptional regulator during brain development, we knocked down *C11orf46* by IUE-mediated delivery of C11orf46 shRNA together with GFP expression plasmid at E15 and GFP-labeled projection neurons were isolated by fluorescence-activated cell sorting (FACS) at P0 (Fig. 4b). Notably, *Sema6a* was significantly increased in *C11orf46* knockdown projection neurons (Fig. 4c). These findings, taken together, strongly suggest that C11orf46 regulates neuronal gene expression, including an inhibitory effect on *Sema6a*.

### C11orf46- epigenome editing rescues a callosal phenotype

**Fig. 5:**
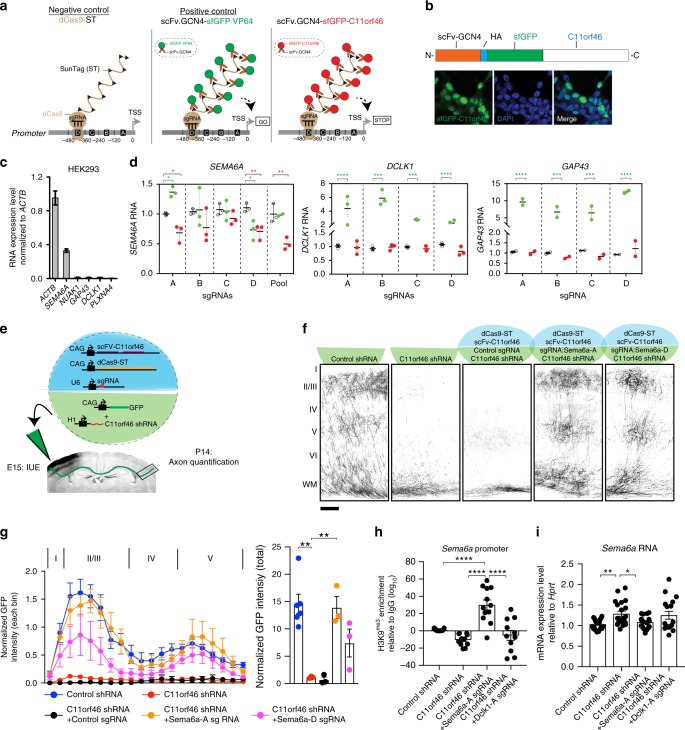
C11orf46- epigenomic editing of neurite-regulating genes rescues transcallosal dysconnectivity in C11orf46-deficient neurons. **a** Schematic representation showing epigenome editing using nuclease-deficient CRISPR/Cas9 SunTag (dCas9-ST) system. Single guide RNA (sgRNA) targeting neuronal gene promoters is introduced with dCas9-ST (10xGCN4) and scFv-sfGFP-C11orf46 (or −VP64 as a positive control) in HEK293 cells. **b** Overexpressed fGFP-C11orf46 is colocalized with DAPI signals in HEK293 cells. **c** Relative expression levels of neurite-regulating genes in HEK293 cells. **d** dCas9-ST mediated promoter loading with 10xC11orf46, and C11orf46^R236H^ in comparison to 10xVP64 at *SEMA6A* in HEK293 cells. Four sgRNAs (**a**–**d**) for each gene were tested individually, and for *SEMA6A* also as a pool. Two-way ANOVA followed by Dunnett’s multiple comparisons test was performed where C11orf46- or VP64-epigenetic editing effects were tested. **e** Epigenomic editing of transcallosal neurons by IUE. Five transgenes were delivered simultaneously at E15 by IUE into the developing somatosensory cortex, followed by quantification of axonal arborization at P14. **f**, **g** Axonal arborization was disrupted by *C11orf46* knockdown, and restored by dCas9-ST mediated recruitment of C11orf46 to *Sema6a* promoter (Sema6a-), using sgRNAs Sema6a-A, Sema6a-D, but not with non-targeting sgRNA. Scale bar, 100 µm. Layer distributions and total axonal density in the contralateral site of the electroporated brains are shown (**g**). *F* (4, 13) = 13.1934, *P* = 0.0002 was determined by one-way ANOVA with post hoc Bonferroni test. Three to six mice per condition. **h** H3K9me3 enrichment at *Sema6a* promoter in GFP + neurons at P0, four days after IUE. Epigenome editing by Sema6a-A sgRNA increases H3K9me3 levels at *Sema6a* promoter. Dclk1-A sgRNA did not affect H3K9me3 levels at *Sema6a* promoter. *F* (3,44) = 8.718, *P* = 0.0001 was determined by one-way ANOVA with post hoc Bonferroni test. **i** *Sema6a* mRNA level in GFP + neurons at P0. Increased *Sema6a* expression caused by *C11orf46* knockdown were suppressed by C11orf46-epigenome editing using Sema6a-A sgRNA, but not by Dclk1-A sgRNA. *F* (3,72) = 4.746, *P* = 0.0045. *P < 0.05, **P < 0.01 determined by one-way ANOVA with post hoc Bonferroni test. *P < 0.05, **P < 0.01, ***P < 0.001, and ****P < 0.0001. Bar graphs indicate mean ± S.E.M.

Our results, reported above, showed that C11orf46 co-assembles with the KMT-RC. Apparently, this interaction is important because mutations such as *C11orf46*^*R236H*^, disrupting C11orf46’s association with the complex, are highly detrimental to orderly brain development. We hypothesized that C11orf46’s affinity to the KMT-RC could be exploited, in order to ‘edit’ neuronal gene expression via a chromatin-associated mechanism. Such type of epigenome editing could be accomplished, for example, by RNA-guided fusion proteins comprised of nuclease-deficient clustered, regularly interspaced short palindromic repeats (CRISPR)/CRISPR-associated protein 9 (dCas9) bound to a transcriptional activator or repressor^38^. However, because ‘1:1’ systems (one transcriptional regulator per dCas9 copy) are often only minimally effective^38,39^, we instead used dCas9-SunTag protein scaffolds which assembles as a binary system with the protein of interest fused to a single-chain antibody variable fragment (scFv)-GFP cassette, with the scFv binding to the Cas9-fused protein scaffold carrying 10 or more copies of GCN4 (the scFv epitope)^39^. We built a dCas9-SunTag system to load ten copies of C11orf46, or the VP64 activator protein as a positive control, onto a single sgRNA-target sequence (Fig. 5a). We began with our epigenome editing experiments in HEK293 cells selecting *Sema6a*, *Dclk1*, and *GAP43*. For each gene, we obtained four sgRNAs positioned within 500 bp upstream of the target TSS. For each of the three target genes, three groups of HEK293 cells were compared, based on transfection with (1) dCas9-ST alone (sgRNA-dCas9-10xGCN4^’SunTag’^) or dCas9-ST together with one of the following plasmids, (2) scFv which recognizes the GCN4 epitope on dCas9-ST-superfold GFP-VP64, or (3) scFv-superfold GFP-wild-type C11orf46 (C11orf46^wt^) (Fig. 5a). After nuclear localization of the scFv-sfGFP-C11orf46 domain was confirmed (Fig. 5b), testing for each target gene experiments were conducted in parallel in HEK293 cells, with each of the four sgRNA tested individually (and in a pool of four for *SEMA6A* as well). Interestingly, introduction of two of the four sgRNAs to *SEMA6A* which is expressed at much higher levels as compared to the other test genes (Fig. 5c), resulting in significant decrease in *SEMA6A* expression when co-transfected with the dCas9-ST^10x,C11orf46(wt)^ systems (Fig. 5d). Conversely, *DCLK1* and *GAP43*, which is expressed at extremely low levels at baseline (Fig. 5c), showed minimal changes when co-transfected with dCas9-ST^10x,C11orf46(wt)^, while responding to dCas9-ST^10*x*VP64^ with a robust, at least 5–10 fold increase in their expression (Fig. 5d).

Our studies in HEK293 cells suggest that C11orf46, when tethered in multiple copies to specific promoter sequences via the CRISPR-Cas9-SunTag system, could actively repress transcribed genes. Next, we wanted to explore this approach in vivo in developing upper layer cortical projection neurons of somatosensory cortex. We focused on *Sema6a*, which was de-repressed after *C11orf46* knockdown in NSC34 cells and GFP-positive neurons (Fig. 4a, c). Both *Sema6a* and *C11orf46* are highly expressed in upper cortical layers II/III at P0, within the targeted time of brain development, via IUE (Supplementary Fig. 6a). Two sgRNAs, targeting different sequences within the first 500 bp upstream from the TSS of *Sema6a*, were selected for in vivo studies and delivered at E15 (Fig. 5e). Strikingly these *Sema6a* promoter-targeting sgRNAs rescued impaired midline crossing of callosal projections and axonal arborization deficit normally encountered upon *C11orf46* knockdown at E15 by IUE (Fig. 5f, g and Supplementary Fig. 6b). Consistent with C11orf46’s association with KMT-RC, introduction of *Sema6a* sgRNA also resulted in increased H3K9me3 levels at the *Sema6a* promoter, in striking contrast with a decrease in H3K9me3 levels after *C11orf46* knockdown (Fig. 5h). These effects were highly specific for the target gene sequence, because sgRNAs directed against *Dclk1* promoter did not affect the *Sema6a* promoter (Fig. 5h). Likewise, increased *Sema6a* expression in *C11orf46* knockdown neurons was strongly suppressed by our SunTag-based C11orf46-mediated epigenome editing toolkit targeting the *Sema6a*, but not the *Dclk1* promoter (Fig. 5i). Consistently, overexpression of *Sema6a* elicited disturbance of midline crossing of callosal projections and axonal terminal arborization in the developing cerebral cortex (Supplementary Fig. 6c).

## Discussion

Axonal development is a pivotal process in establishing neural connectivity for brain functions, including cognition and memory^40^. This critical stepwise process coordinately regulates the establishment of synaptic connections to target sites with topographic specificity^41^. Disrupting these processes may lead to altered neural connectivity, which may underlie cognitive disabilities in neurodevelopmental psychiatric conditions such as intellectual disability and autism spectrum disorder^42^. While multiple molecular pathways including guidance cues and growth factors have been implicated in axonal development^41^, very little is known about chromatin-regulatory mechanisms^43^. Recently, the SET (histone methyltransferase) domain-containing transcriptional repressor, PR/SET domain 8 (PRDM8), was described as essential for the formation of trans-callosal and other interhemispheric fiber tracts^44^ Likewise, Heterochromatin-associated protein 1gamma (HP1γ) is required for the crossing of callosal axons into the contralateral hemisphere, while increased neuronal HP1γ expression promotes growth and differentiation of dendrites and axons^45^. Interestingly, PRDM8 promotes H3K9 methylation^46^ and HP1γ is bound preferentially to compacted nucleosomal arrays bearing the repressive H3K9me3 mark^47^. Furthermore, the C11orf46-binding partner and repressive H3K9MTase, SETDB1, has been implicated in transcriptional regulation of S-type clustered Protocadherin genes, a group of cell adhesion molecules broadly important for neuronal connectivity^48^. These findings, when taken together with our results reported here, imply H3K9 methyl-regulators as important epigenomic determinant of the developing cortical connectome.

In this study, we showed that C11orf46 assembles with bona fide factors of histone-3 lysine-9 trimethyltransferase (H3K9me3) SETDB1 complex, it substrate histone H3 and a subset of nuclear proteins functionally associated with repressive and heavily H3K9-methylated (hetero) chromatin, including Lamin B1 (LMNB1), Lamina-associated protein 2, isoform alpha (LAP2A) and HP1γ. These multi-molecular assemblies of chromatin-bound proteins presumably require distinct domains of C11orf46 to associate with SETDB1 complex and histone H3. Importantly, non-synonymous genetic mutations G218E and R236H found in the CRD domain of C11orf46 are associated with neurodevelopmental disease conditions^13,49^. We show that specifically the R236H, disrupted the C11orf46 assembly with the SETDB1 complex but not C11orf46’s association with histone H3. Therefore, mutations in critical domains of C11orf46 could disrupt the above assembly and potentially interfere with spatiotemporal gene silencing required for orderly development of transcallosal connectivity. Notably, purified C11orf46 showed strong affinity for histone H3 tail modifications in vitro; we were surprised to observe C11orf46’s high affinity towards a histone mark antagonizing histone methylation, H3R2-citrulline (H3R2Cit)^50,51^. Future studies will explore in more detail the regulatory role for C11orf46. For example, C11orf46 could sense the levels of mono- or di-methylated H3K9 to promote trimethylation, or block H3K9 methylation in response to high levels of antagonizing H3R2Cit mark.

In addition to protein components of the SETDB1 complex, such as KAP1 and MCAF1, our results of proteome screening identified LMNB1 and LAP2A, two nuclear lamina proteins that were previously discovered at the heterochromatin rich nuclear periphery^52^, as potential protein interactors of C11orf46. Interestingly, LMNB1 and LAP2A play critical roles for developmentally regulated dendritic and axonal connectivity^53^. Because C11orf46 resulted in impaired axonal development, but had no robust effect on dendritic structure, it will be important to further investigate epigenetic mechanisms to determine how C11orf46 specifically regulates axonal development.

To the best of our knowledge, this is the first study demonstrating the utility of the nuclease-deficient CRISPR/Cas9 system-mediated epigenome editing with IUE knockdown approach for addressing cell-autonomous epigenetic machinery for regulation of transcallosal connectivity. By targeting transcallosal projection neurons, we demonstrate that C11orf46 regulates axonal terminal arborization by control of expression of *Sema6a* via epigenomic promoter H3K9-methylation. The corpus callosum phenotypes in WAGR cases with *C11orf46* haploinsufficiency (Fig. 1) and the successful epigenomic rescue by sequence-specific C11orf46-mediated chromatin remodeling (Fig. 5), would imply that future chromatin-based therapies could be harnessed to correct developmental errors in the brain’s connectome. Importantly, genetic deletion of *Sema6a* resulted in impaired neuronal connectivity^54,55^. SEMA6A is a transmembrane protein and is known as a ligand for Plexin A2 (PLXNA2) and Plexin A4 (PLXNA4) which also play critical roles for regulating axon guidance^56^. *Plxna2* and *Plxna4* knockout mice showed impaired axonal development similar to those observed in mouse models with genetic deletion of *Sema6a*^56^. Thus, dysregulation of C11orf46-mediated control of *Sema6a* expression may impair axonal development regulated by Plxns either in cell autonomous or non-autonomous manner, which warrants further investigation in the future.

Our results, however, should carefully be interpreted for several reasons. First, although IUE-knockdown approach is a powerful method to examine the cell-autonomous effect of C11orf46 in the specific time critical for axonal growth, off-target effect of shRNAs might be confounded in the phenotypes^57^. To ensure that observed results do not originate from their off-target effects, we confirmed dose-dependent effects on the axonal phenotypes induced by two shRNAs with independent target sequences. Rescue experiments by co-expression of RNAi-resistant wild-type *C11orf46*, containing silent mutations in the target sequence of their shRNA, also ensured that the observed phenotypes are specifically due to its knockdown effect. Nevertheless, C11orf46-mediated mechanisms for axonal development and resulting WAGR syndrome-related phenotypes should be investigated in mice with genetic deletion of *C11orf46*, which will be considered for future studies. Second, we utilized RNA-seq data obtained from lymphoblastoid cell lines to identify differentially expressed genes involved in axonal development in WAGR cases, compared to those in controls. Lymphoblastoid cells may not represent brain-specific epigenome. Nevertheless, lymphoblastoid cells remain to be a convenient and well-characterized tool to elucidate brain disease-related molecular phenotypes^58^. Importantly, our RNA-seq data sets with matched controls revealed not only lymphoid genes, but also genes critical for neuronal function, representing the disturbances of neuronal-like epigenome in these lymphoid cells. Third, the discrepancy of *SEMA6A* expression in lymphoblastoid cells and neuronal conditions likely arise from the confounding effect of other genes located in the WAGR risk locus, including *PAX6* transcription factor. Epigenomic signatures of lymphoid cell line as well as the developing brain may also contribute to this discrepancy. Fourth, because human subjects in our study were recruited on the basis of having aniridia, all participants were, by definition, *PAX6* haploinsufficient, and, therefore, we were unable to assess the effect of *C11orf46+/−* independent of *PAX6+/−* and the phenotype of isolated *C11orf46+/−* is unknown (although homozygous mutations in *C11orf46* have been reported in association with intellectual disability). PAX6 is a transcription factor regulating neurogenesis and rostrocaudal patterning during development^59,60^. *PAX6* haploinsufficiency is associated with corpus callosum hypoplasia in both rodents and humans^15,16,61,62^. Thus, there remains the possibility that additional loss of *PAX6+/−* may be required for *C11orf46+/−* to cause significant defects in morphologic brain development. However, we were able to demonstrate that patients with combined loss of both genes had more severely reduced corpus callosum volumes compared to isolated *PAX6+/−*, confirming an additional role of C11orf46 in neurodevelopment. Whether the impact of mutations in the above molecules on axonal development is additive or synergistic remains to be determined. In addition, because the WAGR CNV encompasses a large genomic regions that encompass many genes, hampering identification of genetic drivers responsible for specific phenotypes shown in CNVs-associated disease conditions, our data do not exclude the possibility that other genes in 11p13 deletion region, besides *BDNF*, *PAX6*, and *C11orf46* may have independent effect on axonal and other anatomical phenotypes as well as behavioral outcomes in WAGR syndrome.

In summary, we identified a chromatin-associated mechanism underlying axonal development and show that C11orf46 plays an important role in transcallosal phenotypes in 11p13 deletion syndrome. Further investigation on epigenetic regulators in CNVs associated with neurodevelopmental disorders and altered neural connectivity is important for translational approaches that pave the way for novel treatment of neurodevelopmental psychiatric conditions in early life.

## Methods

### Human subjects

Patients who had prior genetic testing confirming diagnosis of WAGR/11p13 deletion syndrome or isolated aniridia with known *PAX6* mutation or deletion, as well healthy control subjects who had no chronic medical conditions were recruited through local advertisements and on-line postings. The study was approved by the institutional review board of the Eunice Kennedy Shriver National Institute of Child Health and Human Development, National Institutes of Health, Bethesda, MD and was registered at https://clinicaltrials.gov/ as NCT00758108. Written informed consent was obtained from adult subjects who were competent to provide consent and from the parents or legal guardians of children and adults with cognitive impairment. Clinical studies were performed between December 2008 and May 2014 at the NIH Clinical Research Center.

### Genetic testing

Deletion boundaries for each subject with WAGR syndrome were determined using oligonucleotide array comparative genomic hybridization using a custom-designed microarray platform (Agilent Technologies, Inc., Santa Clara, CA) containing 105,000 60-mer oligonucleotide probes using NCBI Build 36 (hg18) human reference sequence as previously described^63^. Probes for chromosome 11p were spaced at approximately 400 bp intervals (excluding repeat regions) using 57,925 probes; 121 probes were located within *BDNF*.

### Brain magnetic resonance imaging (MRI)

Brain MRI consisted of one cubic millimeter resolution, T1-weighted images collected on a 3.0 T Philips Achieva MRI scanner with an 8-channel phased array head coil. Corpus callosum volumes were calculated using FreeSurfer’s (version 5.3) subcortical image processing pipeline and published methods^64^. Briefly, the pipeline uses prior probability of a given tissue class at a specific atlas location, the likelihood of the image intensity given the tissue class, and the probability of the local spatial configuration of labels given the tissue class. The measured corpus callosum volume extends 2.5 mm from the midline on both sides to mitigate against any residual misalignment after registration to the template. Analysis of MRI data was performed blinded to genetic diagnosis of each patient. Each subject had a single structural MRI scan. MRI brain scans were normalized to a T1-weighted template (MNI305) using a 12-parameter affine transformation. No artifact or structured noise removal was applied to the MRI scans.

### Lymphoblastoid cell lines

Fasting venous blood samples were obtained from patients (Supplementary Table 3). Peripheral blood mononuclear cells were isolated using a Ficoll-Paque gradient (GE Heathcare Life Sciences, Pittsburgh, PA), then infected with Epstein Barr virus, and after sufficient cell line expansion, stored in freezing media at −80 °C.

### Antibodies

A mouse monoclonal antibody against Flag-tag (M2) (Sigma, Cat #F1804) was used for affinity purifications. For immunoblots, the following antibodies were used: rabbit monoclonal antibody against the C-terminus of human SETDB1 (Cell Signaling technology, Cat #2196S), KAP1 (cell signaling technology, Cat#4124S); rabbit polyclonal antibody against SETDB1 (Santa Cruz, Cat #sc-66884X), MCAF1 (Novus biological, Cat #NB100-438), Histone H3 C-terminus (Millipore, Cat #07-690). Custom-made mouse monoclonal antibody and guinea pig polyclonal antibody were generated against *E*. *coli* derived, purified and mass spectrometrically verified full-length human His-tagged C11orf46 protein (Supplementary Fig. 4b). For details on the generation of mouse monoclonal antibody, see the references^65,66^. Altogether, total 45 individually picked anti-C11orf46 mouse monoclonal hybridoma clones were screened by Western blot using *E*.*coli* derived human C11orf46 as antigen. Five clones (clone numbers 9, 26, 28, 33, and 36) were positive in this assay (clone 26 shown as representative example in Supplementary Fig. 4b). Antibodies from these clones were pooled and used for the co-immunoprecipitation assay using tissue homogenates of human prefrontal cortex. The guinea pig antibodies were generated by Cocalico Biologicals (Reamstown, PA) using affinity purified human recombinant His-tagged full-length C11orf46. Rabbit polyclonal antibodies against histone H3 lysine-9 monomethyl (Millipore, Cat #07-450), H3 lysine-9 dimethyl (Upstate, Cat #07-441), and H3 lysine-9 trimethyl (Upstate, Cat #07-442) were used for co-immunoprecipitating endogenous SETDB1 from HeLa nuclear extract. The antibodies which were used for immunohistochemistry or chromatin immunoprecipitation (ChIP) are described in each section.

### Plasmids

Plasmids expressing interfering shRNA were generated to suppress endogenous C11orf46 protein expression utilizing the pSUPERIOR.puro vector system (Oligoengine). Their target sequences are: C11orf46 shRNA-1 with strong suppression; 5′-CAAACTGAATTTGCTCCAGAA- 3′ and C11orf46 shRNA-2 with milder suppression; 5′- GAAGACAGCTTGTACCTGGTT- 3′. A scrambled sequence that shows no homology to any known messenger RNA was utilized to produce the Control shRNA (5′-ATCTCGCTTGGGCGAGAGTAAG-3′). The HA and scFv-tagged RNAi resistant wild-type human C11orf46 expression constructs containing four silent mutations (underlined) in the target sequence of C11orf46 shRNA (5′- CAAACAGAGTTCGCACCAGAA-3′) were produced and cloned into CAG promoter driven plasmid (pCAGGS1vector) for rescue experiments. dCas9-SunTag coding sequence was also transferred into pCAGGS1 vector. Two single guide RNA sequences each *Dclk1* or *Sema6a* promoter region were using online CRISPR design tool^67^. Those protospacer sequences were cloned into sgRNA cloning vector (Addgene #41824) with the protocol distributed on the addgene website (https//media.addgene.org/data/93/40/adf4a4fe-5e77-11e2-9c30-003048dd6500.pdf). The protospacer sequences are listed in Supplementary Table 5. CAG promoter driven mouse *Sema6a* expression plasmid (CAG-mSema6a) was cloned using Sema6a (NM_018744) Mouse Tagged ORF clone (ORIGENE, Cat#MR211486).

### In vitro epigenomic editing using dCas9-SunTag-C11orf46

For producing scFv-GCN4-sfGFP-C11orf46, full-length wild-type C11orf46 (C11orf46^wt^) or R236H mutant C11orf46 (C11orf46^R236H^) was cloned in-frame to the C-terminus of scFv GCN4 antibody using the BamH1 and NotI sites (addgene plasmid #60904). dCas9-SunTag (2 µg, addgene #60903) containing 10 copies GCN4 peptide epitope fused to catalytically inactive Cas9 was delivered into HEK293FT cells (1.5 × 10^6^, 60 mm dish) along with a 4 pooled sgRNAs (50 ng each sgRNA, U6-sgRNA-polyT amplicon) to direct GCN4 single-chain antibody conjugated fluorescent C11orf46 (2 µg, scFv-GCN4-sfGFP-C11orf46) to neurite-regulating gene promoters. Three days after transfection using lipofectamine 2000, total RNA was extracted and qPCR was performed. scFV-GCN4-sfGFP-VP64 was used as a positive control for activating gene expression. dCas9 alone was tested as a negative control to account background gene activity. For producing U6-sgRNA-polyT, mouse [Dec.2011 (GRCm38/mm10)] and human promoter sequences (500 bp) were identified using PromoterWise (http://www.ebi.ac.uk/Tools/psa/promoterwise/). Four sgRNAs spaced approximately 500 bp away from each other in the promoter region of the target genes were identified based on their high gRNA score and low/no off-target site in the genome (Supplementary Table 5). sgRNAs were incorporated into U6-sgRNA-polyT cassettes by PCR amplification using U6-Fwd and U6sgRNAter-Rev primers which carried the reverse complement of part of the U6 promoter. These cassettes include the sgRNA (+85) scaffold with guide sequence and seven T nucleotides for transcriptional termination.

### In utero electroporation (IUE)

IUE targeting the somatosensory cortex was performed using our previously published methods with minor modifications^20,21^. Pregnant C57/BL6 mice were anesthetized at embryonic day 15 (E15) by intraperitoneal administration of a mixed solution of Ketamine HCl (100 mg/kg), Xylazine HCl (7.5 mg/kg), and Buprenorphine HCl (0.05 mg/kg). After the uterine horn was exposed by laparotomy, the shRNA plasmid (1 µg/µl) together with CAG promoter-driven GFP expression plasmids (1 µg/µl) (molar ratio, approximately 1:1) were injected into the lateral ventricles with a glass micropipette made from a micropillary tube (Narishige, Cat #GD-1), and electroporated into the VZ of the CP at E15. C11orf46 expression constructs (1 µg/µl) were also co-introduced for rescue experiments. For in vivo epigenomic editing, CAG-dCas9-ST (1 µg/µl), scFv-C11orf46 (1 µg/µl) and sgRNAs (1 µg/µl) together with shRNAs and GFP expression plasmid will be introduced at E15. For overexpression of *Sema6a*, CAG-Sema6a (2 µg/µl) or tdTomato (1 µg/µl) with GFP expression plasmid will be introduced at E15. For electroporation, Electrode pulses (40 V; 50 ms) were charged four times at intervals of 950 ms with an electroporator (Nepagene, Cat #CUY21EDIT). All experiments were performed in accordance with the institutional guidelines for animal experiments of Johns Hopkins University.

### RNA-seq and bioinformatic analysis

Total RNA was isolated from four lymphoblastoid cell lines including one derived from healthy control subject, one WAGR patient with *C11orf46 gene* intact and two WAGR patients with *C11orf46* gene deletions using a total RNA extraction kit (Qiagen) according to manufacturer instructions. RNA integrity determined in Bioanalyzer (Agilent’s Technologies) and a total RNA-seq was performed at Eurofins genomics. DNA libraries were prepared from total RNA samples, the adaptor-ligated libraries (125 bp) were enriched by PCR amplification and gel purified (average library size of ~270 bp). Libraries were sequenced with the Genome Analyzer II (Illumina) using pair-end 50-bp raw fastq reads. The sequencing approach resulted in 32,750,145 and 27,929,697 and 39,292,408, and 54,377,092 reads for lymphoblastoid cell line sample numbers one, two, three, and four, respectively. Sequencing raw reads were assessed for quality control using FastQC (http://www.bioinformatics.babraham.ac.uk/projects/fastqc/). RNA-seq reads of 50 bp were aligned against the human genome (UCSC hg19) references with TopHat2 using default parameters with Bowtie as the internal aligner and a segment mapping algorithm to discover splice junctions^68,69^. In all, 77–82% of all unique sequences correctly aligned to the reference genome. Reads were counted in exon regions using featureCounts tool (subread v1.5.2) producing a table that was normalized to rpkms (Supplementary Table 3). Heatmaps were produced scaling rpkm values by scale function (base v3.5.1) and colors were adjusted by quantile breaking to allow colors represent an equal proportion of the data.

Two different techniques were used in order to assemble the transcriptome: (i) reference-guided assembly of RNA-seq reads using reference annotations from Refseq^70^ and (ii) non-reference guided assembly (without the help of a reference gene model) to yield a ‘transcript reference-free’ characterization of RNA-seq reads. For the first approach, BioConductor packages DESeq (References) and EdgeR v2.4.6 in the R programming environment were used. HTSeq-count (http://www-huber.embl.de/users/anders/HTSeq/doc/index.html) was used with UCSC annotation to generate a set of per-gene read counts for each sample, using default parameters including ‘stranded’ set to ‘no’. The HTSeq-count-generated matrix count dataset was used as input. The quartile of genes with the lowest counts was excluded to increase the detection power at a given FDR. For the second approach, the Cufflinks-CuffDiff-CummeRbund pipeline was used^69^. Fragments per kilobase per millions of reads (FPKM) values were computed for each cell line sample. Genes that were significantly differentially expressed with a FDR of 0.05 in DESeq and EdgeR were selected for further analysis. Both methods yielded highly reproducible results in the expression measurements of the transcripts, and the majority of differentially expressed genes were also significant in an independent analysis using Cufflinks.

### Generation of tet-inducible cell lines

Human full-length *SETDB1* complementary DNA (cDNA), identical to SETDB1 transcript variant 1 (NM_001145415.1; 3876 bp, 1291 amino acids, predicted molecular weight 143 kDa,) was reverse-transcribed using primer Cjk-SA1. Total RNA was isolated from human brain derived U87-MG glioblastoma cells (ATCC, Cat #HTB-14) and was subsequently amplified by PCR (Cjk-SA2 & Cjk-SA4). SalI-Not1 site was double digested and cloned into pENTR4 and transferred to the pFRT TO DESTFLAGHA vector by a LR reaction. The plasmid was completely sequenced. The FLAGHA epitope contributed 17 amino acids to the N-terminal of SETDB1. These amino acids fused at the N-terminus to the ~60 bp (1xFLAG)- (1xHA) epitope (FLAG-HA-SETDB1) downstream of a promoter containing the tetracycline-responsive element (pFRT-TO-FLAGHA-SETDB1) and stably transfected (hygromycin) into Flp-In^TM^ T-Rex 293^TM^ cells (ThermoFisher, Cat #R780-07) together with plasmid pOG44 (expressing FLP recombinase). Full-length *C11orf46* cDNA (NM_152316.2; 270 amino acids; 783 bp) was amplified by PCR from an Incyte plasmid (Openbiosystem, Cat #IHS1380-97432875) using primers Cjk24 and Cjk25, cloned into SalI-NotI restriction sites of the pENTR4 vector. A similar procedure was used to generate HEK293 cells for tet-inducible expression of a FLAG-HA-tagged full length *C11orf46* cDNA (NM_152316.1 784 bp, 260 amino acids). For cloning, high fidelity reverse transcriptase (AccuScript Hi-Fi; Agilent Technologies #600180) and DNA polymerase were used (Pfu Ultra DNA polymerase; Agilent Technologies #600380). All plasmids were sequence verified and the primers used in their generation are listed in Supplementary Table 6.

### Affinity purification of protein complexes

Inducible (Tet-On) HEK293 cell lines expressing SETDB1 or C11orf46 or control cells (10^7^ cells/assay) were treated with 2 μg/ml of doxycycline for 48 h before harvest. Pelleted cells were suspended in CS100-0.02 buffer (Tris HCl, pH 7.4, 10 mM MgCl_2_, 2.5 mM NaCl, 100 mM 0.02% NP-40, 2 units/ml Benzonase, protease inhibitor, and phosphatase inhibitor) at four times, packed cell volume, sonicated, passed five times through a 26-gauge needle, centrifuged at 20,000 × *g* for 15 min, and filtered through a 0.2-μm filter (Millipore, Cat #SCGP00525). Benzonase was added to eliminate nucleic acid-dependent indirect interactions. Lysate was passed through an anti-FLAG agarose column (packed using 0.5 ml of 50% slurry per 100 mg of extracts) at 4 °C and washed four times with cold CS100-0.02, then four times with cold CS500-0.02 (Tris HCl, pH 7.4, 10 mM MgCl_2_, 2.5 mM NaCl, 500 mM 0.02% NP-40, protease and phosphatase inhibitors). Column-bound protein complexes were eluted using 10 bed volumes of the CS100-0.02-containing 3X-FLAG peptide (100 ng/µl) at 4 °C. Purity of protein complexes was verified with four to fifteen percent gradient gel/silver stain and analyzed using mass spectrometry at the proteomics core facility in University of Massachusetts.

### In situ hybridization

The following Cyanine dyes-conjugated RNA probes were designed by assistance of Primer-BLAST (https://www.ncbi.nlm.nih.gov/tools/primer-blast/), C11orf46 antisense RNA probe: 5′-Cy3-AGCUUUAGCAGUCCUUCCAUCUGA-3′ and Sema6a antisense RNA probe: 5′-Cy5- GGAACUGGCGUGCCCAUUCAGUGC-3′ for each target gene, and NS (non-targeting) RNA probe-1: 5′-Cy3-AUCUCGCUUGGGCGAGAGUAAG-3′ to determine background signal. All probes were synthesized by Integrated DNA Technologies, Inc. Mouse brains were extracted after perfusion with 4% paraformaldehyde (PFA). The fixed brains were embedded in cryocompound (Sakura) after replacement of PFA with 30% sucrose in PBS. Coronal sections were obtained at 20 µm with a cryostat (Leica, Cat #CM 3050S). The sections were washed with DEPC-treated PBS (DEPC-PBS), followed by permealization by ice-cold methanol for 30 min. Hybridization buffer (50% Formamide, 10% Dextran sulfate, 0.1 mg/mL Yeast tRNA, 2× SSC, 50 mM Sodium phosphate) including 15 ng/µl of RNA probes were heat-denatured at 95 °C for 5 min and 1/500 vol. RNase inhibitor (20U/uL) was added to the buffer. After washing by DEPC-PBC, the permealized brain slices were hybridized with RNA-probes in hybridization buffer over night at 37 °C. The samples were then washed once with wash buffer (40% Formamide, 1x SSC) for 30 min at 37 °C. Final three-time washing steps by DEPC-PBS included nuclei labeling with DAPI (Roche, 10236276001). Fluorescent images were collected by a confocal microscope (Zeiss, LSM 700).

### Immunohistochemistry

Immunohistochemistry was performed using our previously published methods with some modifications^20,21^. Mouse brains were extracted after perfusion with 4% PFA. The fixed brains were embedded in cryocompound (Sakura) after replacement of PFA with 30% sucrose in phosphate buffered saline (PBS). Coronal sections including somatosensory cortex were obtained at 40 µm with a cryostat (Leica, Cat #CM 3050S). The sections were washed with PBS containing 0.5% Triton X-100 and then blocked with 0.5% Triton X-100 and 1% bovine skin gelatin for one hour. After blocking, sections were incubated with various cell markers [mouse anti-Nestin antibody (BD Biosciences, Cat #556309), rabbit anti-Pax6 antibody (BioLegend, Cat #901301), rabbit anti-TBR2 antibody (abcam, Cat #ab23345), rabbit anti-NeuroD2 antibody (abcam Cat #ab109406), mouse anti-SATB2 antibody (Santa Cruz Cat #sc-81376), rabbit anti-CUX1 antibody (Santa Cruz Cat #sc-13024), or rat anti-CTIP2 antibody (abcam, Cat #ab18465)] and guinea-pig polyclonal anti-C11orf46 primary antibodies at 4 °C overnight, followed by incubation with secondary antibodies conjugated to Alexa 488, 568 or 647 (Invitrogen, Cat #A11001(mouse), #A11006(rat), #A11008(rabbit) for Alexa 488, A11075(guinea-pig) for Alexa568, A21235 (mouse), A21244 (rabbit) for Alexa 647, respectively) for 1 h. Nuclei were labeled with DAPI (Roche, 10236276001). For cell type marker staining, anti-CaMKII (Millipore, Cat #05-532), anti-Olig-2 (Millipore, Cat #AB9610), anti-ALDH1L1 (abcam, ab177463), or anti-Iba-1 (Wako, Cat #019-19741) antibody were used. For analysis of neuronal migration and axonal outgrowth, coronal sections from fixed brains were obtained at 20 and 100 µm, respectively. The sections were washed with PBS containing 0.5% Triton X-100 and then blocked with 0.5% Triton X-100 and 5% normal goat serum for one hour. After blocking, sections were incubated with rat monoclonal anti-GFP (Nacalai, Cat #GF90R) antibody at 4 °C overnight, followed by incubation with secondary antibodies conjugated to Alexa 488 for 1 h.

### Fluorescence-activated cell sorting (FACS)

The mice subjected to IUE at E15 underwent whole brain extraction at P0 or P14. The somatosensory cortex where GFP-positive neurons were localized was dissected using a stereomicroscope with a fluorescent flashlight (Night Sea, Cat #DFP-1). Cells were separated by utilizing papain dissociation kit (Worthington, Cat #LK003150) with minor modification^71^. For ChIP-qPCR experiments, approximate 0.5–1.0 × 10^6^ GFP-positive neurons obtained by pooling 5–6 pups’ GFP-positive cortices were collected by FACS into 1.5 ml Protein LoBind Tube (Eppendorf, Cat #Z666505).

### Quantitative real time PCR (qPCR)

FAC-sorted GFP positive somatosensory cortical neurons were suspended into RNAlater solution (ambion Cat#AM7021) and stored at −80 °C. Total RNA were prepared and treated by DNAse (PicoPure™ RNA Isolation Kit, Applied Biosystems Cat#KIT0204). Total RNA was reverse transcribed to cDNA (SuperScript™ III First-Strand Synthesis System, Invitrogen Cat#18080051). qPCR was performed using PowerUp™ SYBR™ Green Master Mix (Applied Biosystems Cat#A25742). *Hprt* was used as a reference gene to normalize the expression data by ddCt method. Target gene primers (*Sema6a*, *Dclk1*, *Gap43*, and *Hprt*) are listed in Supplementary Table 6.

### Chromatin immunoprecipitation (ChIP)

FAC-sorted GFP-positive cells were cross-linked with 1% formaldehyde for 15 min at room temperature, quenched with glycine to a final concentration of 0.125 M for another 10 min, and stored at −80 °C overnight before processing. After cell lysates with the lysis buffer (50 mM Tris-HCl, 10 mM EDTA (pH8.0), 1% SDS, and protease inhibitor cocktail (Roche)), chromatin was sonicated with a Q Sonica Sonicator (45% amplitude, 15 s 20 times with 1 min interval), cleared by centrifugation, and incubated overnight at 4 °C with 5–7 μg of the desired anti-H3K9me3 (abcam, Cat #ab8898). Five micrograms of chromatin was used for each immunoprecipitate. Immunocomplexes were immobilized with 30 μl of protein-G agarose beads (Active Motif) for 4 h at 4 °C, followed by stringent washes (wash buffers will be described) and elution. Eluates were reverse cross-linked overnight at 65 °C and deproteinated with proteinase K at 56 °C for 1 h. DNA was extracted with phenol chloroform, followed by ethanol precipitation. ChIP-qPCR analyses were performed in a QuantStudio (Applied Biosystems, 12K Flex) using PowerUp™ SYBR™ Green Master Mix (Applied Biosystems, Cat#A25742. ChIP-qPCR signals were calculated as a percentage of input. Fold induction was calculated over a negative genomic region. All primers used in qPCR analyses are shown in Supplementary Table 6.

### Quantification of axonal phenotypes

Axonal arborization was assessed by previously published methods with some modifications^6,72^. Tiled scanned images of the somatosensory cortex where GFP-positive transcallosal neurons projected from the IUE-targeted contralateral hemisphere were collected by a confocal microscope (Zeiss, LSM 700). Images were taken by the 10x objective lens (0.8× zoom-out), with 1024 × 1024 pixel resolution at a scan speed of seven per section. Single plain images at corpus callosum were taken by the 10× objective lens. Acquisition parameters were kept constant for all scans. Contralateral arborization signals were analyzed in images of whole cortex which were cropped with 9 inches width, and GFP signal from layer I to layer V was quantified by 20 equal spaces (20 bins) by ImageJ software. GFP signal intensity at corpus callosum was measured by ImageJ. Contralateral and callosal signal intensity were normalized by GFP-positive cell density in ipsilateral (electroporated) site. Three to six mice were used for quantification for each group.

### Quantitative bin analysis of brain slices

The effect of knockdown of C11orf46 on neuronal migration was assessed by performing quantitative bin analysis by our previously published methods^19–21^. The number of GFP-positive cells in the developing somatosensory cortex, including the CP, intermediate zone (IZ), and SVZ/VZ, were counted. The somatosensory cortex was divided into 10 equal spaces (10 bins) and the percentage of GFP-positive cells in each bin was determined. The numbers of neurons in each category from more than three independent experiments were counted in a blinded manner using ImageJ software (http://rsb.info.nih.gov/ij/).

### Statistical analyses

For analysis of human MRI data, IBM SPSS Statistics (version 24) software was used. Kolmogorov-Smirnov testing was performed to confirm normal distribution of patient data. Univariate fixed effect ANCOVAs accounting for age and sex as covariates compared white matter structure volumes among patient groups, and post hoc pairwise two-sided Least Significant Difference (LSD) comparisons were performed. Bonferroni correction was applied due to comparison of multiple corpus callosum regions among patient groups; nominal *p*-values are shown and threshold of *P* < 0.008 (=0.05/6) was considered significant. One-way ANOVA with the Bonferroni multiple comparison test was used for factorial analysis among more than three groups. Student’s *t*-test was used for comparisons between two groups. All data are shown as mean ± SEM, unless otherwise mentioned.

### Reporting summary

Further information on research design is available in the Nature Research Reporting Summary linked to this article.

## Supplementary information

Supplementary Information

Peer Review

Reporting Summary

## Data Availability

The raw RNA-seq data that support the findings of this study have been deposited to GEO under the accession number GSE119360. All data are available from the corresponding authors, A.K. and S.A. upon reasonable request. Human subject data will be deidentified to protect confidentiality.
