# Supplementary material for: In vivo epigenetic editing of Sema6a promoter reverses transcallosal dysconnectivity caused by C11orf46/Arl14ep risk gene
Source: Nat Commun. 2019 Sep 11;10:4112. doi: 10.1038/s41467-019-12013-y (PMC6739341; doi:10.1038/s41467-019-12013-y)
Supplement: Supplementary file 1 — Supplementary Information [file 41467_2019_12013_MOESM1_ESM.pdf]

Supplementary Information

In vivo epigenetic editing of *Sema6a* promoter reverses transcallosal dysconnectivity caused by *C11orf46/Arl14ep* risk gene

Cyril J. Peter[1,*], Atsushi Saito[2,*], Yuto Hasegawa[2], Yuya Tanaka[2], Mohika Nagpal[2], Gabriel Perez[2], Emily Alway[2], Sergio Espeso-Gil[1], Tariq Fayyad[1], Chana Ratner[1], Aslihan Dincer[1], Achla Gupta[1,8], Lakshmi Devi[1,8], John G. Pappas[3], François M. Lalonde[4], John A. Butman[5], Joan C. Han[6,7], Schahram Akbarian[1,#], and Atsushi Kamiya[2,#]

[1]Friedman Brain Institute and Department of Psychiatry, Icahn School of Medicine at Mount Sinai, New York, NY 10029, USA

[2]Department of Psychiatry and Behavioral Sciences, Johns Hopkins University School of Medicine, Baltimore, MD 21287, USA

[3]Department of Pediatrics, New York University School of Medicine, New York, NY

[4]Human Genetics Branch, National Institute of Mental Health, Bethesda, MD 20892, USA.

[5]Diagnostic Radiology Department, The Clinical Center of the National Institutes of Health, Bethesda, MD 20892, USA

[6]Unit on Metabolism and Neuroendocrinology, Eunice Kennedy Shriver National Institute of Child Health and Human Development, National Institute of Health, Bethesda, MD 20892, USA

[7]Departments of Pediatrics and Physiology, University of Tennessee Health Science Center, and Children's Foundation Research Institute, Le Bonheur Children's Hospital, Memphis, TN, 38103, USA

[8]Department of Pharmacology and System Therapeutics, Icahn School of Medicine at Mount Sinai, New York, NY 10029, USA

[*]These authors contributed equally to this work.

[#]Correspondence: akamiya1@jhmi.edu (A.K.), schahram.akbarian@mssm.edu (S.A.).

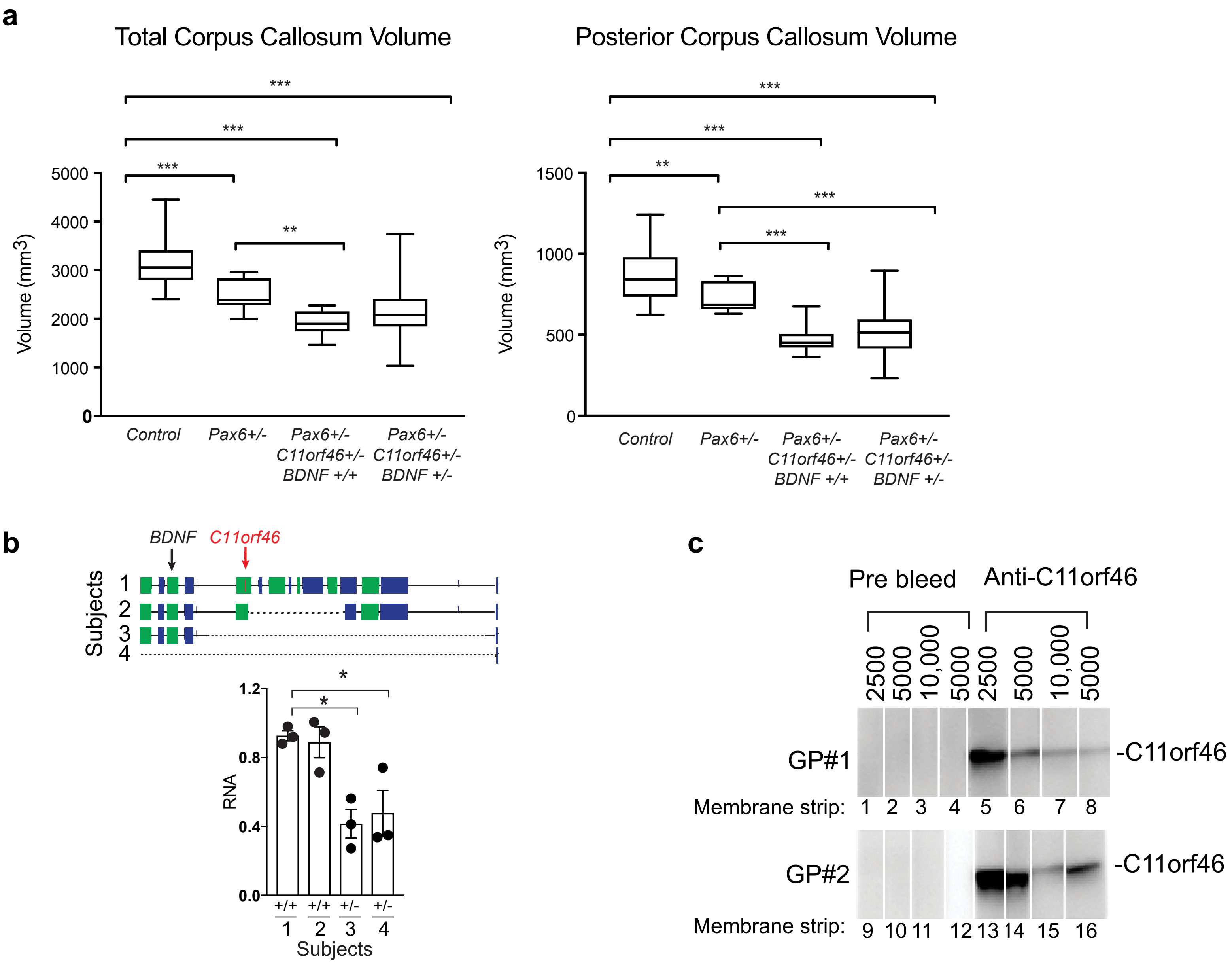
a
Total Corpus Callosum Volume
Posterior Corpus Callosum Volume
Volume (mm3)
***
**
Control
Pax6+/-
Pax6+/- C11orf46+/- BDNF +/+
Pax6+/- C11orf46+/- BDNF +/-
b
BDNF
C11orf46
Subjects
RNA
+/+
+/-
c
Pre bleed
Anti-C11orf46
2500
5000
10,000
GP#1
GP#2
-C11orf46
Membrane strip:

Supplementary Fig. 1 The critical role of C11orf46 for interhemispheric connectivity and knockdown effect of C11orf46. a Total (left) or posterior (right) CC volumes in participants with BDNF and without BDNF. ANCOVA including age and sex as covariates, compared corpus callosum volumes of healthy control (n = 23), isolated *PAX6+/-* (n = 12), *PAX6+/- C11orf46+/-* without *BDNF* haploinsufficiency (n = 7) and *PAX6+/-* and *C11orf46+/-* with *BDNF* haploinsufficiency (n = 10). No significant differences in the CC volumes of *PAX6+/-C11orf46*+/- patients with and without *BDNF+/-* (*P* = 0.19 and 0.47, for unadjusted comparisons; *P* = 0.13 and 0.37 respectively on ANCOVA adjusting for age and sex). Box and whisker plots represent the distribution of corpus callosum volume from each participant. The inner bar in the box indicates average value. The upper and lower box ends represent first and third quantile, respectively. The upper and lower whisker ends represent the maximum and minimum values in the group, respectively. **b** Schematic illustration of genetic map highlighting various types of micro deletions at 11p13 WAGR locus (dotted lines) including *C11orf46*; one control and three different types of microdeletion in WAGR patients are indicated. mRNA levels of C11orf46 in WAGR patients lymphoblastoid cells were quantified. Bar graphs represent the averages of mRNA expression in three independent experiments per patient. n = 3, 1 (*C11orf46* +/+) versus 3 (*C11orf46* +/-) *P* = 0.0139, 1 (*C11orf46* +/+) versus 4 (*C11orf46* +/-) *P* = 0.0239. Error bars indicate S.E.M. **c** Immunoblot with affinity purified guinea pig (GP) anti-C11orf46 antibodies detecting full-length human C11orf46. Note the pre-immune IgG from same animals (GP#1 & GP#2) did not detect C11orf46 (negative control). Antibody dilutions 1:5000 were used.

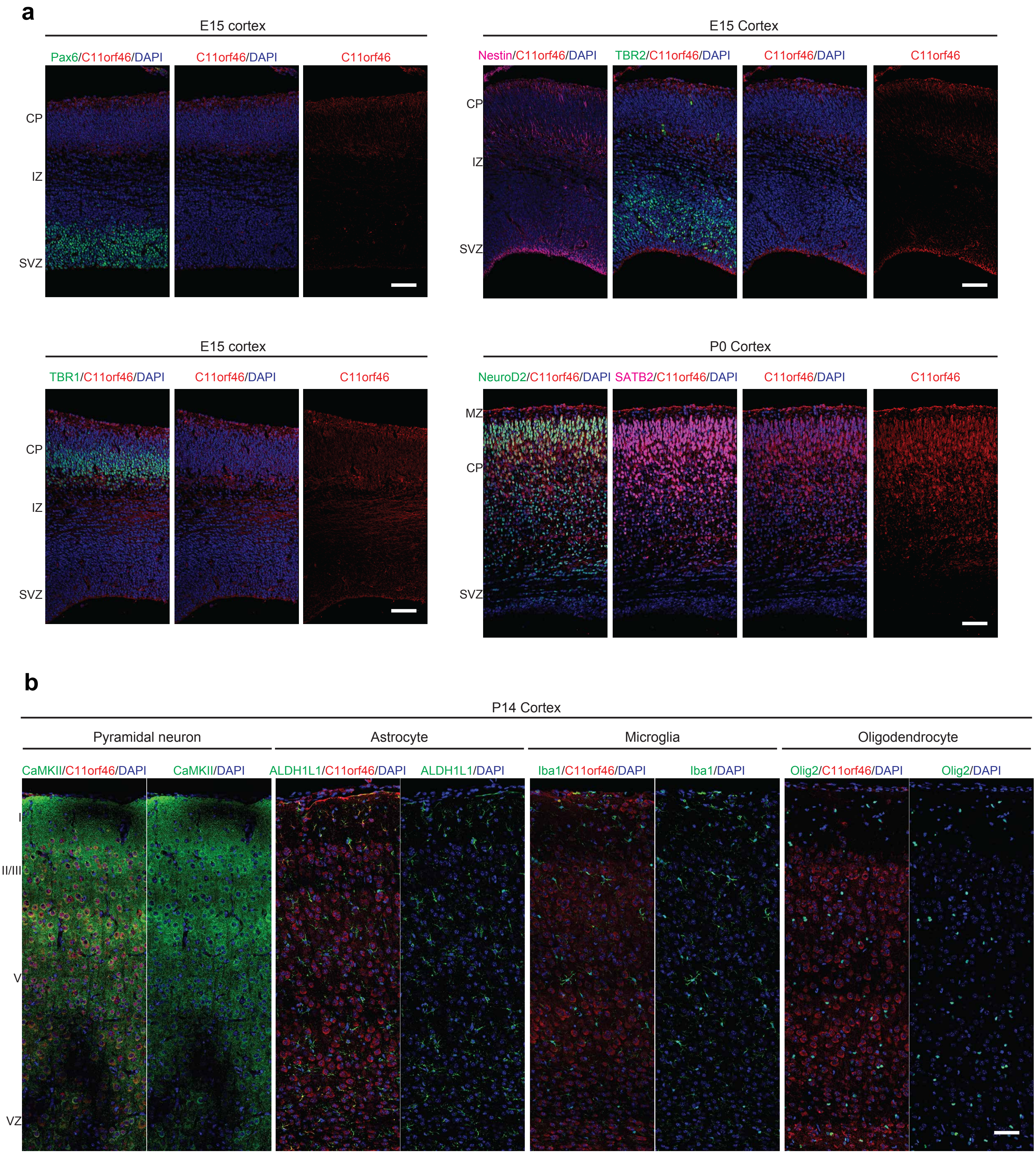

Cell-type	Pyramidal neuron	Astrocyte	Microglia	Oligodendrocyte
C11orf46(+) cells/Cell-type marker(+) cells	339/393	42/93	2/42	9/58
Ratio	86.3%	42.2%	4.8%	15.5%

Supplementary Fig. 2 C11orf46 is highly expressed in the neuronal cells in cortical plate and pyramidal neuron after differentiation. a (Upper left) Low magnification images of C11orf46 (red) protein staining with Pax6 (green) in the cerebral cortex at E15. (Upper right) Low magnification images of C11orf46 (red) protein staining with Nestin (magenta) and TBR2 (green) in the cerebral cortex at E15. (Lower left) Low magnification images of C11orf46 (red) protein staining with TBR1(green) in the cerebral cortex at E15. (Lower right) Low magnification images of C11orf46 (red) protein staining with NeuroD2 (green) and SATB2 (magenta) in the cerebral cortex at P0. Nuclear and cytoplasmic distribution of C11orf46 are shown in higher magnification images in **Fig. 1f**. Blue, nucleus counterstained by DAPI. Scale bar, 100 µm. **b** Low magnification images of C11orf46 protein staining (red) with cell type-specific markers (green) in the cortex. C11orf46 is predominantly expressed in CaMKII-positive pyramidal neurons, but not in ALDH1L1 (astrocyte), Iba-1 (microglia), or Olig2 (oligodendrocyte)-positive cells in the cerebral cortex at P14. (Bottom) Ratio of the number of nuclear C11orf46-positive cells in each specific marker-positive cells are shown in the table. Higher magnification images are shown in **Fig. 1g.** Scale bar, 100 µm.

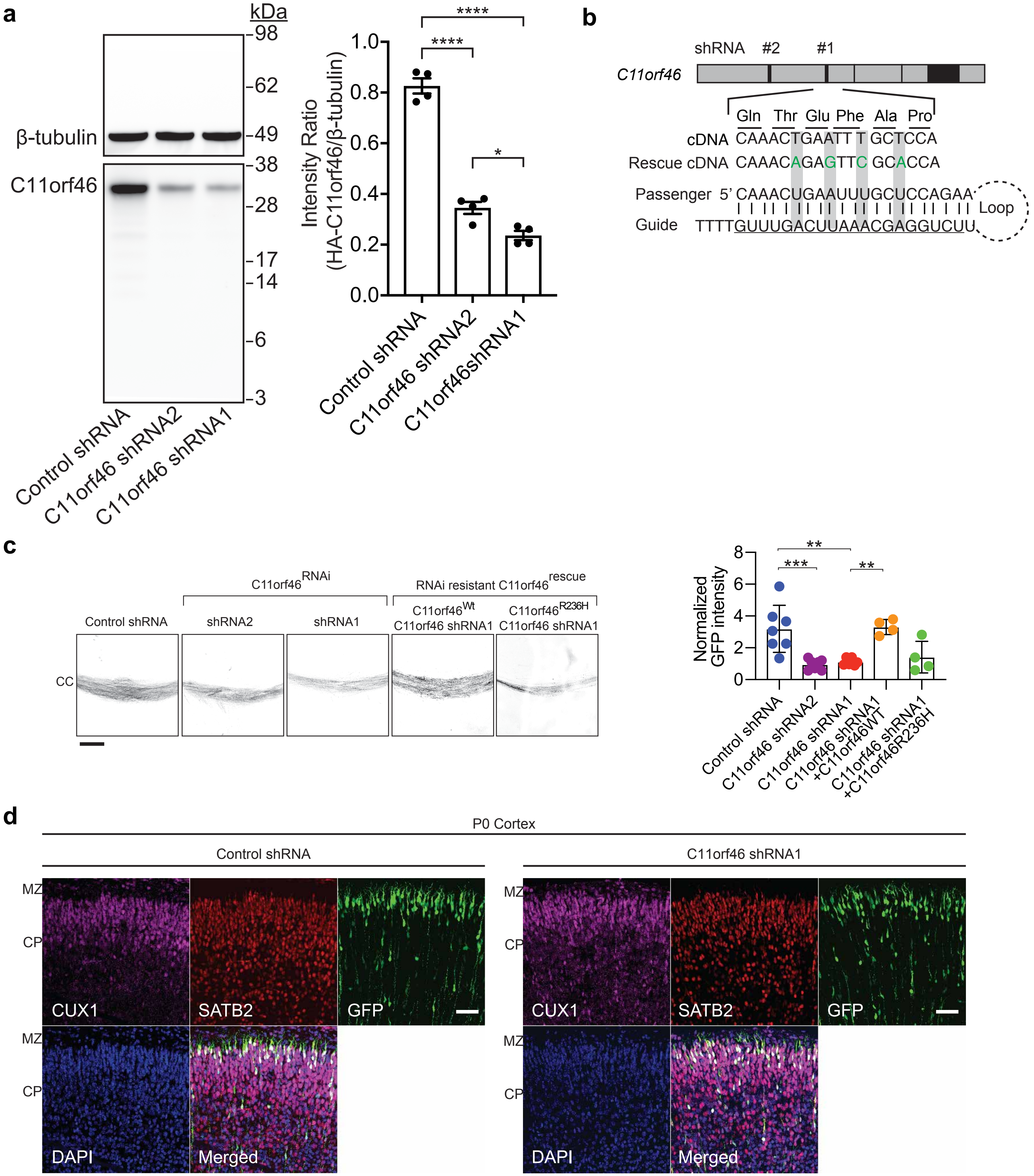

	Control shRNA	C11orf46 shRNA1
# of GFP(+) cells	132	140
# of GFP(+),SATB2 (+) cells (Ratio)	129 (97.7%)	137 (97.8%)
# of GFP(+),CUX1(+) cells (Ratio)	130 (98.5%)	138 (98.6%)

Supplementary Fig. 3. Knockdown plasmids against *C11orf46* and its effects on midline crossing of callosal projections and neuronal marker expression. a (Left) Western blot analysis of whole cell extract derived from HEK293 cells cotransfected with HA-C11orf46 and shRNA (C11orf46 or Control) plasmids blotted with anti-HA antibody. Endogenous β-tubulin was used as a loading control. (Right) Exogenous C11orf46 protein expression was strongly suppressed by shRNA1, compared with shRNA2. F (2, 9) = 166.4, P < 0.0001 was determined by one-way ANOVA with post hoc Bonferroni test. **b** Nucleotide sequence targeted by C11orf46 shRNA1. Four synonymous mutations were added within shRNA1 (bottom) target sequence in the rescue plasmids (green). **c** Knockdown of C11orf46 show impaired midline crossing of callosal projections at P14. Normalized GFP intensity at corpus callosum was decreased by delivery of C11orf46 shRNA1 and shRNA2 when compared with control shRNA (first 3 panels). These deficits were partially rescued by co-expressing RNAi resistant wild type C11orf46 ($C11orf46^{Wt}$), but not by R236H mutant ($C11orf46^{R236H}$) (last 2 panels). (Right) Quantitative data are shown in the graph. F (4, 23) = 9.9, P < 0.0001 was determined by one-way ANOVA with *post hoc* Bonferroni test. Scale bar, 100 µm. **d** CUX1 (magenta) and SATB2 (red) expression in the P0 brains after C11orf46 knockdown. Knockdown of C11orf46 does not affect the percentage of SATB2 and CUX1-positive cells in GFP-labeled neurons, compared to those in controls. Scale bar, 50 µm. *P < 0.05, **P < 0.01, ***P < 0.001, and ****P < 0.0001. Bar graphs indicate mean ± S.E.M (a,c).

a

```
Human   MMDPCSVGVQLRTTNECHKTYYTRHTGFKTLQELSSNDMLLLQLRTGMTL 50
Mouse   -MDPCSVGVQLRTTHDCHKTFYTRHTGFKTLKELSSNDMLLLQLRTGMTL 49
Chicken -MDPCSVGVQLQATNECHKTYYTRHTGFKTKEDISSFDLLLLQLRTGMTL 49
         *********::*::****:********* :::** *:***********

Human   SGNNTICFHHVKIYIDRFEDLQKSCCDPFNIHKKLAKKNLHVIDLDDATF 100
Mouse   SGNNTICLHHVKIYIDRFEDLQKSCCDPFNIHKKLAKKNLHVIDLDDATF 99
Chicken SENDTICFHHAKIYIERFEDLQKSCCDPFNMHRKLSKKNLRAIDLHDATF 99
        * *:***:**.*:**:***:**********:*:**:****:.***.****

Human   LSAKFGRQLVPGWKLCPKCTQIINGSVDVDTEDRQKRKPESDGRTAKALR 150
Mouse   LSAKFGRQLVPGWKLCPKCTQIINGSVDVDSDDRQRRKPDSDGRTAKALR 149
Chicken LTAKFGRQFVPGWKLCPKCMQVINGSVDVEAEERQRRKLDSDGRTAKALK 149
        *:******:********* *:*******:::**:** :*********:

Human   SLQFTNPGRQTEFAPETGKREKRRL-TKNATAGSDRQVIPAKSKVYDSQG 199
Mouse   SLQFTNPGKQTEFAPEGGKREKRKL-TKATSAASDRQIIPAKSKVYDSQG 198
Chicken SLQFTNPGRQTEFTPETSKREKRRLQTKNPSFNSDRQVIPAKSKVYDSQG 199
        ****:***:****:** .*****:* ** .:  ****:************

Human   LLIFSGMDLCDCLDEDCLGCFYACPACGSTK GAECRCDRKWLYEQIEIE 249
Mouse   LLIFSGMDLCDCLDEDCLGCFYACPTCGSTKCGAECRCDRKWLYEQIEIE 248
Chicken LLLYSGMDLCDCLDEDCLGCFYACPKCGSNKCGTECRCDRKWLYEQIEIE 249
        **::********************* ***.***:****************

Human   GGEIIHNKHAG----------------- 260
Mouse   GGEIIHNKHAGKAYGLLSPCHPYDILQK 276
Chicken GGEIIRNKHVG----------------- 276
         **** :***.*
```

b

#	C11orf46 Peptides Identified by mass spectrometry	Clone-8 (C11orf46 ▲)	
1	MMDPCSVGVQLRT	+	+
2	KTLQELSSNDmLLLQLRT	+	+
3	RTGMTLSGNNTICFHHVKI	+	+
4	KIYIDRFEDLQKS	+	+
5	KSCCDPFNIHKKL	+	+
6	KKNLHVIDLDDATFLSAKF	+	+
7	TQIINGSVDVDTEDRQK	+	+
8	RSLQFTNPGRQ	+	+
9	RQTEFAPETGKRE	+	+
10	KWLYEQIEIEGGEIIHNKH	-	+

c

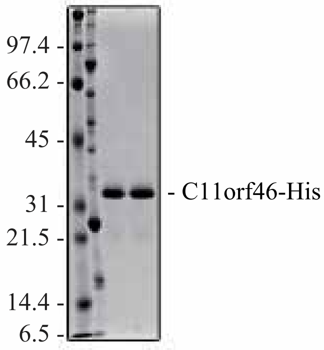

d

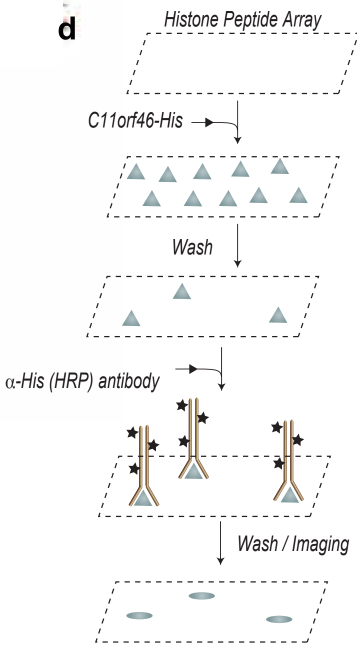

e

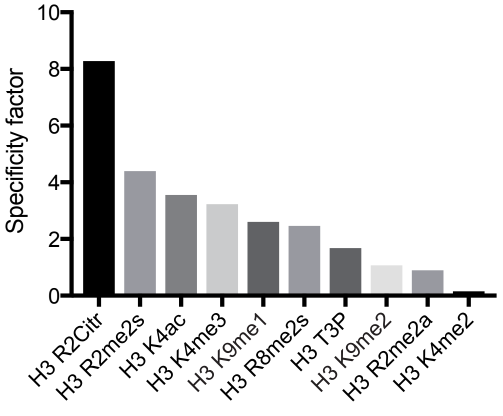

Top 10 (out of 384) modified histone peptides
in histone array bound by C11orf46-His

f

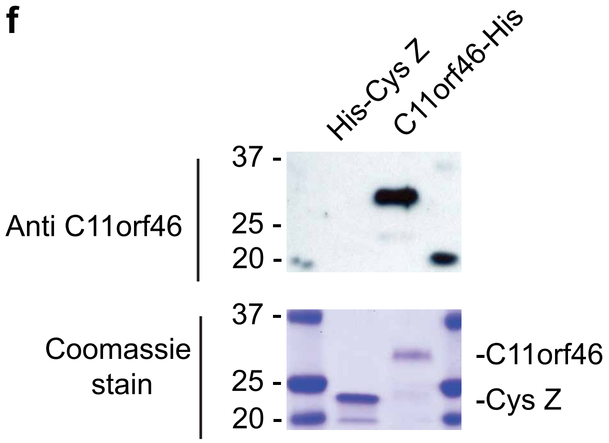

Supplementary Fig. 4 C11orf46 is a chromatin regulator. a, b C11orf46 is a conserved molecule. CLUSTAL W multiple sequence alignment of human C11orf46 protein and mouse and chicken *orthologs*. Identical (*), similar (:) critical point mutation associated with cognitive diseases (see text) marked in red. C11orf46 peptides present in C11orf46 complexes purified from clones 8 & 10 are highlighted (bold and underlined) (**a**) and detailed in the table (**b**). Note absence of carboxyl terminal peptide (marked in green) in clone 8 expressing truncated C11orf46. **c** Immunoblot showing recombinant protein load (250ng each). **d** Purified C11orf46-His incubated with Histone peptide array followed by HRP labeled anti-Histidine antibody detection and imaging analysis. **e** Histone peptide array showing purified C11orf46 binding to histone H3 N-terminal tail including its modifications. Top ten histone H3 peptides (out of >300 on the array) detected by C11orf46 includes H3K9me2/3. **f** Immunoblot showing mouse anti-C11orf46 detecting *E. coli* derived histidine-tagged full-length human C11orf46 but not the control protein CysZ; coomassie stained gel (bottom).

a

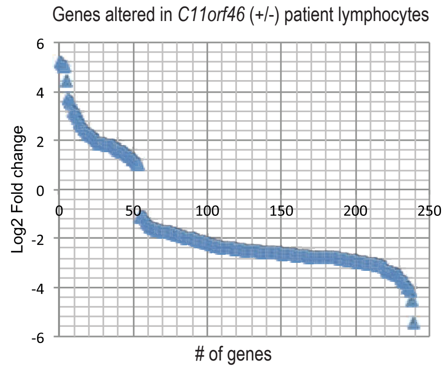
Genes altered in *C11orf46* (+/-) patient lymphocytes
Log2 Fold change
6
4
2
0
-2
-4
-6
0
50
100
150
200
250
# of genes

b

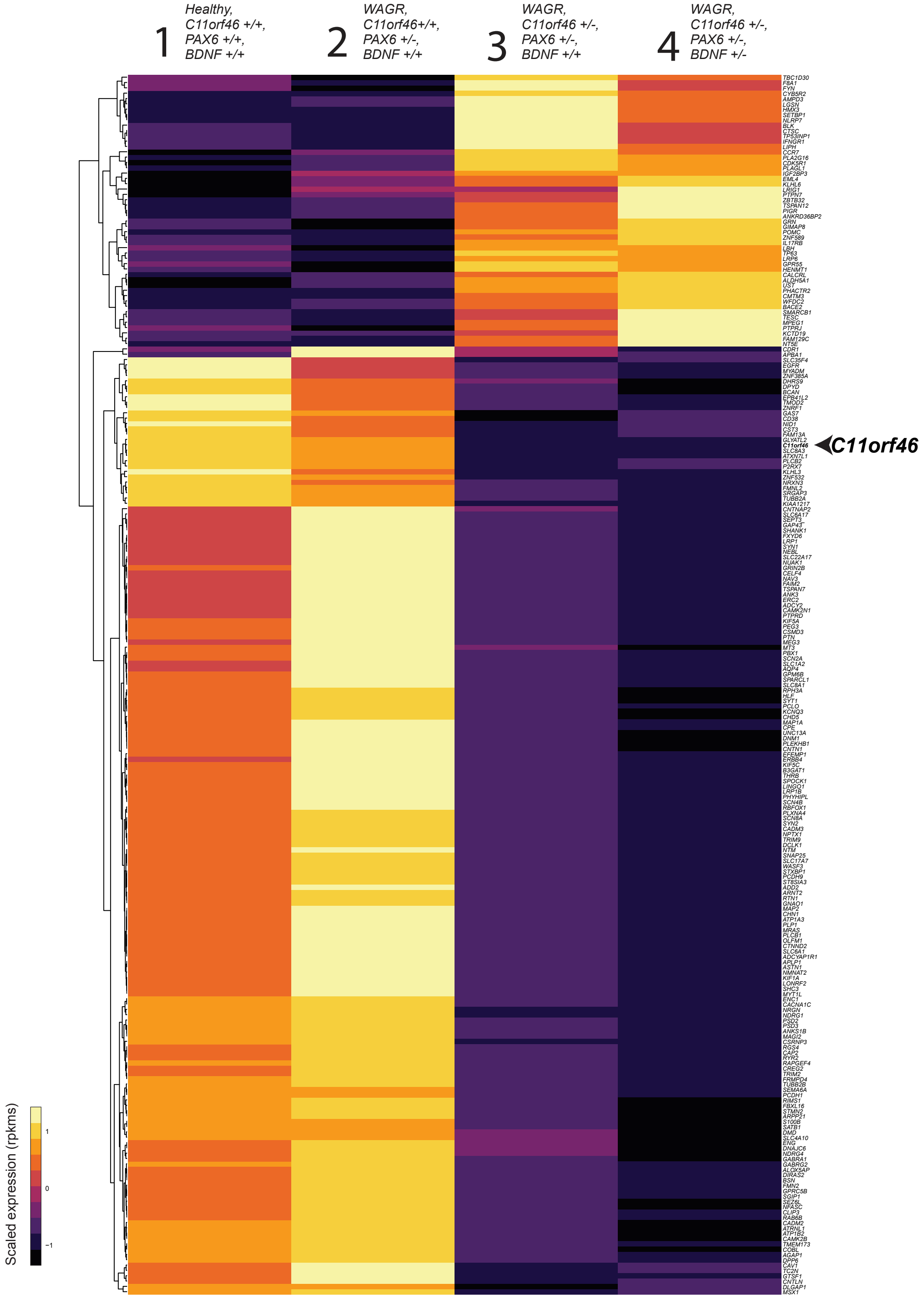
1
Healthy, C11orf46 +/+, PAX6 +/+, BDNF +/+
2
WAGR, C11orf46+/+, PAX6 +/-, BDNF +/+
3
WAGR, C11orf46 +/-, PAX6 +/-, BDNF +/+
4
WAGR, C11orf46 +/-, PAX6 +/-, BDNF +/-
C11orf46
Scaled expression (rpkms)
1
0
−1

Supplementary Fig. 5 RNA-seq dataset obtained from lymphoblastoid cell lines of the WAGR cases and controls. a Genes differentially expressed in *C11orf46* haploinsufficient WAGR lymphoblastoid cells. **b** Pair-end (50bp) RNA sequenced libraries were mapped to human (GRCh38.p10_v26) with STAR (v2.5.3a) using a two-method step protocol following tool specifications. Reads were counted in exon regions using featureCounts tool (subread v1.5.2) producing a table that was normalized to rpkms (**Supplementary Table 3**). Heatmaps were produced scaling rpkm values by scale function (base v3.5.1).

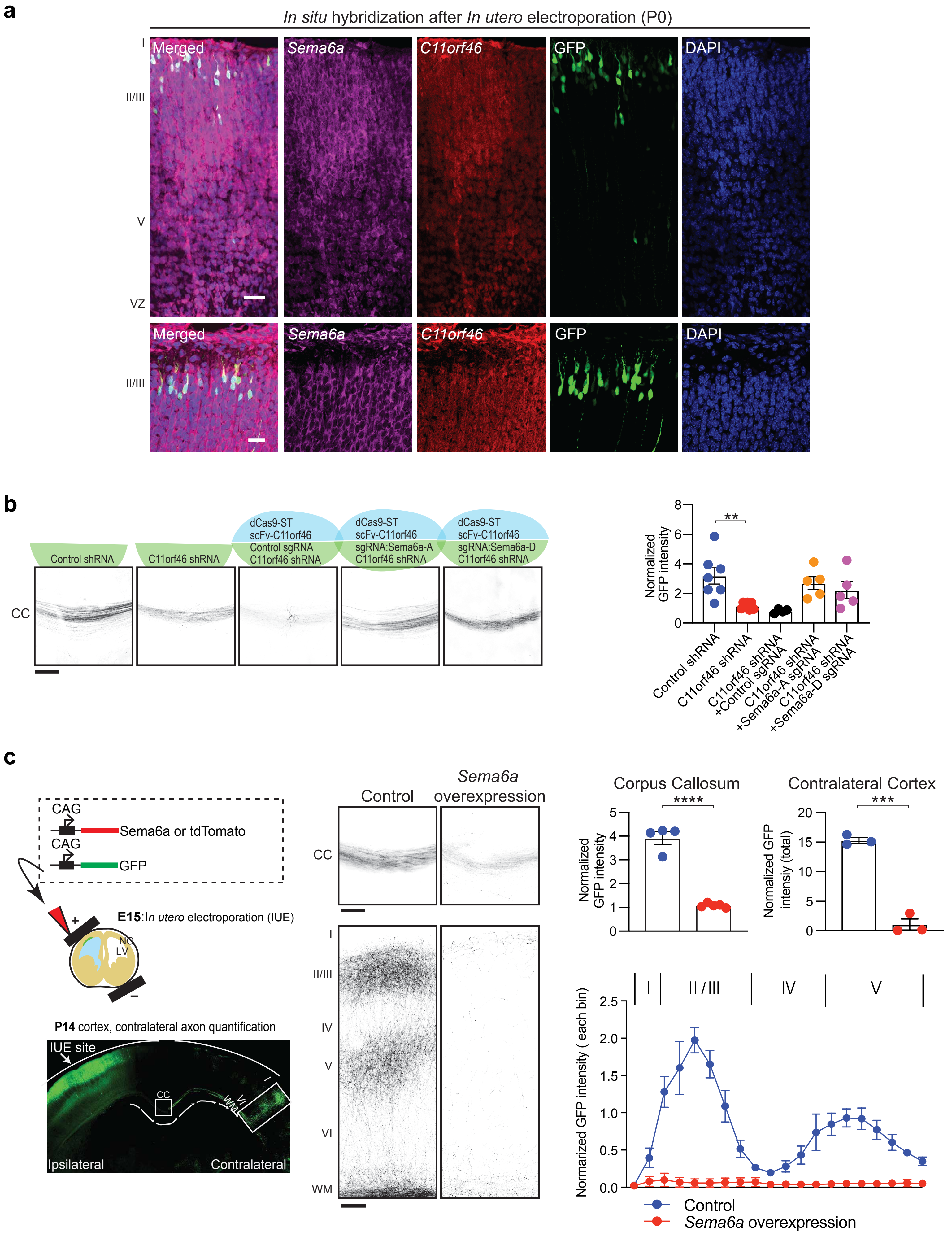

a
In situ hybridization after In utero electroporation (P0)
I
II/III
V
VZ
Merged
Sema6a
C11orf46
GFP
DAPI
b
Control shRNA
C11orf46 shRNA
dCas9-ST
scFv-C11orf46
Control sgRNA
C11orf46 shRNA
sgRNA:Sema6a-A
sgRNA:Sema6a-D
CC
Normalized GFP intensity
**
Control shRNA
C11orf46 shRNA
C11orf46 shRNA +Control sgRNA
C11orf46 shRNA +Sema6a-A sgRNA
C11orf46 shRNA +Sema6a-D sgRNA
c
CAG
Sema6a or tdTomato
GFP
E15:In utero electroporation (IUE)
NC
LV
P14 cortex, contralateral axon quantification
IUE site
Ipsilateral
Contralateral
Control
Sema6a overexpression
CC
IV
VI
WM
Corpus Callosum
Contralateral Cortex
****
***
Normalized GFP intensity
Normalized GFP intensiy (total)
I
II / III
IV
V
Normarized GFP intensity (each bin)
Control
Sema6a overexpression

Supplementary Fig. 6 Sema6a plays critical roles for axonal development. a *In situ* hybridization of *C11orf46* and *Sema6a* in the electoporated brains. *C11orf46* (red) and *Sema6a* (magenta) are highly expressed in the upper cortical layers II/III in the cerebral cortex at P0. Scale bar, 50 µm (upper panels) and 20 µm (lower panels). **b** The midline crossing of callosal projections was disrupted by *C11orf46* knockdown, but restored by dCas9-ST mediated recruitment of C11orf46 to two different part of *Sema6a* promoter (Sema6a-), using specific sgRNAs Sema6a-A, Sema6a-D, but not with non-targeting control sgRNA. Normalized GFP intensity at corpus callosum was shown in the graph. Control shRNA and C11orf46 shRNA samples were shared with Supplementary Fig. 3c. F (4, 23) = 5.633, P = 0.0026 was determined by one-way ANOVA with *post hoc* Bonferroni test. Scale bar, 100 µm. **c** (Left) Schematic diagram of *Sema6a* overexpression into transcallosal projection neurons by *in utero* electroporation. Sema6a or tdTomato overexpression plasmid together with GFP expression construct were delivered in the developing cerebral cortex at E15, followed by analysis of corpus callosum (CC) formation and axonal arborization at P14. (Middle) Overexpression of *Sema6a* in pyramidal neurons of layer II/III (Ipsilateral) impaired midline crossing of callosal projections and axon terminal arborization across all cortical layers in the contralateral somatosensory cortex at P14. (Upper right) Normalized GFP intensity at corpus callosum and total GFP intensity (axonal density) in contralateral cortex were shown in brains with overexpression of *Sema6a* and controls. $P < 0.0001$ (corpus callosum) and $P = 0.0002$ (contralateral cortex) were determined by Student's t-test. (Lower right) Layer distributions of callosal axons in the contralateral site of the electroporated brains were shown as normalized immunofluorescence intensities of callosal axons. Scale bar, 100 µm. **P < 0.01, ***P < 0.001, and ****P < 0.0001. Bar graphs indicate mean ± S.E.M (b,c).

Supplementary Table 1. The complete list of proteins identified by mass spectrometry analysis of purified Flag-C11orf46 complexes.

S.No	Protein ID	Description	Sequence Coverage (%)			Exclusive Unique Peptide Count			Total Spectrum Count		
			HEK293 (Wt; +Dox)	HEK293 (Tet-ON Flag-C11orf46; clone #10; +Dox)	HEK293 (Tet-ON Flag-C11orf46▲; clone #8; +Dox)	HEK293 (Wt;+Dox)	HEK293 (Tet-ON Flag-C11orf46; clone #10; +Dox)	HEK293 (Tet-ON Flag-C11orf46▲; clone #8; +Dox)	HEK293 (Wt; +Dox)	HEK293 (Tet-ON Flag-C11orf46; clone #10; +Dox)	HEK293 (Tet-ON Flag-C11orf46▲; clone #8; +Dox)
1	RS27A	Ubiquitin-40S ribosomal protein S27a (RPS27A)	8.30%	47%	31%	1	5	3	1	6	3
2	**C11orf46**	Uncharacterized protein C11orf46 (C11orf46)		45%	41%		10	9		20	19
3	**SETDB1**	Histone-lysine N-methyltransferase SETDB1 (SETDB1)		40%			42			102	0
4	H2A1D	Histone H2A type 1-D (HIST1H2AD)	15%	35%	28%	1	4	5	5	8	11
5	**MCAF1**	Activating transcription factor 7-interacting protein 1 (ATF7IP)		34%			26			63	0
6	IF6	Eukaryotic translation initiation factor 6 (EIF6)	5.70%	33%	43%	1	5	6	1	8	14
7	H12	Histone H1.2 (HIST1H1C)		26%	6.10%		4	1		4	1
8	RS11	40S ribosomal protein S11 (RPS11)	13%	25%	32%	2	4	5	2	4	5
9	PABP4	Polyadenylate-binding protein 4 (PABPC4)	7.80%	24%	20%	1	6	4	8	15	12
10	DDX21	Nucleolar RNA helicase 2 (DDX21)	5.00%	23%	26%	2	12	15	3	15	22
11	EIF3K	Eukaryotic translation initiation factor 3 subunit K (EIF3K)	6.40%	23%	13%	1	4	2	2	4	4
12	RL8	60S ribosomal protein L8 (RPL8)	12%	18%	23%	1	2	4	2	4	5
13	**KAP1**	Transcription intermediary factor 1-beta (TRIM28)	6.70%	18%	24%	2	9	10	2	14	17
14	RS9	40S ribosomal protein S9 (RPS9)	4.60%	18%		1	4		1	4	0
15	RL7A	60S ribosomal protein L7a (RPL7A)	7.50%	16%	21%	1	3	6	1	3	6
16	LMNB1	Lamin-B1 (LMNB1)	5.50%	16%	20%	3	7	8	3	8	11
17	CKAP4	Cytoskeleton-associated protein 4 (CKAP4)		12%	9.60%		5	4		6	4
18	DDX1	ATP-dependent RNA helicase DDX1 (DDX1)	1.20%	7.20%	13%	1	3	5	1	4	5
19	HS90A	Heat shock protein HSP 90-alpha (HSP90AA1)	3.80%	6.40%	9.40%	1	2	4	3	4	6
20	LAP2A	nuclear lamina-heterochromatin anchoring protein		6.10%	11%		2	5		2	6
21	C1TC	C-1-tetrahydrofolate synthase, cytoplasmic (MTHFD1)		5.80%	9.20%		4	7		4	8
22	SYLC	Leucyl-tRNA synthetase, cytoplasmic (LARS)		3.10%	9.10%		2	6		3	8
23	NSUN2	tRNA (cytosine(34)-C(5))-methyltransferase (NSUN2)		3.00%	8.90%		2	4		3	5
24	NMD3	60S ribosomal export protein NMD3 (NMD3)		2.80%	12%		1	4		1	4
25	NOP56	Nucleolar protein 56 (NOP56)			12%			4			5
26	SRP68	Signal recognition particle 68 kDa protein (SRP68)			11%			5			6

Known of the (SETDB-MCAF1-KAP1) and C11orf46 in magenta

Protein ID	UniProt Protein ID
Description	HUGO Gene Symbol and gene name
Sequence Coverage (%)	Percent of the residues in each protein sequence that have been identified.
total number of unique peptides	Number of unique peptides obtained for each protein
Spectral counts	Number of spectral counts adjusted for shared peptides

References
SETDB1
MCAF1
KAP1 Schultz et al, Genes and development, 2002 (PMID: 11959841)
Ivanov et al, Molecular Cell, 2007 (PMID: 18082607)

Supplementary Table 2. The complete list of proteins identified by mass spectrometry analysis of purified Flag-SETDB1 complexes

S.No	Protein ID	Description	Percent Sequence coverage (%)		Exclusive Unique Peptide Count		Total Spectrum count	
			HEK293 (Wt; +Dox)	HEK293 (Tet-ON SETDB1; clone #1; +Dox)	HEK293 (Wt; +Dox)	HEK293 (Tet-ON SETDB1; clone #1; +Dox)	HEK293 (Wt; +Dox)	HEK293 (Tet-ON SETDB1; clone #1; +Dox)
1	SETDB1	Histone-lysine N-methyltransferase SETDB1 (SETDB1)	17%	72%	14	96	28	550
2	KAP1	Transcription intermediary factor 1-beta (TRIM28)		62%		32		95
3	C11orf46	Uncharacterized protein C11orf46 (C11orf46)		53%		15		53
4	MCAF1	Activating transcription factor 7-interacting protein 1 (ATF7IP)		51%		46		127
5	RL38	60S ribosomal protein L38 (RPL38)		50%		4		5
6	CI140	Protein C9orf140 (C9orf140)		49%		16		21
7	CDK1	Cyclin-dependent kinase 1 (CDK1)		47%		12		15
8	HSP7C	Heat shock cognate 71 kDa protein (HSPA8)		46%		24		39
9	RL40	Ubiquitin-60S ribosomal protein L40 (UBA52)		45%		7		28
10	HSP71	Heat shock 70 kDa protein 1A/1B (HSPA1A)		41%		18		24
11	RL23	60S ribosomal protein L23 (RPL23)		38%		4		7
12	FHL1	Four and a half LIM domains protein 1 (FHL1)		36%		9		12
13	GRP78	78 kDa glucose-regulated protein (HSPA5)		33%		15		20
14	HP1-γ	Heterochromatin protein 1-gamma		30%		5		13
15	PRDX4	Peroxiredoxin-4 (PRDX4)		30%		6		8
16	CDC23	Cell division cycle protein 23 homolog (CDC23)		28%		11		16
17	E2F7	Transcription factor E2F7 (E2F7)		27%		17		23
18	EFTU	Elongation factor Tu, mitochondrial (TUFM)		27%		9		10
19	ADT2	ADP/ATP translocase 2 (Slc25a5)		27%		8		8
20	POGZ	Pogo transposable element with ZNF domain (POGZ)		25%		24		35
21	RL3	60S ribosomal protein L3 (RPL3)		24%		7		8
22	PCM1	Pericentriolar material 1 protein (PCM1)		24%		37		52
23	SERA	D-3-phosphoglycerate dehydrogenase (PHGDH)		23%		9		9
24	MCAF2	Activating transcription factor 7-interacting protein 2 (ATF7IP2)		23%		11		13
25	ZN828	Zinc finger protein 828 (ZNF828)		23%		16		19
26	ADNP	Activity-dependent neuroprotector homeobox protein (ADNP)		19%		17		23
27	CAMP3	Calmodulin-regulated spectrin-associated protein 3 (KIAA1543)		18%		13		16
28	NAMPT	Nicotinamide phosphoribosyltransferase (NAMPT)		18%		6		7
29	ATPA	ATP synthase subunit alpha, mitochondrial (ATP5A1)		17%		7		7
30	RLA0	60S acidic ribosomal protein P0 (RPLP0)		17%		4		6
31	TIM50	Mitochondrial import inner membrane translocase subunit TIM50 (TIMM50)		17%		5		6
32	IQGA3	Ras GTPase-activating-like protein IQGAP3 (IQGAP3)		17%		20		24
33	EXOC4	Exocyst complex component 4 (EXOC4, SEC8)		17%		12		15
34	ST38L	Serine/threonine-protein kinase 38-like (STK38L)		16%		4		7
35	EF1A1	Elongation factor 1-alpha 1 (EEF1A1)		14%		5		6
36	PYR1	CAD protein (CAD)		13%		24		26
37	SPAG5	Sperm-associated antigen 5 (SPAG5)		12%		10		11
38	MYO5C	Myosin-Vc (MYO5C)		12%		17		19
39	RUVB1	RuvB-like 1 (RUVBL1, Pontin)		12%		4		4
40	IRS4	Insulin receptor substrate 4 (IRS4)		12%		11		13
41	GRP75	Stress-70 protein, mitochondrial (HSPA9)		9.90%		6		6
42	CC123	Coiled-coil domain-containing protein 123, mitochondrial (CCDC123, CEP89)		9.70%		7		7
43	RPB2	DNA-directed RNA polymerase II subunit RPB2 (POLR2B)		9.50%		9		9
44	EXOC5	Exocyst complex component 5 (EXOC5, SEC10)		7.60%		5		5
45	UBP7	Ubiquitin carboxyl-terminal hydrolase 7 (USP7)		5.50%		4		6
46	UBR5	E3 ubiquitin-protein ligase UBR5 (UBR5)		5.40%		9		12
47	PRKDC	DNA-dependent protein kinase catalytic subunit (PRKDC)		5.30%		18		20
48	IQGA1	Ras GTPase-activating-like protein IQGAP1 (IQGAP1)		5.20%		6		8
49	EF2	Elongation factor 2 (EEF2)		4.80%		4		5
50	GCN1L	Translational activator GCN1 (GCN1L1)		4.60%		8		15
51	AZI1	5-azacytidine-induced protein 1 (AZI1)		4.00%		4		4
52	SYEP			3.40%		4		4

Known of the (SETDB-MCAF1-KAP1) and C11orf46 in magenta

Protein ID	UniProt Protein ID
Description	HUGO Gene Symbol and gene name
Sequence Coverage (%)	Percent of the residues in each protein sequence that have been identified.
total number of unique peptides	Number of unique peptides obtained for each protein
Spectral counts	Number of spectral counts adjusted for shared peptides

References
SETDB1
MCAF1
KAP1 Schultz et al, Genes and development, 2002 (PMID:)
Ivanov et al, Molecular Cell, 2007 (PMID:)

Supplementary Table 3. Differentially expressed genes in lymphoblastoid cells in WAGR patients with *C11orf46* haploinsufficiency

Cell line genotypes-->	Healthy (**C11orf46 +/+**, PAX6 +/+, BDNF +/+)	WAGR (**C11orf46 +/+**, PAX6 +/-, BDNF +/+)	WAGR (**C11orf46 +/-**, PAX6 +/-, BDNF +/+)	WAGR (**C11orf46 +/-**, PAX6 +/-, BDNF +/-)
	**1**	**2**	**3**	**4**
APBA1	0	0.037939914	0.013183187	0
SLC35F4	0.893425838	0.483859175	0.017697806	0.031326568
EFEMP1	0.354116473	0.626822534	0.018939601	0
B3GAT1	0.241510017	0.415841381	0.029555724	0.004650316
CACNA1C	0.144440766	0.155439845	0.033388941	0.01251554
CNTNAP2	0.067339067	0.148996333	0.033764714	0.003187537
FRMPD4	0.209618462	0.26745894	0.035403963	0
MAGI2	0.169096259	0.203768949	0.035699847	0.007582999
CSMD3	0.134159184	0.223125506	0.03830925	0.005166515
EGFR	0.511706458	0.280269334	0.03895468	0.00424326
ERBB4	0.255125296	0.456299248	0.040358886	0.006123303
TC2N	0.502134508	0.772895359	0.048829477	0.138291482
GABRG2	0.216343725	0.273311761	0.049121956	0
SHANK1	0.259576219	0.577913082	0.049582859	0
PLXNA4	0.285652224	0.412201452	0.050416913	0.009735498
COBL	0.202073517	0.252089297	0.057524963	0.003702687
RIMS1	0.255996931	0.287083033	0.061612945	0
NDRG1	0.49990157	0.527628602	0.064932194	0.035156681
SCN4B	0.364267519	0.61165938	0.066417684	0.009405179
CREG2	0.444776893	0.581414588	0.068216999	0.014861499
RBFOX1	0.4156812	0.624486455	0.068524321	0
MSX1	1.764791779	2.03730179	0.070791226	0.275673795
ADCY2	0.223995125	0.394031684	0.072736794	0
SLC6A17	0.549040429	1.105122765	0.073412334	0
LRP1B	0.293665909	0.46574584	0.074862552	0.024943596
CDR1	0.037914051	0.262611256	0.07604246	0
NAV3	0.300603594	0.524980857	0.077295735	0.008756466
SCN2A	0.230923693	0.366834694	0.079171449	0
DPP6	0.330752194	0.385802582	0.088449918	0.017613405
ATXN7L1	1.127594506	1.04136945	0.090462567	0.096075694
PLCB1	0.298135482	0.461996676	0.090501533	0.015256679
DCLK1	0.422936186	0.604671171	0.09135146	0.00739198
PSD2	0.632126282	0.72973595	0.091449778	0.011772625
CSRNP3	0.461528515	0.567169253	0.092566641	0.078225419
P2RX7	2.184033245	1.878791923	0.094711665	0.335294817
PBX1	0.244300203	0.376031701	0.095347776	0.022503117
NUAK1	0.400402244	0.708753105	0.096368193	0.015162631
ASTN1	0.391376766	0.621973778	0.097865927	0.011548714
GRIN2B	0.405079061	0.696209252	0.098221367	0.001717135
ARPP21	0.426333963	0.449369182	0.104096491	0
SLC22A17	0.470074856	0.845542152	0.105243618	0.012419322
NEBL	0.260311311	0.486347238	0.107166738	0.007782314
RYR2	0.428813528	0.570953339	0.107638344	0.05080765
RGS4	0.754306164	1.04940421	0.10861681	0
NRXN3	0.590043676	0.455261871	0.108681734	0.00684002
CELF4	0.443899708	0.768666605	0.109134667	0.008133781
SGIP1	0.437450057	0.561682373	0.111014588	0
MYT1L	0.504100947	0.771794895	0.111861068	0
NFASC	0.373766488	0.497472874	0.114651767	0.008742161
SLC8A1	0.313600327	0.502401855	0.114670031	0
KCNQ3	0.373514566	0.520844274	0.115708891	0
DLGAP1	1.846573391	1.80169775	0.117770964	0.5178691
AGAP1	0.453885161	0.547259392	0.118680905	0.024827037
THRB	0.708834728	1.091052422	0.120627143	0.036603416
GLYATL2	4.172897211	4.02416983	0.122478835	0.404688804
LRP1	0.291746614	0.518148639	0.124230295	0.028044937
ADCYAP1R1	0.438098193	0.695402192	0.131800994	0
ANKS1B	0.511970039	0.605679334	0.132385292	0.057681924
ADD2	0.435453633	0.639017961	0.133225814	0.008384733
PCDH1	0.649651455	0.649361904	0.139831559	0.018000969
CADM2	0.46813796	0.5404254	0.142716245	0.005318304
SEZ6L	0.49063137	0.641911076	0.146949474	0
RAPGEF4	0.924974679	1.188973783	0.147361732	0.022357915
SCN8A	0.482230683	0.66514118	0.148218388	0.042688872
CNTLN	1.576652598	2.322715544	0.149209272	0.261711614
FXYD6	0.427885568	0.78822996	0.153378692	0.010342585
TRIM9	0.517599914	0.712201531	0.155194627	0.071275383
CADM3	0.69293423	0.982374531	0.156046164	0
SHC3	0.763042714	1.174490426	0.158708096	0
FMN2	0.588749543	0.774368088	0.158734221	0.016447175
FAM13A	1.397878014	1.062202749	0.158992325	0.410082905
ERC2	0.506991109	0.898333753	0.160803969	0.006697322
PTPRD	0.464947158	0.822037131	0.161301279	0.014275824
LONRF2	0.627677145	0.929967658	0.161570443	0.041945629
LINGO1	0.849094535	1.321627844	0.168385494	0.086707236
SATB1	0.509208695	0.491207151	0.173480474	0.04358479
SRGAP3	0.58817067	0.499259514	0.17645435	0.082822386
DNM1	0.490456518	0.73654841	0.177585785	0.009861691
CHD5	0.477600121	0.698738895	0.182095934	0.005372079
PHYHIPL	1.039774096	1.714758042	0.183334228	0.025961328
CAP2	1.028693586	1.395358471	0.185688285	0.043824464
ST8SIA3	0.651048475	0.952896085	0.18653969	0
WASF3	0.715728708	1.045883329	0.18897774	0.005146247
BSN	0.758071883	0.978281556	0.191932575	0.044206662
PCDH9	0.590934915	0.856655644	0.196423689	0.024037552
DIRAS2	0.958094385	1.244293155	0.200167252	0
MRAS	0.705362182	1.085707411	0.200417579	0
KIF5C	0.776489232	1.224481385	0.203577589	0.121077269
ANK3	0.608003633	1.072856453	0.206536593	0.030076636
NTM	0.860577015	1.272874292	0.210358631	0.00763799
CAMK2B	0.653442329	0.774194656	0.211532061	0.02604724
BACE2	0	0.044901547	0.218430563	0.29274171
UNC13A	0.564313782	0.844475067	0.224686846	0.028492952
HLF	0.658789661	0.899535704	0.227321113	0
GNAO1	0.76141031	1.087889705	0.227356795	0.019394706
SLC4A10	0.668984645	0.693627053	0.228594713	0
PSD3	1.039061327	1.263386911	0.231829282	0.073340369
SYN2	0.847615548	1.168499944	0.237673163	0.060767919
GABRA1	0.687848908	0.887482149	0.238046343	0

RPH3A	0.712622546	0.978863682	0.238815419	0.010247827
CTNND2	0.851795537	1.337322536	0.240543696	0.007741492
SLC6A1	0.829675254	1.299070697	0.244824468	0
PCLO	0.856702904	1.182539991	0.262453371	0.015098329
SPOCK1	0.901966869	1.336263035	0.265324877	0.18785871
NPTX1	1.158185743	1.610875651	0.268675039	0.063410245
ARNT2	0.942468572	1.31815347	0.27142276	0.010295154
KIF1A	1.154319783	1.730148497	0.273265475	0.058044274
KCTD19	0	0	0.274420274	0.726820497
CAV1	1.649790649	2.341000501	0.275742403	0.546657047
RAB6B	0.817740737	1.078870889	0.281160822	0.084052233
PLCB2	2.736027527	2.166885271	0.285861712	0.513227207
LRIG1	0.066505947	0.26871408	0.298788905	0.536436287
SLC17A7	1.135536237	1.661046403	0.299505868	0.008835832
LRP6	0.108581559	0.083565441	0.312147051	0.305816639
FBXL16	1.019986826	1.109816192	0.319793905	0.039957277
KIAA1217	1.164089775	1.084066248	0.329817546	0.253186613
ZNF532	1.616162968	1.323623943	0.335919205	0.295635144
CNTN1	0.998760964	1.484467495	0.3455466	0
TRIM2	1.166073295	1.46642497	0.350313291	0.18602497
ATRNL1	0.999701369	1.192539082	0.351361988	0.040561272
PEG3	1.335767436	2.275221222	0.351819726	0.005726434
RTN1	1.333186639	1.912944022	0.362564178	0.008556911
APLP1	1.215225193	1.860653905	0.36944655	0.108991778
PLP1	1.315754187	2.01635224	0.370378575	0.013112009
TSPAN7	1.323310106	2.37827143	0.386051186	0.034167112
OLFM1	1.252618826	1.939812091	0.392166973	0.069416768
ZNRF1	1.505192989	1.040122911	0.399684822	0.267937298
NLRP7	0	0	0.421781141	0.271486248
GPRC5B	1.592336126	2.056858909	0.42207158	0.00796908
SLC8A3	2.453549632	2.365673146	0.43100153	0.446733772
FAIM2	1.544229228	2.789895938	0.433606485	0
DNAJC6	0.98860316	1.350876985	0.437024349	0.053481307
PLEKHB1	1.175573847	1.729207905	0.456347249	0.053851468
GAP43	1.747879544	3.673390817	0.467418827	0.152745162
NMNAT2	1.736220133	2.622190803	0.481755781	0.059321545
SYN1	1.370461016	2.472000798	0.530100702	0.013901062
NDRG4	1.231347929	1.49983594	0.579494465	0.154207116
ATP1A3	1.781095474	2.707567933	0.580050278	0.110186359
STMN2	2.395768349	2.514282268	0.603613585	0
DHRS9	2.04527602	1.497280995	0.630325074	0.056671849
BCAN	1.65974891	1.35765031	0.646754152	0.285528545
CST3	4.505831028	3.448250445	0.649861537	1.380369807
MEG3	2.061199353	3.522998532	0.65668974	0.020215559
ZNF385A	2.75099895	1.613371266	0.660925946	0.409462579
SYT1	2.066849997	2.943013257	0.664417187	0
GTSF1	26.9225139	41.58226585	0.713521705	0.631495944
S100B	2.48691825	2.38623193	0.725512286	0
AQP4	2.020458445	3.368089748	0.725979076	0
LGSN	0	0.03708638	0.73453647	0.428834511
STXBP1	2.518994103	3.637851543	0.801110578	0.068407543
FMNL2	2.948493583	2.509359521	0.840286095	0.48492508
SEMA6A	2.5184848	2.562433248	0.859561589	0.458266854
CHN1	2.66245342	4.06189862	0.860431676	0.080159714
PTN	2.73012155	4.56995454	0.876108574	0
CPE	2.561274885	3.814603097	0.91324989	0.089807063
MT3	2.138501089	3.379791619	0.939515561	0
NID1	13.82911785	9.703935247	0.940543042	3.316085998
CAMK2N1	3.07606919	5.450463985	1.047385287	0.040634691
CLIP3	3.087215948	4.007629714	1.104095391	0.366218538
KLHL3	3.661191112	2.823761465	1.259718337	1.142914963
TMEM173	3.194731508	3.800229299	1.266887132	0.607198385
DMD	2.411306589	2.481015561	1.291649743	0.619520563
MAP1A	3.977547452	6.008880653	1.362542946	0.079977208
DPYD	4.311592022	3.360140846	1.368469218	0.383445356
ENG	2.923679189	3.882781391	1.382542745	0.364550492
ATP1B2	4.482549572	5.372481734	1.431599117	0.186505914
GAS7	8.015212486	6.815379626	1.519347922	3.33245203
NRGN	7.431269892	8.290059574	1.547780659	1.217643821
MAP2	5.336590489	8.178376935	1.577507045	0
TMOD2	4.493424377	3.354562018	1.657351816	1.287609158
GPM6B	3.304592043	4.851829725	1.732170857	0.875355382
KIF5A	6.162395828	10.29584187	1.761809424	0.101830108
ENC1	5.316969975	5.70482757	1.863798353	1.315095513
TUBB2A	7.446971681	6.940038465	2.585293926	1.507448221
SNAP25	10.49018098	15.1495873	2.626466241	0.031653178
MYADM	19.80120387	11.17965892	2.797361036	0.643341146
SLC1A2	8.954411229	14.99558865	3.371295064	0
CD38	30.33884313	22.87915172	3.497427343	10.41283227
SPARCL1	14.95682604	24.23814912	5.600386102	0.091682241
TUBB2B	22.36033012	23.18011147	5.848503913	1.380310824
EPB41L2	20.82308944	15.42229558	7.954683825	5.153390367
ALOX5AP	24.40609273	29.78024406	9.75721863	5.669250231
CALCRL	0.137012643	0.351487613	0.977066398	1.214965031
TBC1D30	0.529968114	0.206015312	1.11499455	0.847816895
NT5E	0.381729173	0.369213197	1.158771002	1.763962881
TESC	0.534202288	0.224251287	1.499994769	2.662010056
PTPRJ	0.792413523	0.190811737	1.630258645	2.534990141
TSPAN12	0.347142088	0.689283073	1.648712515	2.358347351
PHACTR2	0.354169056	0.452148856	1.86861337	2.082781347
HENMT1	0.674709141	0.111270605	2.22703477	1.828665789
ANKRD36BP2	0.023261369	0.624338505	2.27672751	3.7326996
KLHL6	0.221728533	1.31054953	2.348072329	3.078166478
UST	0.297226054	0.899216701	2.540758064	3.004055128
CMTM3	0.266141536	0.290168971	2.633353028	3.653412669
FAM129C	0.635037476	0.358402819	2.694509723	4.484936022
POMC	0.243261286	0	2.781018116	3.229936145
GIMAP8	1.67663655	0.825827877	3.237209663	3.904739856
CYB5R2	0.505971305	0.389400517	3.247370423	2.385775415
HMX3	0.0797391	0	3.454466977	2.060876873
TP53INP1	1.244153331	1.13189415	3.496782236	2.377551818
SETBP1	1.123984505	1.108722618	3.509342813	2.62290263
AMPD3	1.487070241	1.669009931	3.562487934	2.784370632
LBH	2.186240401	0.761910438	3.574059329	3.555658682

PIGR	0.096184378	1.073354496	4.038302946	6.805138441
GPR55	1.78914847	0.549035567	4.16074144	3.923633635
FYN	1.937317976	0.998033214	4.348822205	3.05043245
ALDH5A1	1.362802557	2.257256634	4.617766113	5.409148513
PLAGL1	1.249765666	1.938112134	4.625909037	4.14972288
LIPH	1.350138495	0.864444646	4.842380889	3.248113634
BLK	1.791614557	1.573680958	5.077571785	3.053058715
IGF2BP3	1.357237057	3.888491238	5.752878782	6.155098191
ZBTB32	2.633808381	3.761653431	5.824303378	7.969478809
PLA2G16	0.690398324	1.836579181	6.140843495	5.831325958
TP63	1.305008356	0.333172911	6.306915097	5.716413563
SMARCB1	5.014114169	4.156309153	6.956429201	9.293977705
PTPN7	4.497738887	6.261788137	8.042583251	10.79059694
CDK5R1	2.270766764	3.707418205	8.2932944	7.511886384
ZNF589	5.082446892	4.635129103	8.453916136	9.719463379
IL17RB	3.479044755	2.683430912	8.546211313	10.06459305
F8A1	3.767567739	1.76494796	9.680999739	5.815439878
EML4	4.448935122	8.236847459	10.80639879	13.50021
WFDC2	0.541441183	2.031405113	11.3481101	15.7236702
GRN	12.56862645	9.829427521	17.93384132	20.8332571
MPEG1	11.5917261	6.854910524	22.39166951	32.60513167
IFNGR1	10.25926796	6.845682676	36.06002062	22.34925796
CTSC	13.7708923	12.33306977	48.21569872	31.70532809
CCR7	3.337834851	24.60480565	48.78413049	39.69910496

Lymphocyte Sample Information:

NIH ID	Manuscript ID	Diagnosis	Age (years)	Sex	Race/Ethnicity	Chr	Cytoband	Start (Hg18, March 2006, Build 36)	Stop (Hg18,March 2006, Build 36)	SizeMb	*BDNF*	*C11orf46*	*PAX6*	
H23.3	1	Healthy	29	M	White/Non-Hispanic	no deletion	N/A	N/A	N/A	N/A	+/+	+/+	+/+	
W42.3	2	WAGR	13	M	White/Non-Hispanic	chr11	p14.1 - p13	30,406,179	35,176,519	4.77	+/+	+/+	+/-	
W39.3	3	WAGR	12	M	White/Non-Hispanic	chr11	p14.1 - p12	28,901,513	39,755,713	10.85	+/+	+/-	+/-	
W15.3	4	WAGR	17	M	White/Non-Hispanic	chr11	p14.2 - p12	26,005,134	40,174,102	14.17	+/-	+/-	+/-	
824.3	A	Healthy	8	M	Asian	no deletion	N/A	N/A	N/A	N/A	+/+	+/+	+/+	
A4.3	B	Aniridia	8	M	White/Non-Hispanic									heterozygous W266X (premature stop-codon in exon 10)
W45.3	C	WAGR	7	M	White/Hispanic	chr11	p15.1 - p13	19,309,352	32,405,969	13.10	+/-	+/-	+/-	
H24.3	D	Healthy	24	F	Asian	no deletion	N/A	N/A	N/A	N/A	+/+	+/+	+/+	
A5.3	E	Aniridia	28	F	White/Non-Hispanic									heterozygous deletion of exons 1-4
W8.3	F	WAGR	25	F	White/Non-Hispanic	chr11	p14.1 - p12	30232199	37070116	6.837917	+/+	+/-	+/-	

Supplementary Table 4. Genes with KAP1 binding sites

	GENE	KAP1 Binding SITES					
#		**Chr**	**Start**	**End**	**Strand**	**Annotation**	**Distance to TSS**
1	***DCLK1***	chr13	36429427	36429776	+	promoter-TSS (NM_001195415)	-203
		chr13	36430027	36430376	+	intron (NM_004734, intron 5 of 17)	397
		chr13	36428827	36429176	+	intron (NM_004734, intron 5 of 17)	997
2	***SEMA6A***	chr5	115883857	115884206	+	promoter-TSS (NM_020796)	-652
		chr5	115882030	115882775	+	intron (NM_020796, intron 1 of 18)	26520
		chr5	115880296	115881879	+	intron (NM_020796, intron 1 of 18)	28149
		chr5	115910979	115911428	+	intron (NM_020796, intron 1 of 18)	29464
3	***GAP43***	chr3	115342328	115342777	+	exon (NM_002045, exon 1 of 3)	401
		chr3	115580855	115581204	+	Intergenic	161810
		chr3	115503737	115504186	+	Intergenic	167517
		chr3	115509294	115510043	+	intron (NM_002338, intron 3 of 6)	238878
4	***PLXNA4***	chr7	132330825	132331512	+	intron (NM_181775, intron 1 of 4)	2279
		chr7	132249535	132250369	+	intron (NM_181775, intron 2 of 4)	11371
		chr7	132321034	132321383	+	intron (NM_181775, intron 2 of 4)	12239
		chr7	132172893	132173342	+	intron (NM_181775, intron 4 of 4)	88206
		chr7	132159928	132160277	+	intron (NM_001105543, intron 3 of 3)	101221
		chr7	132088492	132089041	+	intron (NM_001105543, intron 3 of 3)	172557
		chr7	132086292	132086941	+	intron (NM_001105543, intron 3 of 3)	174707
		chr7	132064009	132065211	+	intron (NM_020911, intron 3 of 31)	196713
		chr7	131908018	131908567	+	exon (NM_020911, exon 9 of 32)	353031
		chr7	131871293	131872898	+	intron (NM_020911, intron 15 of 31)	389228
		chr7	131831114	131831463	+	exon (NM_020911, exon 28 of 32)	430035
		chr7	131760741	131761090	+	Intergenic	500408
5	***NUAK1***	chr12	106413007	106413716	+	intron (NM_014840, intron 1 of 6)	7159
		chr12	106409791	106410440	+	intron (NM_014840, intron 1 of 6)	32756
		chr12	106386100	106386549	+	Intergenic	120450
		chr12	106500881	106501230	+	Intergenic	123696
		chr12	106526389	106526915	+	Intergenic	147487
6	*ATXN7L1*	chr7	105305401	105306250	+	intron (NM_020725, intron 3 of 11)	13784
		chr7	105353769	105354703	+	intron (NM_020725, intron 3 of 11)	-34627
		chr7	105278867	105279216	+	exon (NM_138495, exon 5 of 10)	40568
		chr7	105331052	105331901	+	intron (NM_020725, intron 3 of 11)	-11867
7	*CAP2*	chr6	17394551	17394900	+	intron (NM_006366, intron 1 of 12)	989
8	*CD38*	chr4	15742959	15743628	+	Intergenic	-36638
9	*CTNND2*	chr5	11589741	11590090	+	promoter-TSS (NM_001288716)	-886
10	*CTSC*	chr11	88034855	88035255	+	intron (NM_001814, intron 4 of 6)	35886
11	*DPP6*	chr7	153126424	153126873	+	Intergenic	-457771
12	***ENC1***	chr5	73839508	73840057	+	Intergenic	97467
13	*EPB41L2*	chr6	131375467	131376410	+	intron (NM_001431, intron 1 of 19)	8524
14	*ERC2*	chr3	56317713	56318062	+	intron (NM_015576, intron 3 of 16)	184504
		chr3	56322578	56322927	+	intron (NM_015576, intron 3 of 16)	179639
15	*FMNL2*	chr2	153193442	153193891	+	intron (NM_052905, intron 1 of 25)	1915
		chr2	153311507	153311956	+	intron (NM_052905, intron 1 of 25)	119980
		chr2	153290131	153290480	+	intron (NM_052905, intron 1 of 25)	98554
		chr2	153308234	153308583	+	intron (NM_052905, intron 1 of 25)	116657
16	*GPR55*	chr2	231801432	231802499	+	Intergenic	-12024
17	*KIAA1217*	chr10	24849930	24850661	+	Intergenic	94861
		chr10	24848655	24849779	+	Intergenic	93783
		chr10	23982588	23983337	+	promoter-TSS (NM_001098500)	-713
		chr10	24495830	24496179	+	intron (NM_001098500, intron 2 of 18)	-1716
		chr10	24160260	24160609	+	intron (NM_001098500, intron 1 of 18)	176759
18	*MEG3*	chr14	101299332	101300245	+	intron (NR_033360, intron 4 of 6)	7319
19	***MSX1***	chr4	4727235	4727993	+	Intergenic	-133778
		chr4	4858104	4858453	+	Intergenic	-3114

20	***NDRG1***	chr8	134415298	134415847	+	Intergenic	-106025
21	*NID1*	chr1	236139620	236140869	+	3' UTR (NM_002508, exon 20 of 20)	88237
22	***NMNAT2***	chr1	183395942	183397220	+	Intergenic	-8947
		chr1	183398905	183399377	+	Intergenic	-11507
23	*NRXN3*	chr14	79747740	79748289	+	intron (NM_001272020, intron 1 of 5)	2332
		chr14	79115279	79115628	+	intron (NR_073547, intron 5 of 20)	245379
		chr14	78459136	78461053	+	Intergenic	-176622
24	*PSD3*	chr8	18870380	18870929	+	intron (NM_015310, intron 1 of 15)	542
		chr8	18473281	18473630	+	intron (NM_206909, intron 8 of 12)	192950
25	*RAPGEF4*	chr2	173865496	173866245	+	intron (NM_001282901, intron 10 of 24	72937
		chr2	173538960	173539409	+	Intergenic	-61341
26	***SLC8A3***	chr14	70656506	70657019	+	promoter-TSS (NM_058240)	-975
27	*SPOCK1*	chr5	136368969	136369429	+	intron (NM_004598, intron 6 of 10)	465819
28	***SRGAP3***	chr3	9289652	9290101	+	intron (NM_001033117, intron 1 of 21)	1493
		chr3	9290556	9290905	+	5' UTR (NM_014850, exon 1 of 22)	639
29	*TC2N*	chr14	92363123	92363616	+	intron (NM_006329, intron 4 of 10)	-29489
30	*TRIM9*	chr14	51615755	51616104	+	Intergenic	-53507
31	***PTN***	chr7	137027601	137028150	+	intron (NM_002825, intron 1 of 4)	671
		chr7	137028301	137028685	+	promoter-TSS (NM_002825)	53
32	***MAP2***	chr2	210479508	210481177	+	intron (NM_002374, intron 2 of 14)	35939
		chr2	210361422	210365296	+	intron (NM_001039538, intron 1 of 14)	74588
33	***PLEKHB1***	chr11	73344057	73344406	+	Intergenic	-12992
34	*SLC6A1*	chr3	10948383	10949867	+	intron (NM_014229, intron 6 of 13)	-85295
35	*COBL*	chr7	51215907	51216456	+	intron (NM_001287436, intron 5 of 13)	168334
		chr7	51459755	51460104	+	Intergenic	-75414
		chr7	51147851	51148200	+	intron (NM_001287438, intron 7 of 7)	236490
		chr7	51213617	51214566	+	intron (NM_001287436, intron 5 of 13)	170424
36	*DNAJC6*	chr1	65730594	65730943	+	intron (NM_014787, intron 1 of 18)	391
37	*DPP6*	chr7	153126424	153126873	+	Intergenic	-457771
38	*EGFR*	chr7	55134099	55134548	+	intron (NM_201282, intron 1 of 15)	47598
39	*ERBB4*	chr2	213586438	213587195	+	Intergenic	-183464
		chr2	213401560	213402130	+	intron (NM_001042599, intron 1 of 26)	1507
40	***SHC3***	chr9	91725340	91726189	+	intron (NM_016848, intron 2 of 11)	67918
41	*SYN1*	chrX	47467934	47468372	+	intron (NM_006950, intron 1 of 12)	11103

Supplementary Table 5. sgRNA sequences

HUMAN sgRNAs<br>**Gene**	**Promoter location**	**sgRNAs score**	**sgRNA Sequence (5' to 3')**	**Total no. of offtarget sites (hg19)**	**off-target sites in exons (mm9)**	**score**	**mismatches**	**UCSC gene**	**locus**
**DCLK1**	GRCh38:13:35768052:36131906:-1								
	chr13:-36705474	73	TTGGCAGGGCGCACCCACCA**CGG**	125 (17 are in genes)	TTGGTGGGGGGCACCCACCAGAG	1.4	3MMs [5:6:10]	NM_001165937	chr17:-37816592
					TGGGTAGAGCCCACCCACCAGGG	0.8	4MMs [2:5:8:11]	NR_048548	chr1:-44476336
					TCGTCGGGGCGCACCCGCCACGG	0.4	4MMs [2:4:6:17]	NM_139057	chr15:+100881552
					TTGGCCTGTGGCACCCACCAGGG	0.3	4MMs [6:7:9:10]	NM_001252617	chr9:-138418498
					CTGCCAGGGCGGCCCCACCAGGG	0.3	4MMs [1:4:12:13]	NM_016621	chr11:+45951948
					TTGGCAGTGAGAACCCACAAGAG	0.2	4MMs [8:10:12:19]	NM_001191320	chr15:-26961770
					CAGGCAGGGTGCAGCCACCAGAG	0.2	4MMs [1:2:10:14]	NM_133476	chr12:+6797421
					TTGGCACTGAGCACCCAGCAAGG	0.2	4MMs [7:8:10:18]	NM_015299	chr14:-24901106
					CAGGCAGGGCCCACCCAGCATGG	0.2	4MMs [1:2:11:18]	NM_013433	chr19:-12822337
					GTGGCAGGGTGCACTCACCCGGG	0.2	4MMs [1:10:15:20]	NM_001171611	chr10:-88428532
					TCGGCAGGCCACACGCACCAAGG	0.1	4MMs [2:9:11:15]	NR_027033	chr22:-46508498
					GTGGCAGAGCCCAGCCACCAGGG	0.1	4MMs [1:8:11:14]	NR_024369	chr9:+34568012
					TTGGCCGGACCCACCCTCCAGGG	0.1	4MMs [6:9:11:17]	NM_004260	chr8:+145741111
					CTGGCGGGGCGCACTCACCCAAG	0.1	4MMs [1:6:15:20]	NM_001145657	chr1:+21995727
					TTGGCCGGGCGGCCCCACCCAGG	0.1	4MMs [6:12:13:20]	NM_012188	chr5:-169532831
					TTGGCAGGCCACGCCCTCCAGAG	0.1	4MMs [9:11:13:17]	NR_046363	chr11:-113857828
	chr13:-36705605	66	TCTCCAGAGGAGGGCGGCGG**CGG**	300 (64 are in genes)	TCTCGAGGGGAGGGCGGCCGTGG	1	3MMs [5:8:19]	NM_138701	chr7:+40174037
					GCTCACGGGGAGGGCGGCGGGAG	0.8	4MMs [1:5:6:8]	NM_001261826	chr19:+2115460
					ACGCCTGGGGAGGGCGGCGGGAG	0.8	4MMs [1:3:6:8]	NM_138345	chr3:-183948511
					GCACCAGCGGTGGGCGGCGGCGG	0.8	4MMs [1:3:8:11]	NM_001130709	chr4:-71570661
					GCCCCGGAGCAGGGCGGCGGGAG	0.8	4MMs [1:3:6:10]	NM_133265	chrX:+112084111
					TCTTGAGTGGCGGGCGGCGGCAG	0.8	4MMs [4:5:8:11]	NM_001141980	chr15:-43785392
					CCTCCAGAGGACGGCGGCGATGG	0.8	3MMs [1:12:20]	NM_031371	chr1:+235491344
					CCTGCAGCGGAGTGCGGCGGCGG	0.6	4MMs [1:4:8:13]	NM_004690	chr6:-150039215
					CCTCCAGAGGACCGCGGCGGGGG	0.6	3MMs [1:12:13]	NM_000386	chr17:+28618785
					TCTATAAAGGCGGGCGGCGGCAG	0.5	4MMs [4:5:7:11]	NM_004152	chr19:+2269487
					GCCCCACAGGAAGGCGGCGGTGG	0.5	4MMs [1:3:7:12]	NM_198868	chr5:+179294706
					GCACCGGAGGTGGGCGGCGGGGG	0.5	4MMs [1:3:6:11]	NM_001009811	chr17:-79164420
					CATCCTGAGGAAGGCGGCGGCGG	0.4	4MMs [1:2:6:12]	NM_016538	chr17:-79875808
					TCTTCAGGGGGCGGCGGCGGCGG	0.4	4MMs [4:8:11:12]	NM_001130106	chr19:-55790883
					TCTTCAAGGGAGGGCGGCAGGGG	0.3	4MMs [4:7:8:19]	NM_024895	chr10:-102777939
					TCTCCAGGGCCAGGCGGCGGCGG	0.3	4MMs [8:10:11:12]	NM_000484	chr21:+27347416
					TCTGCTGAGAAGCGCGGCGGCAG	0.3	4MMs [4:6:10:13]	NM_153369	chr6:+111580690
					TGTCCAGAGCTGTGCGGCGGGAG	0.3	4MMs [2:10:11:13]	NM_198491	chr16:+85141797
					TCTGCACAAGATGGCGGCGGTAG	0.3	4MMs [4:7:9:12]	NM_001278193	chr11:-62414054
					TCTCCGGCTGGGGGCGGCGGCGG	0.3	4MMs [6:8:9:11]	NM_079837	chr16:-88110432
					TGTTCAGAGGAGCGCGGCGCTGG	0.3	4MMs [2:4:13:20]	NR_026967	chr11:+575796
					CCTCCAGGGGATGGCGGCCGCAG	0.3	4MMs [1:8:12:19]	NM_020902	chr19:-7682975
					TCTCGCGTGGAGGGGGGCGGGGG	0.2	4MMs [5:6:8:15]	NR_047572	chr16:-3086605
					TCAGCAGAGGAGGGAGGCGCGGG	0.2	4MMs [3:4:15:20]	NM_001943	chr18:-29077934
					TCTCCGGGGAAGGGCGGTGGCAG	0.2	4MMs [6:8:10:18]	NM_198498	chr11:-111155056
					TCTCTAGAGTAGGGGGGCGCTAG	0.2	4MMs [5:10:15:20]	NM_145064	chr12:+57643138
					TCACCAGCAGAGGGCAGCGGAGG	0.2	4MMs [3:8:9:16]	NM_003644	chr17:-9862864
					TCTATAGAGGAGAGAGGCGGAGG	0.2	4MMs [4:5:13:15]	NM_001267865	chr7:-48080904
					TCTCAAGAGCTGGGCGGTGGTGG	0.2	4MMs [5:10:11:18]	NM_001159597	chr2:-263915
					GTTCCAGAGGAGGGAGGCAGGGG	0.1	4MMs [1:2:15:19]	NM_001261438	chr17:-41611160
					CCTCCAAAGGAGGGCGACAGAGG	0.1	4MMs [1:7:17:19]	NM_001128846	chr19:-11134132
					TCTCTCGCGGAGGTCGGCGGTGG	0.1	4MMs [5:6:8:14]	NM_001730	chr13:+73633171
					TCATCAGAGGAGGGCGAAGGCAG	0.1	4MMs [3:4:17:18]	NM_016578	chr11:+77404591
					GCTCCCAAGGAGGGCAGCGGGGG	0.1	4MMs [1:6:7:16]	NM_014215	chr1:+156828786
					TCTCCCCAGGACGGCGGCTGTGG	0.1	4MMs [6:7:12:19]	NM_000613	chr11:-6452682
					TCTCCCCAGAAGGGCCGCGGCGG	0.1	4MMs [6:7:10:16]	NM_003206	chr6:+134210675
					GCTCCAGAGAAGGGCGGGTGGAG	0.1	4MMs [1:10:18:19]	NM_001146313	chr17:-12659780
					CTTCCAGAGGAGGGCTGCTGGAG	0.1	4MMs [1:2:16:19]	NM_152924	chr15:-89738670
					CCTCCAGAGGCGCGGGGCGGGAG	0.1	4MMs [1:11:13:15]	NM_080738	chr1:-236558652
					TCTCCCCAGGCGGGTGGCGGCAG	0.1	4MMs [6:7:11:15]	NM_138435	chr22:-40417505
					TCTCCAGGTGAGCGGGGCGGCAG	0.1	4MMs [8:9:13:15]	NM_001199636	chr7:+31104544
					GGTCCAGAGGAGGGAGGAGGAAG	0.1	4MMs [1:2:15:18]	NM_005986	chr13:+112724279
					TCTGCAGAGGGGTGGGGCGGGGG	0.1	4MMs [4:11:13:15]	NM_001184726	chr22:-31003138
					TCGCCCGAGGAGCGCGGTGGCGG	0.1	4MMs [3:6:13:18]	NM_005476	chr9:-36258341
					TCTGCAGGGGAGGGAAGCGGAGG	0.1	4MMs [4:8:15:16]	NM_213560	chr19:-14582366
					TCTCCAGCTGCGGCCGGCGGAGG	0.1	4MMs [8:9:11:14]	NM_001083908	chr14:+50100580
					TCTCCCCAGGTGGGCGGGGGCAG	0.1	4MMs [6:7:11:18]	NM_024979	chr13:+113678947
					TCTCCAGAAGTGGGCGTCTGCAG	0.1	4MMs [9:11:17:19]	NM_133463	chr7:+2748700
					TCTCCCGAGGCGGGAGGCGCTGG	0.1	4MMs [6:11:15:20]	NM_001098202	chr17:+1959532
					TCTCGACAGGAGGGCAGCAGTGG	0.1	4MMs [5:7:16:19]	NM_003173	chrX:-48566014
					TCTCAGGAGGAGGGAGGTGGGAG	0	4MMs [5:6:15:18]	NR_036447	chr16:+16412011
					TCTCCCGAGGTGGGCGGGGACAG	0	4MMs [6:11:18:20]	NM_001040011	chr9:-131038479
					GCTCCAGAGGAAGGGGGAGGAGG	0	4MMs [1:12:15:18]	NM_001077208	chr4:+83763407
					CCTCCAGAGCAGGCCAGCGGTGG	0	4MMs [1:10:14:16]	NM_001139442	chr9:-124751897
					TCCCCAGAGGATGACGGCCGAGG	0	4MMs [3:12:14:19]	NM_007277	chr5:+465217
					TCTCCCGGGGAGGAGGGCGGCGG	0	4MMs [6:8:14:15]	NM_006334	chr9:-137967112
					TGTCCAGAGGAGCGCCGCCGAAG	0	4MMs [2:13:16:19]	NM_004976	chr11:-17757907
					TCTACTGAGGAGGGCTGTGGCAG	0	4MMs [4:6:16:18]	NM_016240	chr8:+27528859
					TCTCCAGGGGAGACCGGCTGCAG	0	4MMs [8:13:14:19]	NM_052821	chr9:+137007180
					TCTGCAGAGGAGGACGTAGGGGG	0	4MMs [4:14:17:18]	NM_014916	chr7:+97838709
					TCTCCTGAGGATGGCAGAGGTGG	0	4MMs [6:12:16:18]	NM_175867	chr21:+45678621
					TCTTCAGAGGAGGACTGCAGAAG	0	4MMs [4:14:16:19]	NM_024582	chr4:+126412415
					TCTCCAGAGGAATGCAGGGGAGG	0	4MMs [12:13:16:18]	NM_022664	chr1:+150485306
					TCTCCAGAGCAGGCCCGGGGCAG	0	4MMs [10:14:16:18]	NM_006319	chr16:-29874190
	chr13:-36705727	86	AGATGAGGCCCCCACCGTCG**GGG**	96 (25 are in genes)	AGCTGGGGCCCGCACCGTCGGGG	0.8	3MMs [3:6:12]	NM_004473	chr9:+100616898
					AGGTGAGGACCCCAGCGTCGTGG	0.5	3MMs [3:9:15]	NM_016145	chr19:-12780142
					TGCTGCGGCCCCGACCGTCGGGG	0.3	4MMs [1:3:6:13]	NM_001080531	chr4:+146653988
					CGATGGGGCGCCCACCCTCGAAG	0.3	4MMs [1:6:10:17]	NM_178448	chr9:+139959040
					AGTTCCGGCCCCCACCGTGGCGG	0.3	4MMs [3:5:6:19]	NM_175872	chr19:+35454985
					AGATGAGGCGCCCAGCATCGGGG	0.2	3MMs [10:15:17]	NM_003482	chr12:+49434903
					AGATGTGCCCCACACCTTCGAAG	0.2	4MMs [6:8:12:17]	NM_001012753	chr19:+12090116
					TGCTGAGGGCCCCACAGTCGGAG	0.2	4MMs [1:3:9:16]	NM_001194986	chr1:+48231136
					AGATGAGGCCGCCACGGTCCCAG	0.1	3MMs [11:16:20]	NM_020695	chr19:-1827891
					AGCTGAGGTCTCCACTGTCGAGG	0.1	4MMs [3:9:11:16]	NR_047668	chr5:-10249870
					TGATGGGGCCCCCAACGTTGGGG	0.1	4MMs [1:6:15:19]	NM_017647	chr17:-61902921
					AGATGAGGCTCACAACATCGAGG	0.1	4MMs [10:12:15:17]	NM_198535	chr19:+9413240
					AGTTGAGGCCCGCACCCCCGAGG	0.1	4MMs [3:12:17:18]	NM_003685	chr19:-6418147
					TGATGAGGTCCCCACGGTGGAAG	0.1	4MMs [1:9:16:19]	NR_003587	chr17:-73616380
					AGGTGAGGCCCGCGCCGTCAGGG	0	4MMs [3:12:14:20]	NM_001145524	chr16:-30107392
					AGTTGAGGCCCCCACTCTCCGAG	0	4MMs [3:16:17:20]	NM_001010898	chr1:-110717475
					AGATGGGGCCGCCACCTACGAAG	0	4MMs [6:11:17:18]	NM_001204158	chr19:+49129517
					AGATGAGGCACCCACCTGTGTGG	0	4MMs [10:17:18:19]	NM_004455	chr1:+26361379
					AGATGAGGCCGCCACCACCTGGG	0	4MMs [11:17:18:20]	NM_032444	chr16:-3640873
					AGGTGAGGCCCCCAGAGTGGCAG	0	4MMs [3:15:16:19]	NM_001114099	chr16:-29909148
					AGGTGAGGCCCGCTCCGGCGGAG	0	4MMs [3:12:14:18]	NM_001139	chr17:-7983061
					AGGTGAGGCCCCGACAGACGGAG	0	4MMs [3:13:16:18]	NM_003978	chr15:+77325290
					AGATGGGGCCCCCAGCAGCGGGG	0	4MMs [6:15:17:18]	NM_004260	chr8:+145742541
					AGATGAGGCCCACAGCGACAAGG	0	4MMs [12:15:18:20]	NM_013245	chr16:+69350124
					AGTTGAGGCCCCCTCCGGGGGGG	0	4MMs [3:14:18:19]	NM_017811	chr9:+33817440
	chr13:-36705833	59	CAAGTAGTCTTTGGGAGATG**GGG**	246 (14 are in genes)	CAAGAAGACCTTGGGAAATGGAG	0.5	4MMs [5:8:10:17]	NM_001135608	chr5:+142604637
					CATGCACTCTCTGGGAGATGCAG	0.5	4MMs [3:5:7:11]	NM_001122741	chr6:-152163658
					CAACAAGTCATTTGGAGATGCAG	0.5	4MMs [4:5:10:13]	NM_001030059	chr10:+122349382
					CAAGTGGGCTGGGGGAGATGAGG	0.2	4MMs [6:8:11:12]	NM_006841	chr3:-50258019
					CAGGTAGCCTTTGGGAGGTACAG	0.1	4MMs [3:8:18:20]	NM_001101372	chr19:-51833004
					CAAGTAGTCTTTGGGAAAGCCGG	0.1	3MMs [17:19:20]	NM_017631	chr4:+169189021
					CAAGTGTGCTTTGGGGGATGGAG	0.1	4MMs [6:7:8:16]	NM_017594	chr9:+93373816
					CAAGCAGTATTTGGAAGAAGGAG	0.1	4MMs [5:9:15:19]	NM_018384	chr7:-150434343
					TAAGAAGTCTTTGTGAGAGGAAG	0.1	4MMs [1:5:14:19]	NM_002728	chr11:-57158120
					CAGGTATTCTTGGTGAGATGGGG	0.1	4MMs [3:7:12:14]	NM_007062	chr12:+108091464
					CAAGTTCTCTTTGGTAGATCTGG	0.1	4MMs [6:7:15:20]	NM_006845	chr1:+45226040
					CAAGTAGCATTTGGGCAATGAGG	0.1	4MMs [8:9:16:17]	NM_194071	chr7:-137563090
					CAAGCTGTCTTTGGGGGGTGGGG	0	4MMs [5:6:16:18]	NM_014443	chr5:-148756252
					CAAGTAGGCTTTGAGGGGTGAAG	0	4MMs [8:14:16:18]	NM_018416	chr12:+8206724
**GAP43**									

	chr3:+115342101	86	GGGGGCGACTGTCTAGAATG**GGG**	78 (5 are in genes)	GGGGGTGTCTGGCTAGCATGCGG	0.2	4MMs [6:8:12:17]	NM_025029	chr2:-130940041
					GCGGGTGACTGCCTAGAAGGCGG	0.2	4MMs [2:6:12:19]	NM_001040107	chr12:+111099240
					GGGGGCGCCTGCCTAGCCTGGGG	0.1	4MMs [8:12:17:18]	NM_001145028	chr19:-14168122
					GGGGGCGGCTGTCCACAGTGCAG	0	4MMs [8:14:16:18]	NM_023068	chr20:+3677444
	chr3:+115342020	77	ATCATATCTCCTGGATTGAC**TGG**	161 (12 are in genes)	TATTGCAGTGGAAAAGTAGGTGG	0.7	3MMs [1:3:18]	NM_014831	chr3:-36876240
					TACTTCAGTGGACAAGTCGGGGG	0.6	4MMs [1:3:5:13]	NR_024453	chr16:-433349
					CAAAGCAATAGAAAAGTCTGTGG	0.5	4MMs [4:8:10:19]	NM_022340	chr3:-15126381
					CGAGGCAGAGGAGAAGTCGGAGG	0.3	4MMs [2:4:9:13]	NM_031419	chr3:-101568667
					AGCTGCAGTGGAAAAGTTGGAGG	0.3	4MMs [1:2:3:18]	NM_015180	chr14:+64498031
					CAATGCAGCGGAGAAGGCGGCAG	0.2	3MMs [9:13:17]	NM_001146698	chr12:+114404285
					AAATGAAGGGGAAAAGTCAGAAG	0.2	4MMs [1:6:9:19]	NM_018723	chr16:-7102016
					CAATCCTGTGGAAAAGTCATTGG	0.1	4MMs [5:7:19:20]	NM_001101421	chr12:-109879952
					CAATGCAGTGATAAAGTCCTTGG	0.1	4MMs [11:12:19:20]	NM_006379	chr7:+80374531
					CAAAGCAGTGGAGAAGCTGGGGG	0	4MMs [4:13:17:18]	NM_212481	chr2:+97215507
					CAAAGGAGTGGAAAAATTGGTAG	0	4MMs [4:6:16:18]	NM_005657	chr15:-43712987
					CAATGCATTGGAAAACTGGCTGG	0	4MMs [8:16:18:20]	NM_001113498	chr14:+47309640
	chr3:-115341781	64	TGCATAGTTAATAATTGTGG**AGG**	245 (16 are in genes)	TGAGTGGGTAATAATTGTGGTGG	0.8	4MMs [3:4:6:8]	NM_033112	chr6:+42994990
					TTTAAAGTTAATAATTGTGTTAG	0.7	4MMs [2:3:5:20]	NM_002156	chr2:-198351291
					TTTATAGTCAAAAATTGTGGTAG	0.4	4MMs [2:3:9:12]	NM_182536	chr2:+69177259
					TTCATTGTAAATAATTGTGAAAG	0.3	4MMs [2:6:9:20]	NM_001205019	chrX:+30748573
					TTCATAATTTATAAGTGTGGGGG	0.3	4MMs [2:7:10:15]	NM_001040108	chr14:-75481940
					TGCAAAGTTTATAATTCTGTGAG	0.2	4MMs [5:10:17:20]	NM_153348	chr12:-117366009
					TGCCCAGTAAATAATTGGGGTAG	0.2	4MMs [4:5:9:18]	NM_001037132	chr7:+107850023
					TGTAAAGTAAATAATAGTGGAAG	0.2	4MMs [3:5:9:16]	NM_138571	chr6:-126300301
					TCCTTAGTTAATAACTGTAGTGG	0.1	4MMs [2:4:15:19]	NM_007007	chr12:+69665698
					TGCATAATTCATCATTTTGGCAG	0.1	4MMs [7:10:13:17]	NM_207410	chr6:-55216353
					TGCATAGTTAAAAATGCTGGGAG	0.1	3MMs [12:16:17]	NM_000216	chrX:-8498795
					TGCATATTTAATAAGTATAGGGG	0	4MMs [7:15:17:19]	NM_032663	chr12:+109509553
					TGCATAATTAAAAATTGAAGTAG	0	4MMs [7:12:18:19]	NM_031935	chr1:-185970652
					TGCAAAGTTAATACCTTTGGAGG	0	4MMs [5:14:15:17]	NM_053276	chr2:-37028384
					TGCAGAGTTAATAAACGTCGAGG	0	4MMs [5:15:16:19]	NR_027906	chr6:-168226140
					TGCATAGTTATTAATAGTATTGG	0	4MMs [11:16:19:20]	NM_032581	chr7:-22983282
	chr3:+115341818	54	AGATATGACTTATATTGTAT**AGG**	396 (22 are in genes)	TGATAAGACCTATATTGTAATAG	0.4	4MMs [1:6:10:20]	NM_018442	chr1:+167973083
					TGATTTGAGTTAAATTGTATTGG	0.4	4MMs [1:5:9:13]	NR_033698	chr8:+42940214
					AGAAATGAAGTATATTATATAAG	0.3	4MMs [4:9:10:17]	NR_015342	chr9:-79400364
					ATAGATGATTTATATTGTTTCAG	0.3	4MMs [2:4:9:19]	NM_006581	chr6:+96562066
					AGAAATGCCTTCTATTTTATAGG	0.3	4MMs [4:8:12:17]	NM_015879	chr18:+55031576
					TGATTTGAATTATAATGTATAGG	0.3	4MMs [1:5:9:15]	NM_032172	chr7:+6190255
					GGAGATGACTTAGATTGTGTAAG	0.2	4MMs [1:4:13:19]	NM_001040446	chr5:+32230405
					AGGTATGAGTTATATTTTAGGAG	0.2	4MMs [3:9:17:20]	NM_001001436	chr16:-46836257
					ATATATGATATATATAGTATAGG	0.2	4MMs [2:9:10:16]	NM_001277196	chr8:-133858979
					AGATATTCCTTACATTGTGTCGG	0.1	4MMs [7:8:13:19]	NM_001252020	chr15:-31294361
					AGTTCTGACTCATTTTGTATTAG	0.1	4MMs [3:5:11:14]	NM_014612	chr9:+96289551
					ATATAAGACTTATAATTTATAAG	0.1	4MMs [2:6:15:17]	NM_016224	chr6:-158364190
					AAATATTACTTATAATGTTTCAG	0.1	4MMs [2:7:15:19]	NR_015424	chr2:-89104853
					AAATATTACTTATAATGTTTCAG	0.1	4MMs [2:7:15:19]	NM_001164315	chr2:-97915841
					AAATATTACTTATAATGTTTCAG	0.1	4MMs [2:7:15:19]	NM_025190	chr2:+98123539
					AGACAACACTTATTTTGTATAAG	0.1	4MMs [4:6:7:14]	NM_001031748	chr12:+40115790
					AGATAAGAGTTAAATTTTATAGG	0.1	4MMs [6:9:13:17]	NM_024677	chr4:+40763400
					AGATATTTCTTATATAGTAGTGG	0.1	4MMs [7:8:16:20]	NM_006212	chr1:+207250341
					AGATTTCACTTATCTTCTATTAG	0.1	4MMs [5:7:14:17]	NM_001145418	chr22:+28692421
					AGATATTACTAATTTTGTACAAG	0	4MMs [7:11:14:20]	NM_198881	chrX:+106064190
					AGATATGGCTTATATTACTTAAG	0	4MMs [8:17:18:19]	NM_003183	chr2:-9663338
					AGATATTACTTATGATGAATTAG	0	4MMs [7:14:15:18]	NM_001007565	chr3:+100432614
**SEMA6A**	chr5:-115910803	94	GGCGGAGAAACTACGCGGTG**TGG**	44 (8 are in genes)	CACGGAAAATCTACGCGGTGGAG	0.9	4MMs [1:2:7:10]	NM_199141	chr19:+11022919
					GGCAGATGAACTACGCGGCGCAG	0.3	4MMs [4:7:8:19]	NM_002886	chr3:+152880965
					GGGGGAGAAGCCACGCGGGGGAG	0.2	4MMs [3:10:12:19]	NR_026961	chr21:-45230559
					GGCCGACAAACGACGCGGCGCGG	0.2	4MMs [4:7:12:19]	NM_177457	chr8:-143859255
					GGCGGAGCAGCTCCGCGTTGCGG	0.1	4MMs [8:10:13:18]	NM_001242376	chr17:+42989115
					GGAGGAGAAACTCCGAGGAGCAG	0	4MMs [3:13:16:19]	NM_015284	chr1:+43905617
					GGCGGAGAAACAAGGTGGTGGAG	0	3MMs [12:14:16]	NM_021819	chr15:-75115068
					GGCGGAGAAACTGCGCCCAGCAG	0	4MMs [13:17:18:19]	NM_006469	chr1:+185286312
	chr5:-115910724	92	TGGCCCCACCGCGCGCTAGG**CGG**	71 (11 are in genes)	TGGCCGCGCCGCGCGCGAGGTGG	0.7	3MMs [6:8:17]	NM_001037293	chr9:-112403040
					TGGCCCCGCGGCGCGCTCGGGGG	0.5	3MMs [8:10:18]	NM_001102562	chr5:+16179488
					TGGCCCCGCCCCGCCCTAGGGGG	0.4	3MMs [8:11:15]	NM_002013	chr14:-45603712
					GGGCCCCACGCCGCGCCAGGCGG	0.3	4MMs [1:10:11:17]	NM_198478	chr19:-45655730
					TGGCCACACTGGGCGCAAGGTGG	0.2	4MMs [6:10:12:17]	NR_036573	chr7:-148991673
					TGGCCCCGTCGGGCGCTAAGAAG	0.1	4MMs [8:9:12:19]	NR_037652	chr15:+80216144
					TCTCCCCACCGCGCGCCGGGAGG	0.1	4MMs [2:3:17:18]	NM_002209	chr16:+30485560
					TGGCTCCACGGCCCGCTCGGCGG	0.1	4MMs [5:10:13:18]	NM_018847	chr9:+21335193
					TGGCCCACCCGCCTGCTAGGAGG	0.1	4MMs [7:8:13:14]	NM_004996	chr16:-16177129
					TGGCCCCACAGGGCACTGGGGAG	0	4MMs [10:12:15:18]	NM_139317	chr20:+61869450
					TGGGCCCACCGCGCCCACGGGGG	0	4MMs [4:15:17:18]	NM_017723	chr9:+140175318
	chr5:+115910905	94	CGTAGAGCGTCCATCGCTTC**TGG**	22 (6 are in genes)	CTTGCAGCGTGCATCGCTTCAAG	0.8	4MMs [2:4:5:11]	NM_001012	chr1:+45244239
					CGGAGAGCGCGCCTCGCTTCCAG	0.3	4MMs [3:10:11:13]	NM_001007156	chr15:-88799681
					CGTGGAACGTGCAGCGCTTCAGG	0.1	4MMs [4:7:11:14]	NM_174873	chr12:-133197056
					CGTAAAGCCTCCATCCCTTTTGG	0.1	4MMs [5:9:16:20]	NM_052847	chr19:+2514741
					TGTAGAGCGCCCATGCCTTCCAG	0.1	4MMs [1:10:15:16]	NM_014339	chr22:-17585552
					CGGAGAGCGTCCTTCCCTGCCGG	0	4MMs [3:13:16:19]	NM_001252334	chr9:-130533686
	chr5:-115911046	80	AATAAAAATAGACGTAGTGC**AGG**	236 (10 are in genes)	CTTAAAAATACACGTAGTGTAAG	0.4	4MMs [1:2:11:20]	NM_001197223	chr5:+58271761
					AAGAAAACTAGAAGTAGTGGAGG	0.3	4MMs [3:8:13:20]	NM_002845	chr18:+8069698
					AATTAAAATAGACTTACTGCTGG	0.2	3MMs [4:14:17]	NM_005212	chr4:-71113557
					AATCAAACTAGACTTAGTTCAGG	0.1	4MMs [4:8:14:19]	NR_046535	chr13:+94806346
					AAAAAAAATATACTTAGTGGAAG	0.1	4MMs [3:11:14:20]	NM_015978	chr1:-75010140
					AACTAAAATAGACGTGGAGCAAG	0.1	4MMs [3:4:16:18]	NM_001195388	chr1:-157483718
					AATAAACACAGAGGTGGTGCTGG	0	4MMs [7:9:13:16]	NR_036636	chr12:+112337879
					AATAAAGATAGACTTAGTATGAG	0	4MMs [7:14:19:20]	NR_002174	chr6:+25081265
					AATAAAAATAAACCTAAGGCAAG	0	4MMs [11:14:17:18]	NM_016218	chr5:-74895501
					AATAAAAATAGACAAACTACCAG	0	4MMs [14:15:17:19]	NM_001004313	chr17:+10617170

MOUSE sgRNAs

Gene	Promoter location	sgRNAs score	sgRNA Sequence (5' to 3')	Total no. of offtarget sites (mm9)	off-target sites in exons (mm9)	score	mismatches	UCSC gene	locus
**Dclk1**	chromosome:GRCm38:3:55241764:55539668:1	91	GGCAAGTCCTTCTAGCGACT**GGG**	53 (7 are in genes)	AGCAAGTGCTTCTGGCGACTTAG	0.5	3MMs [1:8:14]	NM_012275	chr2:+113816645
					GTGAAGTCCTTCATGCGACTCAG	0.1	4MMs [2:3:13:14]	NM_004656	chr3:+52441991
					GGCAAGTGATTCCAGGGACTGAG	0.1	4MMs [8:9:13:16]	NM_139072	chr2:-230271921
					GGCATGTCCTTTTAGGAACTGGG	0	4MMs [5:12:16:17]	NM_181719	chr1:+20072874
					CGCAAGTGCTTCTGGGGACTGGG	0	4MMs [1:8:14:16]	NM_017671	chr20:+6090993
					GGCAAGTCATTGTAGGCACTTGG	0	4MMs [9:12:16:17]	NM_178822	chr3:-151161162
					GGCAAGTCCTTCAATGGGCTTAG	0	4MMs [13:15:16:18]	NR_047467	chr10:+92806676
		91	GATGAAGGCGACCTCAATAC**TGG**	47 (3 are in genes)	GTTGAAAGTGACCTCAATACCAG	1.1	3MMs [2:7:9]	NM_001818	chr10:+5258816
					TAGGAAGGCTACTTCAATACCAG	0.5	4MMs [1:3:10:13]	NM_032358	chr12:+539790
					GAGGAAGGAGACCTGAAGACAAG	0.1	4MMs [3:9:15:18]	NM_014663	chr1:+44133666
		97	CGCGAATGACGAGTGGAACG**TGG**	10 (1 is in gene)	GCGAGTGCCGGGTGGAACCGAG	0.1	4MMs [6:9:12:20]	NM_020493	chr17:-46692840
		91	GTTTTGAGGGGCAATCCTAC**TGG**	67 (7 are in genes)	GTCTTGAGCGGCAATCCTAACAG	0.9	3MMs [3:9:20]	NM_153774	chr1:+137300808
					GGCATGAGGGGCATTCCTACTGG	0.2	4MMs [2:3:4:14]	NM_007955	chr1:-137010643
					CTTTTGCCGGGCAGTCCTACGAG	0.2	4MMs [1:7:8:14]	NM_175556	chr4:+154365474
					GTTTTGGGGGACAATCCTGAAAG	0.1	4MMs [7:11:19:20]	NM_206935	chr5:-77436483
					GTTTTGAGGCACAATCTAACTGG	0.1	4MMs [10:11:17:18]	NM_207533	chr15:+95455038
					GTTTTGAGATGCAATGATACAAG	0.1	4MMs [9:10:16:17]	NM_027724	chr13:-38128622

						GTTTTGAGGGGCAGACCAGCGAG	0	4MMs [14:15:18:19]	NM_153125	chr2:-26264906
**Gap43**	GRCm38:16:42247842:42341251:-1		67	GGCAGTTAGCAGCAACTGTA**AGG**	203 (15 are in genes)	GGCAGTTAATAGCAACTGGAGGG	0.5	3MMs [9:10:19]	NM_021411	chr11:-115018195
						GTCAGCTTGCAGCAACTGTGCAG	0.4	4MMs [2:6:8:20]	NM_008087	chr7:+59152676
						GGCATCTCGCAACAACTGTAGAG	0.4	4MMs [5:6:8:12]	NM_172879	chr5:+3845264
						GGCCATTAGGAGCACCTGTAAGG	0.4	4MMs [4:5:10:15]	NM_001099631	chr4:-137811454
						GACATTTTGCAGCAACTCTAAGG	0.3	4MMs [2:5:8:18]	NM_009576	chr9:-91283726
						GGAAGTTTGCAGAAACTGTTCAG	0.3	4MMs [3:8:13:20]	NM_029570	chr3:+35725955
						GGCAAGTAGAAGCAGCTGTAGGG	0.2	4MMs [5:6:10:15]	NM_010305	chr5:-17779237
						GCCACTTAGAAGCTACTGTAAAG	0.2	4MMs [2:5:10:14]	NM_001168297	chr10:+11017752
						GGCAGTGAACAGCAAGTGTACAG	0.2	3MMs [7:9:16]	NM_001163270	chr17:+25388871
						GCCAATTAGCAGCAACTCTCAAG	0.1	4MMs [2:5:18:20]	NM_170690	chr10:-69342594
						GGCAGTGAGGAGTAGCTGTAGAG	0.1	4MMs [7:10:13:15]	NM_153054	chr8:-71562681
						TGCAGTTTGCAGCGCCTGTACAG	0.1	4MMs [1:8:14:15]	NM_001024910	chr10:+58639639
						GGTAGATAGCAGCAAATGAAAGG	0.1	4MMs [3:6:16:19]	NM_011159	chr16:-15769835
						GGCAGTTACCATAAAATGTAAGG	0	4MMs [9:12:13:16]	NM_181577	chr11:+28285825
			38	AAGAGGAGGAAGGGACGAGA**GGG**	501 (39 are in genes)	AAGAGATGGAAGGGACGAGAAAG	2.2	2MMs [6:7]	NM_007920	chr14:-79960522
						GGGCGGAGGAAGGGACGAGGGAG	0.7	4MMs [1:2:4:20]	NM_023587	chr16:-35022475
						GATAGGAGGTAGGGACCAGAGAG	0.6	4MMs [1:3:10:17]	NR_033577	chr15:+77450885
						CAGACGTGGAAAGGACGAGAGGG	0.5	4MMs [1:5:7:12]	NM_001145390	chr6:-142734457
						AAGACGAGTTGGGGACGAGATAG	0.4	4MMs [5:9:10:11]	NM_001100596	chr4:-42039230
						AAGACGAGTTGGGGACGAGATAG	0.4	4MMs [5:9:10:11]	NM_001099348	chr4:+42339327
						AAGACGAGTTGGGGACGAGATAG	0.4	4MMs [5:9:10:11]	NM_001100596	chr4:-42679198
						AGAAGGGGGAAGGGACTAGAAGG	0.4	4MMs [2:3:7:17]	NM_178897	chr5:-130738894
						CAGAAAAGGAAGGGACAAGAGGG	0.4	4MMs [1:5:6:17]	NM_001111143	chr3:+107021484
						AGAAGGAGGATGCGACGAGAGAG	0.3	4MMs [2:3:11:13]	NM_001146176	chr12:+78039496
						AAGGGGAGGGGGGGACAAGACGG	0.3	4MMs [4:10:11:17]	NM_010902	chr2:+75542808
						AAGAAGAGGTTGGGACAAGATGG	0.3	4MMs [5:10:11:17]	NR_046048	chr9:-47608999
						TACAGGATGAAGGGAAGAGAGAG	0.3	4MMs [1:3:8:16]	NM_021557	chr12:+80292732
						GTCAGGAGGAAGGGAGGAGACAG	0.3	4MMs [1:2:3:16]	NM_009032	chr19:+4785282
						GATAGGAAGAAGGGAAGAGAGGG	0.3	4MMs [1:3:8:16]	NM_001114179	chrX:+133527464
						CTGAGGAGGGAGGGAAGAGACAG	0.3	4MMs [1:2:10:16]	NM_001170433	chr6:-146941991
						AAGACGTGTAAGGGACAAGATGG	0.2	4MMs [5:7:9:17]	NM_010498	chrX:-67613300
						AAGGTGAAGAAGGCACGAGAGAG	0.2	4MMs [4:5:8:14]	NM_001195025	chr1:+134213012
						ATAAGGAGCAAGGGACGGGACAG	0.2	4MMs [2:3:9:18]	NM_172945	chr11:+77284783
						AGGAAGAGGAAGGGTCGAGTGAG	0.2	4MMs [2:5:15:20]	NM_172339	chr2:-26232531
						TAGAGGAGTAATGGACCAGATGG	0.2	4MMs [1:9:12:17]	NM_001163996	chr5:+110558845
						AACATGAGAAAGGGATGAGATGG	0.2	4MMs [3:5:9:16]	NM_026422	chr2:-35997190
						GGGAGGAGGAGGGGAGGAGAGGG	0.2	4MMs [1:2:11:16]	NM_001146307	chr4:+140953825
						AGGAGGAGGAGGAGACGAGGCAG	0.1	4MMs [2:11:13:20]	NM_001081193	chr10:-120369007
						AAGAGGCAGAAGAGACAAGAAGG	0.1	4MMs [7:8:13:17]	NM_138944	chr8:+80958559
						AACAGGAGAGAGGGAGGAGAGAG	0.1	4MMs [3:9:10:16]	NM_146283	chr13:+21629258
						AACAGGAGAGAGGGAAGAGAGGG	0.1	4MMs [3:9:10:16]	NM_177767	chr8:-96591501
						GAGAGGAGGGTGGGATGAGAAGG	0.1	4MMs [1:10:11:16]	NM_021505	chr5:+123241770
						AAGAGGAAAGAGGGAGGAGAAGG	0.1	4MMs [8:9:10:16]	NM_011602	chr4:-43553320
						AAGGAGAGGAAGGGACGTGGCAG	0.1	4MMs [4:5:18:20]	NM_028096	chr1:-37668626
						AAGAGGAGCAAGGGATGAGGGAG	0.1	3MMs [9:16:20]	NM_001081132	chr2:+5977080
						CAGATGAGGAAGGGACCTGAGGG	0.1	4MMs [1:5:17:18]	NM_027248	chr14:+52628846
						AGGAGGAAGAAGGGAAGAGCTGG	0.1	4MMs [2:8:16:20]	NM_008876	chr11:-70368769
						AAGAAGAGAAAGAGACGACAGAG	0.1	4MMs [5:9:13:19]	NM_029741	chr7:-52605626
						AAGAGTGGGAAAGGACCAGAGGG	0.1	4MMs [6:7:12:17]	NM_133674	chr6:+43233363
						CAGAGCTGGAAGGGAGGAGAGAG	0.1	4MMs [1:6:7:16]	NM_001012322	chr1:-121903510
						TAGTGGAGGAACGAACGAGACAG	0.1	4MMs [1:4:12:14]	NM_001012625	chr1:-22369585
						AAGGGGAGGAAGGGATGGGAGAG	0.1	3MMs [4:16:18]	NM_008192	chr11:+69032480
						CAGATGAGGAAGAGAGGAGAAAG	0.1	4MMs [1:5:13:16]	NM_020002	chr14:+56243031
	chr16:+42341184		50	AATTCTGAGAAAGGGACCCA**GGG**	430 (19 are in genes)	GCTTCAGAGAAAGGGACCCACGG	1.5	3MMs [1:2:6]	NM_027292	chr17:-48590231
						AGCTCTGAGAAAGGGAACCAGAG	1.2	3MMs [2:3:17]	NM_007687	chr19:-5492831
						TATTATGAAGAAGGGACCCATGG	0.8	4MMs [1:5:9:10]	NM_026886	chr5:-116925274
						ATCTGGGAGAAAGGGACCCACAG	0.8	4MMs [2:3:5:6]	NM_010626	chr7:+86844986
						CATCTTGAGAAAGGGACCCTGAG	0.7	4MMs [1:4:5:20]	NM_025360	chr9:-89594205
						AAAGCAGAGAGAGGGACCCAGGG	0.5	4MMs [3:4:6:11]	NM_011201	chr2:-167790426
						AAATAAGAGAAAGGGACCCGTGG	0.4	4MMs [3:5:6:20]	NM_009154	chr15:-32621428
						GTTTCTGACAAAGGGAACCACAG	0.4	4MMs [1:2:9:17]	NM_001177571	chr10:+41289604
						CATTGTGAGATAGGGACCAAGAG	0.3	4MMs [1:5:11:19]	NM_145394	chr3:-121162875
						GCTTCTGAGAAAGGAAGCCAAAG	0.2	4MMs [1:2:15:17]	NM_010382	chr17:-34451964
						AAATCTGACAATGGGACCTACAG	0.2	4MMs [3:9:12:19]	NM_025655	chr11:+76723744
						AAGTCTGAGGAAGGGAAGCAGAG	0.1	4MMs [3:10:17:18]	NM_030035	chr16:+36919767
						AATACTGAGAGAGGGAGCGAAAG	0.1	4MMs [4:11:17:19]	NM_010427	chr5:-16099893
						AACTCTGAAAGAGGGACTCAAAG	0.1	4MMs [3:9:11:18]	NM_001164220	chr14:-62223790
						AATGCTGGGAAAAAGACCCAAAG	0.1	4MMs [4:8:13:14]	NM_021380	chr1:+132804998
						CATTCTTAGAAATGGGCCCATAG	0.1	4MMs [1:7:13:16]	NM_178703	chr6:-114252681
						AATTCTGACAATAGTACCCAAGG	0	4MMs [9:12:13:15]	NM_001039546	chr9:-80156942
						GATTCTGAGAAAGAGAGCAAGAG	0	4MMs [1:14:17:19]	NM_153145	chr11:+109933195
						AACTCTGAGAAAGAGTCCCGTGG	0	4MMs [3:14:16:20]	NM_178759	chr11:+46629293
	chr16:-42341007		38	GCTATGGGGTGGGGGTAGAA**AGG**	500 (42 are in genes)	CCTGAGGTGTGGGGGTAGAAAAG	1.4	4MMs [1:4:5:8]	NM_029964	chr11:+117668477
						AGAAAGGGGTGGGGGTAGAAGAG	1.3	4MMs [1:2:3:5]	NM_016765	chr17:-35197006
						GCTTTGGGGAGGGGGTAGGAGAG	0.9	3MMs [4:10:19]	NM_008269	chr11:-96162656
						GGTTTGGGGCTGGGGTAGAAGAG	0.7	4MMs [2:4:10:11]	NM_175184	chr2:+33680573
						GCTGGGGAGTGGGGGTAGAGAGG	0.7	4MMs [4:5:8:20]	NM_016674	chr16:+26371409
						GATATGGGGTGGGGGTGGGAAGG	0.4	3MMs [2:17:19]	NM_001033124	chr3:+87585874
						GCTATGGGTTAGGGGTGGAAGGG	0.3	3MMs [9:11:17]	NM_008063	chr9:-44207630
						GCTTTGGAGTTGTGGTAGAATGG	0.3	4MMs [4:8:11:13]	NM_019995	chr3:+54502607
						GATATGGGGTGGGGCTAGCAAAG	0.3	3MMs [2:15:19]	NM_001253887	chr7:-112717079
						TCTTGGGGGTGGGGGGAGAAAAG	0.3	4MMs [1:4:5:16]	NM_173423	chr18:+46684585
						ACCCTGGGGTGGGGGGAGAAGGG	0.3	4MMs [1:3:4:16]	NM_001130459	chr11:-3831345
						GGTGGGGGGTGGGGGGAGAAGGG	0.3	4MMs [2:4:5:16]	NM_153137	chr1:-195023809
						GTTGGGGGGTGGGGGGAGAAGAG	0.3	4MMs [2:4:5:16]	NM_008453	chr5:-65221410
						GGGGTGGGGTGGGGGGAGAAGGG	0.3	4MMs [2:3:4:16]	NM_010927	chr11:-78734566
						GCTTGGGGGGGGGGGGAGAAGGG	0.2	4MMs [4:5:10:16]	NM_011467	chr6:+85084044
						GCTATGGGATGGGGGTAGGGTGG	0.2	3MMs [9:19:20]	NM_080470	chr15:-84943022
						TCTCTGGGGTGGGGGTTGCAAAG	0.2	4MMs [1:4:17:19]	NM_178379	chr11:-63886969
						GCTGTTGGGTGTGGGTAGACGGG	0.2	4MMs [4:6:12:20]	NM_177129	chr1:-134425439
						GTTCTGGGGTGGGGGTGGGAGGG	0.2	4MMs [2:4:17:19]	NM_028043	chr1:+39593218
						GTTAGGGGGTGGGGGTGGCAGAG	0.2	4MMs [2:5:17:19]	NM_022428	chr8:+95198986
						GTGATGGGGTGGGGGTTGGAGGG	0.2	4MMs [2:3:17:19]	NM_021042	chr6:-142621227
						GCAGTGGGGTGGGGGTGGGAGGG	0.2	4MMs [3:4:17:19]	NR_045283	chr15:+37351701
						GATGTAGGGTGGGGGTACAAAGG	0.2	4MMs [2:4:6:18]	NM_008065	chr16:+84835437
						GCTATGGAATGAGGGTAGAGAAG	0.2	4MMs [8:9:12:20]	NR_045332	chr9:+98649856
						GCACTGAGGTGGGGGCAGAAGGG	0.2	4MMs [3:4:7:16]	NM_022888	chr9:+14706276
						CCTGTGGGGTGGGGGGAGAGGAG	0.1	4MMs [1:4:16:20]	NR_045873	chr11:+78837043
						GCATTGGGGTGGGGGTGTAAGAG	0.1	4MMs [3:4:17:18]	NM_177352	chr9:+96051225
						GCTAGTGGGTGGGGCTAGATGGG	0.1	4MMs [5:6:15:20]	NM_007494	chr2:-31365860
						ACTAGGGGGTGGGGGGAGCAGGG	0.1	4MMs [1:5:16:19]	NM_172487	chr6:+113451604
						GGGATGGGGTGGGGGCAGTAGGG	0.1	4MMs [2:3:16:19]	NM_016753	chr3:+67267448
						GCTCTGAGGTGAGGGAAGAACAG	0.1	4MMs [4:7:12:16]	NM_028940	chr4:-9208713
						GGTATGGGGTGGGGGTGGGGAAG	0.1	4MMs [2:17:19:20]	NM_009697	chr7:+77498800
						GGTATGGGGGGGGGGCAGCACAG	0.1	4MMs [2:10:16:19]	NM_018813	chr12:-21318290
						GCTTTGGGGTGGGGGTGGTGTGG	0.1	4MMs [4:17:19:20]	NR_003568	chr13:+12710209
						GCTCTGGGGAGGGAGTCGAAGAG	0.1	4MMs [4:10:14:17]	NM_001199733	chr17:+34046903
						GCGATGTGGTGGGGGTGTAAGGG	0.1	4MMs [3:7:17:18]	NM_001003953	chr5:-123331117
						GCTACGGGGTGGGGGTGGGGAGG	0.1	4MMs [5:17:19:20]	NM_172887	chr5:-151293626
						GCTATGGGGGGTGGGTGGGAGAG	0.1	4MMs [10:12:17:19]	NM_027144	chr9:-42776273
						GCTATAGGTTGGGGGTAGGTGGG	0.1	4MMs [6:9:19:20]	NM_008667	chr1:-52523674
						GCCATAGGGTAGGTGTAGAAAGG	0.1	4MMs [3:6:11:14]	NM_175644	chr12:+76513870
						GATATGGGGTGGGGTTTGAGGGG	0.1	4MMs [2:15:17:20]	NM_001190703	chr12:-110699597
						GCTCTGGGTTGGGGGTTAAAAAG	0.1	4MMs [4:9:17:18]	NM_019640	chr5:-111816965
**Sema6a**	chr18:-47528597		93	TGGCGTCTCCTCCGACCCGA**AGG**	45 (8 are in genes)	TGGCGGCTCCTCCGACCCGGGAG	3.1	2MMs [6:20]	NM_008429	chr1:+174256637
						CAGCGGCTCCTCCGCCCCGAGGG	0.3	4MMs [1:2:6:15]	NM_030563	chr8:+89408776
						TGGCGTCTGCACCGGCCCGAAGG	0.2	3MMs [9:11:15]	NM_020605	chr8:+124254601

						TGGGGGCTGCTCAGACCCGAAGG	0.2	4MMs [4:6:9:13]	NM_177733	chr4:-135728775
						CGGCGTCTCCTCCGCCCTGAAGG	0.2	3MMs [1:15:18]	NM_027295	chr5:+42099324
						TGGCATCTCCTCAGACCTGGAAG	0.1	4MMs [5:13:18:20]	NM_011941	chr2:+119848102
						CGGCGGCTCCTCCCACCCAAGGG	0	4MMs [1:6:14:19]	NM_027496	chr15:-31297336
						TGGCTCCTCCTCCCACCCTATGG	0	4MMs [5:6:14:19]	NM_177078	chr5:+113341910
	chr18:+47528713		92	CAGGAGTAGTGTGCCGCACG**GGG**	53 (8 are in genes)	CATGTGCAGTGTGCCGCCCGTGG	0.2	4MMs [3:5:7:18]	NM_146019	chr11:+69171334
						CTGGAGTAGTGGGCCCCACTGAG	0.1	4MMs [2:12:16:20]	NM_010227	chrX:-71483298
						CAGGCGTTGTGTGGCACACGTGG	0	4MMs [5:8:14:16]	NM_010491	chr6:-142251862
						CAGGAGAAGTGTGCAGCAGCTGG	0	4MMs [7:15:19:20]	NM_001123370	chr17:+15117236
						CAGGAGAAGTGTGCAGCAGCTGG	0	4MMs [7:15:19:20]	NM_001123372	chr17:+15148608
						CAGGAGAAGTGTGCAGCAGCTGG	0	4MMs [7:15:19:20]	NM_001039552	chr17:+15179983
						CAGGAGTAGTGTGAAGGACCGGG	0	4MMs [14:15:17:20]	NM_001037298	chr8:-125010442
	chr18:+47528885		95	TCTCCTAGAGTCGCCCCCGC**TGG**	44 (12 are in genes)	CCTCCGGGAGCCGCCCCCGCGGG	0.3	4MMs [1:6:7:11]	NM_001270646	chr18:-35812234
						GCTCCCAGAGTGGCCCGCGCGGG	0.2	4MMs [1:6:12:17]	NM_016974	chr7:-52962339
						TCTCCCAGACCCGCCCCCTCCGG	0.1	4MMs [6:10:11:19]	NM_138589	chr7:+129210764
						TAACCTAGAGTCGCCCTTGCAGG	0.1	4MMs [2:3:17:18]	NM_027389	chr3:+88711270
						TCTCCTCTAGTCGGCCTCGCCGG	0.1	4MMs [7:8:14:17]	NM_027215	chr7:+31514486
						TCTCCTTTAGTCTTCCCCGCTGG	0.1	4MMs [7:8:13:14]	NM_008604	chr3:+63165842
						TCTCCTCGAGCAGCCCCAGCAGG	0.1	4MMs [7:11:12:18]	NM_010856	chr14:+55575434
						TCTCCCAGAGCCTCTCCCGCCAG	0	4MMs [6:11:13:15]	NM_146260	chr9:-110782556
						TCTCATAGAGTCCCGCCAGCGAG	0	4MMs [5:13:15:18]	NM_080641	chr2:+180510702
						TCACCTAGAGTCAACCCCACAGG	0	4MMs [3:13:14:19]	NM_138306	chr2:+91782897
						TCTCCTAGAGACTCTCCAGCCAG	0	4MMs [11:13:15:18]	NM_177809	chr13:+56544269
						TCTCCTAGTGTCACCTCTGCCAG	0	4MMs [9:13:16:18]	NM_008131	chr1:+155756516
	chr18:+47528787		61	CAGGGCTCAGTAATGCTGTC**TGG**	274 (26 are in genes)	CATGCCTCAGTAATGCTGTTCAG	1.4	3MMs [3:5:20]	NM_026482	chr10:+98472314
						CAGGTCTCATTATTGCTGTCAGG	1	3MMs [5:10:13]	NM_138677	chr6:-108794840
						CAACCCTCAGGAATGCTGTCAAG	0.8	4MMs [3:4:5:11]	NM_001165984	chr3:-89807069
						CAGTGTTCAGTAATGCAGTCTGG	0.7	3MMs [4:6:17]	NM_001081354	chrX:+68407403
						CAAGGCCTAGTCATGCTGTCGGG	0.5	4MMs [3:7:8:12]	NM_009190	chr1:+108676778
						CAGTGCTGAGAGATGCTGTCAGG	0.4	4MMs [4:8:11:12]	NM_027258	chr11:-116199115
						CAGAGCTCATGCATGCTGTCAAG	0.4	4MMs [4:10:11:12]	NM_130884	chr2:-130109681
						CTGGGCTCTGAGATGCTGTCAGG	0.2	4MMs [2:9:11:12]	NM_001195816	chr1:-177914509
						GAGGGCACAGGAATGCTGGCTGG	0.2	4MMs [1:7:11:19]	NM_172892	chr6:+35221819
						CAGGGCTCAGCACTGCTGGCTGG	0.2	3MMs [11:13:19]	NM_001164258	chr7:+107064352
						CAGGACTCAGGGATGCTGTGGGG	0.2	4MMs [5:11:12:20]	NM_177611	chr2:+24263264
						CAGGACTGAGTCATGGTGTCCAG	0.1	4MMs [5:8:12:16]	NM_033324	chr16:-18258518
						CGGTGCTCAGGAAGGCTGTCAGG	0.1	4MMs [2:4:11:14]	NM_001163721	chr4:+153399631
						CAGGGCTAAGTTCTGCTGTGGGG	0.1	4MMs [8:12:13:20]	NM_133351	chr7:-135071529
						CAGGCCTCAGCAATGCTGCAAGG	0.1	4MMs [5:11:19:20]	NM_146261	chrX:-133611782
						CAGGGCAGAGTGATGTTGTCAGG	0.1	4MMs [7:8:12:16]	NM_026960	chr15:+75694725
						CAGGGCTCAGCATTGCCGACCAG	0	4MMs [11:13:17:19]	NM_001042727	chr15:-102070320
						CAGGGCTCTGGAACGCTGTGTGG	0	4MMs [9:11:14:20]	NR_045159	chr4:-139499454
						CAGGGCTCAGTGTTCCTGTGTGG	0	4MMs [12:13:15:20]	NM_145532	chr2:-127530847
						CAGGCCTCAGAAAAGCTTTCTGG	0	4MMs [5:11:14:18]	NR_029880	chr12:+110947179
						CAGGGCCCAGTAAAGCTGGGAGG	0	4MMs [7:14:19:20]	NM_009025	chr8:-13578554
						CAGGGCTCGGTAGTACTCTCAGG	0	4MMs [9:13:15:18]	NM_001253690	chr11:-3190474
						CAGGGCTCAGTGATGGAGACTGG	0	4MMs [12:16:17:19]	NM_026644	chr17:+12412007
						CAGGGCTCAGAAATGTGTTCCAG	0	4MMs [11:16:17:18]	NM_212445	chr9:+53198923
						CAGGGCTCAGTACTGGTTTGTGG	0	4MMs [13:16:18:20]	NM_138652	chr14:+57006472
						CAGGGCTCAGTACATGTGTCTGG	0	4MMs [13:14:15:16]	NM_011708	chr6:+125504583

Supplementary Table 6. Primer and oligonucleotide sequences.

Primers for cloning		
Primer Name	Forward or Reverse	Primer sequence (5'-3')
Cjk-SA1	Reverse	CATTTCTATCAACTAGGAGCTG
Cjk-SA3	Reverse	ACGCGTCGACATGTCTTCCCTTCCTGGGTGCA
Cjk-SA4	Reverse	ATAGTTTAGCGGCCGCCTAAAGAAGACGTCCTCTGCATTC
Cjk24	Forward	ACGCGTCGACATGATGGATCCATGTTCAGTTG
Cjk25	Reverse	ATAGTTTAGCGGCCGCTTATCCAGCATGTTTATTATGAAT

RT-qPCR (mouse)		
Primer Name	Forward (5'-3')	Reverse (5'-3')
C11orf46	GACCGAAAGTGGCTGTATG	ATATGGGTGACAGGGAGATAA
Dclk1	TCCACCGGAATTGAACTCGG	GGGAGCGAACAGTCTCAGA
Sema6a	ACAGCCTGCCCCCTAAAGT	AGCTCCTCTTATATTCGAGCCC
Plxna4	ACAGGGCACATTTATTTGGGG	CACTTGGGGTTGTCCTCATCT
Nuak1	CAGGCCGAGTGGTTGCTATAA	TGGTTGAGGGATGACATGATCT
Gap43	TGGTGTCAAGCCGGAAGATAA	GCTGGTGCATCACCCTTCT
Hprt1	TCAGTCAACGGGGGACATAAA	GGGGCTGTACTGCTTAACCAG

sgRNA protospacer sequences		
Region of Interest	Oligonucleotide Name	protospacer sequence (5'-3')
mouse *Dclk1*	mDclk1-A	AGTCGCTAGAAGGACTTGCC
	mDclk1-D	GTAGGATTGCCCCTCAAAAC
mouse *Sema6a*	mSema6a-A	TCGGGTCGGAGGAGACGCCAC
	mSema6a-D	GACAGCATTACTGAGCCCTGC

ChIP-qPCR (mouse)		
Primer Name	Forward (5'-3')	Reverse (5'-3')
Dclk1	TTTGAGGGGCAATCCTACTG	GCCTTCATCAAAGGAGACCA
Sema6a	AGAGGGAGGCAGAGAGTGTG	GGCTGGGCTTCTCCTAGAGT

	Primer	Sequence 5' - 3'	Species	Size
qRT-PCR	Primer bank ID			
**Human primers:**				
C11orf46 (NM_152316)	22748692c1-forward	TGGAGTCCAGCTTCGTACTAC	human	140bp
	22748692c1-reverse	TGTTCCCAGAAAGTGTCATTCC	human	
SETDB1 (NM_001145415)	224177466c3-forward	TAAGACTTGGCACAAAGGCAC	human	104bp
	224177466c3-reverse	TCCCCGACAGTAGACTCTTTC	human	
ATF7IP (MCAF1) (NM_018179)	38261961c1-forward	AAGGCTCGAAAAACGATGAGAG	human	102bp
	38261961c1-revers	GCCATTTAACAGCTTGACATCAG	human	
TRIM28(KAP1) (NM_005762)	14971416c3-forward	CGGGATGGTGAACGTACTGTC	human	93bp
	14971416c3-reverse	GTCTCGGCAGGTGAGAGTATC	human	
HPRT1 (NM_000194)	164518913c1-forward	CCTGGCGTCGTGATTAGTGAT	human	131bp
	164518913c1-reverse	AGACGTTCAGTCCTGTCCATAA	human	
beta-Actin (NM_001101)	4501885a1-forward	CATGTACGTTGCTATCCAGGC	human	250bp
	4501885a1-reverse	CTCCTTAATGTCACGCACGAT	human	
**Mouse primers:**				
C11orf46 (NM_173750)	68299772c3-forward	CAATGGAAGTGTGGACGTTGA	mouse	103bp
	68299772c3-reverse	TGGGTTTGTAAACTGTAGTGACC	mouse	
Hprt (NM_013556)	7305155a1-forward	TCAGTCAACGGGGGACATAAA	mouse	142bp
	7305155a1-reverse	GGGGCTGTACTGCTTAACCAG	mouse	
beta Actin (NM_007393)	6671509a1-forward	GGCTGTATTCCCCTCCATCG	mouse	154 bp
	6671509a1-reverse	CCAGTTGGTAACAATGCCATGT	mouse	
Ctnnd2	112363089c1-forward	ATGGCTCTGAGACGGAAACC	mouse	112 bp
	112363089c1-reverse	CTGGCTACGATCTGGCGTTC		
Dclk1	26328245a1-forward	TCCACCGGAATTGAACTCGG	mouse	65 bp
	26328245a1-reverse	GGGAGCGAACAGTCTCAGA		
Enc1	31560703a1-forward	CTGTTTCATAAGTCCTCCTACGC	mouse	169
	31560703a1-reverse	CACCACTGAACATGGCTTCG		
Map2	Map2-forward	GGAAAGATGAAGGAAAGGCACCAC	mouse	344
	Map2-reverse	GGCTGGTCCTTGTATTGGGCTTCC		
Nuak1	52138543c3-forward	CAGGCCGAGTGGTTGCTATAA	mouse	
	52138543c3-reverse	TGGTTGAGGGATGACATGATCT		
Plxna4	28461143a1-forward	ACAGGGCACATTTATTTGGGG		
	28461143a1-reverse	CACTTGGGGTTGTCCTCATCT	mouse	107
Ptn	12857177a1-forward	CTCTGCACAATGCTGACTGTC		
	12857177a1-reverse	ACAGCTTCTTACCTTGAGGCTT		
Sema6a	11093909a1-forward	ACAGCCTGCCCCCTAAAGT	mouse	135
	11093909a1-reverse	AGCTCCTCTTATATTCGAGCCC		
shc3	6677937a1-forward	GCCGGGAGTCACCTATGTG	mouse	100
	6677937a1-reverse	TTCCCTGGTAACCTGAGTTCTT		
Slc8a3	30851385a1-forward	GTGAACCGAAATGGATGGAACG	mouse	112
	30851385a1-reverse	TCACCCAATACTGGCTTTCCC		
Syt1	356640230c3-forward	GACAAAAGTCCACCGGAAAACC	mouse	92
	356640230c3-reverse	CCAGTGTCTTGCCACCTAATTC		
Cdk5r1	6753384a1-forward	CTGTCCCTATCCCCCAGCTAT	mouse	140
	6753384a1-reverse	GGCAGCACCGAGATGATGG		
DCLK1	4758128a2-forward	GCTGATTTGACCCGAACTCTG	Human	133
	4758128a2-reverse	AGCCACATACATAACTCTCTCCT		
SEMA6A	145553983c2-forward	ACATTGCTGCTAGGGACCATA	Human	119
	145553983c2-reverse	TCTGCATGTGTCTACATCGGC		
GAP43	194248055c1-forward	GGCCGCAACCAAAATTCAGG	Human	167
	194248055c1-reverse	CGGCAGTAGTGGTGCCTTC		
PLXNA4	157738644c1-forward	GTCATTTGTCACATTCCGAGGA	Human	107
	157738644c1-reverse	GCTTGTAAATCCGATTGACGGC		
NUAK1	48374438c3-forward	ATATACTGCTCGATGACAACTGC	Human	108
	48374438c3-reverse	CATAGAGTGGACTCCCACAAAAC		
NDRG1	207028746c1-forward	CTCCTGCAAGAGTTTGATGTCC	Human	127
	207028746c1-reverse	TCATGCCGATGTCATGGTAGG		
