# Supplementary material for: In vivo epigenetic editing of Sema6a promoter reverses transcallosal dysconnectivity caused by C11orf46/Arl14ep risk gene
Source: Nat Commun. 2019 Sep 11;10:4112. doi: 10.1038/s41467-019-12013-y (PMC6739341; doi:10.1038/s41467-019-12013-y)
Supplement: Supplementary file 3 — Reporting Summary [file 41467_2019_12013_MOESM3_ESM.pdf]

nature research

Corresponding author(s): Atsushi Kamiya, Schahram Akbarian

Last updated by author(s): May 16, 2019

Reporting Summary

Nature Research wishes to improve the reproducibility of the work that we publish. This form provides structure for consistency and transparency in reporting. For further information on Nature Research policies, see Authors & Referees and the Editorial Policy Checklist .

Statistics

For all statistical analyses, confirm that the following items are present in the figure legend, table legend, main text, or Methods section.

n/a	Confirmed	
☐	☒	The exact sample size (n) for each experimental group/condition, given as a discrete number and unit of measurement
☐	☒	A statement on whether measurements were taken from distinct samples or whether the same sample was measured repeatedly
☐	☒	The statistical test(s) used AND whether they are one- or two-sided<br>*Only common tests should be described solely by name; describe more complex techniques in the Methods section.*
☐	☒	A description of all covariates tested
☐	☒	A description of any assumptions or corrections, such as tests of normality and adjustment for multiple comparisons
☐	☒	A full description of the statistical parameters including central tendency (e.g. means) or other basic estimates (e.g. regression coefficient) AND variation (e.g. standard deviation) or associated estimates of uncertainty (e.g. confidence intervals)
☐	☒	For null hypothesis testing, the test statistic (e.g. F, t, r) with confidence intervals, effect sizes, degrees of freedom and P value noted<br>*Give P values as exact values whenever suitable.*
☒	☐	For Bayesian analysis, information on the choice of priors and Markov chain Monte Carlo settings
☒	☐	For hierarchical and complex designs, identification of the appropriate level for tests and full reporting of outcomes
☒	☐	Estimates of effect sizes (e.g. Cohen's d, Pearson's r), indicating how they were calculated

Our web collection on statistics for biologists contains articles on many of the points above.

Software and code

Policy information about availability of computer code

Data collection	3.0 T Philips Achieva MRI scanner with an 8-channel phased array head coil (Human MRI data), ImageJ (NIH), Zen (Zeiss), LSM browser (Zeiss), MS Excel, Adobe Photoshop, Adobe Illustrator
Data analysis	FreeSurfer's (version 5.3), SPSS Statistics (IBM, version 24), Prism8 (GraphPad), MS Excel

For manuscripts utilizing custom algorithms or software that are central to the research but not yet described in published literature, software must be made available to editors/reviewers. We strongly encourage code deposition in a community repository (e.g. GitHub). See the Nature Research guidelines for submitting code & software for further information.

Data

Policy information about availability of data

All manuscripts must include a data availability statement. This statement should provide the following information, where applicable:

- Accession codes, unique identifiers, or web links for publicly available datasets
- A list of figures that have associated raw data
- A description of any restrictions on data availability

Raw data of RNA-seq is available at Gene Expression Omnibus (GEO). Accession number is GSE119360.   .

Field-specific reporting

Please select the one below that is the best fit for your research. If you are not sure, read the appropriate sections before making your selection.

☒ Life sciences ☐ Behavioural & social sciences ☐ Ecological, evolutionary & environmental sciences

Life sciences study design

All studies must disclose on these points even when the disclosure is negative.

Sample size	For Figure 1b, a total of 62 patients were included in the analysis. Required sample size in the other studies was calculated based on pilot result. We set 1-β=o.80, α=0.05.
Data exclusions	No data were excluded.
Replication	Two persons analyzed separately and confirmed to get the result with same tendency.
Randomization	Samples were not randomized.
Blinding	Collected data were analyzed with blinded manner by persons who did not collect data and did not know which sample belongs to which group.

Reporting for specific materials, systems and methods

We require information from authors about some types of materials, experimental systems and methods used in many studies. Here, indicate whether each material, system or method listed is relevant to your study. If you are not sure if a list item applies to your research, read the appropriate section before selecting a response.

Materials & experimental systems

n/a	Involved in the study	
☐	☒	Antibodies
☐	☒	Eukaryotic cell lines
☒	☐	Palaeontology
☐	☒	Animals and other organisms
☐	☒	Human research participants
☐	☒	Clinical data

Methods

n/a	Involved in the study	
☒	☐	ChIP-seq
☐	☒	Flow cytometry
☐	☒	MRI-based neuroimaging

Antibodies

Antibodies used	Nestin antibody (Becton Dickinson#556309), Pax6 (Biolegend #901301), Tbr2 (Abcam# ab23345), Tbr1 (Abcam#ab31940), NeuroD2 (Abcam#ab109406), SATB2 (Santa Cruz#sc-81376), CUX1 (Santa Cruz #sc-13024), CTIP2 (abcam #ab18465), CaMKII (Millipore #05-532), Olig-2 (Millipore AB9610), ALDH1L1 (abcam ab177463), Iba-1 (Wako #019-19741), GFP (Nacalai #GF90R), H3K9me3 (abcam #ab8898), Affinity purified Guinea pig Anti-C11orf46, and Mouse Anti-C11orf46 monoclonal.
Validation	Affinity purified Guinea pig Anti-C11orf46 was validated by western blot analysis using purified recombinant full length C11orf46 protein (Supplementary Fig. 1c). Mouse monoclonal Anti-C11orf46 was validated by western blot analysis using purified recombinant full length C11orf46 protein (Supplemental Fig. 4f). Information regarding commercial antibodies are available at vendors site, can be accessed using the product catalog numbers.

Eukaryotic cell lines

Policy information about cell lines

Cell line source(s)	NSC34 cells (Androphy lab, Indiana university school of medicine), Patient derived lymphoblastoid cells are provided by Dr. Joan C. Han, Flp-In™ T-REx™ 293 Cell Line (Thermo scientific # R78007) and HEK293 cells are obtained from ATCC.
Authentication	NSC34 cells are previously described, Flp-In T-REx 293 cells are validated by vendor, lymphoblastoid cells are validated for chromasomal abnormalities previously.
Mycoplasma contamination	No signs of mycoplasma contamination, thus not treated with anti mycoplasma compounds.
Commonly misidentified lines (See ICLAC register)	*Name any commonly misidentified cell lines used in the study and provide a rationale for their use.*

Animals and other organisms

Policy information about studies involving animals; ARRIVE guidelines recommended for reporting animal research

Laboratory animals	Male and female C57BL/6J (The Jackson Laboratory: #000664) were maintained in our animal facility for animal studies. Brain samples were collected at the age from embryonic day 15 (E15) to postnatal day 14 (P14).
Wild animals	The study did not involve wild animals.
Field-collected samples	The study did not involve field-collected samples.
Ethics oversight	All experiments were performed in accordance with the institutional guidelines for animal experiments of Johns Hopkins University.

Note that full information on the approval of the study protocol must also be provided in the manuscript.

Human research participants

Policy information about studies involving human research participants

Population characteristics	Aged 6-54 years (mean=19, SD=12), 45% male, 23 healthy controls, 12 isolated PAX6 haploinsufficiency, 27 with chromosome 11p13-14 region heterozygous deletion including PAX6 and C11orf46
Recruitment	Patients who had prior genetic testing confirming diagnosis of WAGR/11p13 deletion syndrome or isolated aniridia with known PAX6 mutation or deletion, as well healthy control subjects who had no chronic medical conditions were recruited through local advertisements and on-line postings. Because recruitment of WAGR syndrome patients was on the basis of having aniridia, all participants were, by definition, PAX6 haploinsufficient, and, therefore, we were unable to assess the effect of C11orf46+/- independent of PAX6+/- and the phenotype of isolated C11orf46+/- is unknown (although homozygous mutations in C11orf46 have been reported in association with intellectual disability). PAX6 is a transcription factor regulating neurogenesis and rostrocaudal patterning during development. PAX6 haploinsufficiency is associated with corpus callosum hypoplasia in both rodents and humans. Thus, there remains the possibility that additional loss of PAX6+/- may be required for C11orf46+/- to cause significant defects in morphologic brain development. However, we were able to demonstrate that patients with combined loss of both genes had more severely reduced corpus callosum volumes compared to isolated PAX6+/-, confirming an additional role of C11orf46 in neurodevelopment. Whether the impact of mutations in the above molecules on axonal development is additive or synergistic remains to be determined. In addition, because the WAGR CNV encompasses a large genomic regions that encompass many genes, hampering identification of genetic drivers responsible for specific phenotypes shown in CNVs-associated disease conditions, our data do not exclude the possibility that other genes in 11p13 deletion region, besides BDNF, PAX6, and C11orf46 may have independent effect on axonal and other anatomical phenotypes as well as behavioral outcomes in WAGR syndrome.
Ethics oversight	IRB of the National Institute of Child Health and Human Development

Note that full information on the approval of the study protocol must also be provided in the manuscript.

Clinical data

Policy information about clinical studies

All manuscripts should comply with the ICMJEguidelines for publication of clinical research and a completedCONSORT checklist must be included with all submissions.

Clinical trial registration	NCT00758108
Study protocol	www.clinicaltrials.gov
Data collection	National Institutes of Health Clinical Center, Bethesda, MD, USA, time period: 2008-2014
Outcomes	This is a secondary sub-study analyzing data previously obtained for this WAGR phenotyping protocol. The decision to analyze corpus callosum volume was based on the mechanistic evidence for the potential function of C11orf46.

Flow Cytometry

Plots

Confirm that:

- [x] The axis labels state the marker and fluorochrome used (e.g. CD4-FITC).
- [x] The axis scales are clearly visible. Include numbers along axes only for bottom left plot of group (a 'group' is an analysis of identical markers).
- [x] All plots are contour plots with outliers or pseudocolor plots.
- [ ] A numerical value for number of cells or percentage (with statistics) is provided.

Methodology

Sample preparation	Neuronal cells isolated from mouse brain cortex after in utero electroporation (IUE).
Instrument	FACSAria IIμ, FACSAria III
Software	FACSDIVA
Cell population abundance	Abundance of GFP positive cells was 5-10%.
Gating strategy	Sort by cell size> 2 step removal of cell aggregates > GFP positive cell collection through PE-A channel.

☒ Tick this box to confirm that a figure exemplifying the gating strategy is provided in the Supplementary Information.

Magnetic resonance imaging

Experimental design

Design type	Retrospective observational cohort study.
Design specifications	Comparison of corpus callosum volume adjusted for age and sex at a single time point for control subjects, PAX6 haploinsufficient patients, and PAX6/C11orf46 haploinsufficient patients.
Behavioral performance measures	Not applicable.

Acquisition

Imaging type(s)	Structural
Field strength	3T
Sequence & imaging parameters	Brain MRI consisted of one cubic millimeter resolution, T1-weighted images collected on a 3.0 T Philips Achieva MRI scanner with an 8-channel phased array head coil. Corpus callosum volumes were calculated using FreeSurfer's (version 5.3) subcortical image processing pipeline and published methods (Fischl, B. et al. Whole brain segmentation: automated labeling of neuroanatomical structures in the human brain. Neuron 33, 341-55 (2002). Briefly, the pipeline uses prior probability of a given tissue class at a specific atlas location, the likelihood of the image intensity given the tissue class, and the probability of the local spatial configuration of labels given the tissue class. The measured corpus callosum volume extends 2.5 mm from the midline on both sides to mitigate against any residual misalignment after registration to the template. Analysis of MRI data was performed blinded to genetic diagnosis of each patient.
Area of acquisition	Whole brain

Diffusion MRI ☐ Used ☒ Not used

Preprocessing

Preprocessing software	FreeSurfer's (version 5.3) subcortical image processing pipeline
Normalization	MRI brain scans were normalized to a T1-weighted template using a 12-parameter affine transformation.
Normalization template	MRI brain scans were normalized to the MNI305 template.
Noise and artifact removal	No artifact or structured noise removal was applied to the MRI scans.
Volume censoring	Each subject had a single structural MRI scan so volume censoring does not apply.

Statistical modeling & inference

Model type and settings	Univariate fixed effect ANCOVA including age at time of MRI brain scan and sex as covariates.
Effect(s) tested	The effect of diagnostic group was assessed using ANCOVA.

Specify type of analysis: ☐ Whole brain ☒ ROI-based ☐ Both

Anatomical location(s)	Corpus callosum volumes were calculated using FreeSurfer's (version 5.3) subcortical image processing pipeline and published methods (Fischl, B. et al. Whole brain segmentation: automated labeling of neuroanatomical structures in the human brain. Neuron 33, 341-55 (2002). Briefly, the pipeline uses prior probability of a given tissue class at a specific atlas location, the likelihood of the image intensity given the tissue class, and the probability of the local spatial configuration of labels given the tissue class. The measured corpus callosum volume extends 2.5 mm from the midline on both sides to mitigate against any residual misalignment after registration to the template.

Statistic type for inference (See Eklund et al. 2016)	Standard inferential statistics were performed on ROI measures. No voxel-wise or cluster-wise measures were used.
Correction	Bonferroni correction was applied to multiple comparison between groups.

Models & analysis

n/a	Involved in the study
☒	☐ Functional and/or effective connectivity
☒	☐ Graph analysis
☐	☒ Multivariate modeling or predictive analysis

Multivariate modeling and predictive analysis	ANCOVA adjusting for age and sex compared corpus callosum volume by genotype
