# Supplementary material for: In vivo epigenetic editing of Sema6a promoter reverses transcallosal dysconnectivity caused by C11orf46/Arl14ep risk gene
Source: Nat Commun. 2019 Sep 11;10:4112. doi: 10.1038/s41467-019-12013-y (PMC6739341; doi:10.1038/s41467-019-12013-y)
Supplement: Supplementary file 2 — Peer Review [file 41467_2019_12013_MOESM2_ESM.pdf]

Reviewers' comments:

Reviewer #1 (Remarks to the Author):

In their manuscript, Peter Saito et al explore the role of C11orf46 (also known as ARL14EP), a small nuclear protein encoded in the chromosome 11p13 WAGR risk locus. The authors correlate C11orf46 haploinsufficiency in WAGR microdeletion cases with severe hypoplasia of the corpus callosum and show that in utero shRNA-mediated C11orf46 knockdown (KD) disrupts transcallosal projections of cortical pyramidal neurons. The authors also show that multiple genes encoding key regulators of axonal growth and differentiation, including Sema6A, are upregulated in C11orf46 KD neurons. Finally the authors show that RNA-guided epigenetic editing of neuronal Sema6a gene promoters via a dCas9 protein 40 conjugated SunTag scaffold with multimeric C11orf46 binding results in normalization of Sema6a gene expression and rescue of transcallosal dysconnectivity via repressive chromatin remodeling. Overall, a key advancement of this work is linking mutations predisposing to neurodevelopmental disorders with cortical axonal growth alterations and chromatin remodeling deficits, as well as highlighting the therapeutic potential for repairing brain wiring in neurodevelopmental disorders via gene-targeted designer activators and repressors.
Strengths of this manuscript include the demonstration of axonal growth deficits induced by C11orf46 KD and the fact that the authors take on multiple experimental paradigms to not only delineate the cortical axonal alterations resulting from C11orf46 KD, but also offer detailed biochemical signaling based insights as to how C11orf46 deficiency may lead to axonal deficits via downstream targets. Weaknesses include lack of corroboration of the findings in germline knock-out and knock-in mouse models that reflect more accurately the role of C11orf46-haploinsufficency in the phenotypes associated with the WAGR syndrome. Because of this the authors are not able to address the ultimate functional consequences (if any) of the observed alterations in transcallosal connectivity at the level of circuit connectivity and local synaptic plasticity. In utero electroporation of shRNAs, in particular, is powerful technique to study axonal growth but there are many examples where the germline KO does not show the effect observed in the acute shRNA-mediated KD. A good example is Dcx and Dclk1 genes, where the single KO mice show no neocortical defects, whereas acute KD of either shows clear defects (Baek et al 2014, PMID:24945770). The authors should at least discuss this important issue and clearly explain how they excluded potential contributions of off-target effects in their findings (the sequencing data may be helpful to this end). Another weakness is that a substantial part of the work relies on cell lines, including lymphoblastoid cell lines from WAGR cases, which may not represent adequately the transcriptional dysregulation in the brain. Despite these limitations the results offered by the authors (especially the biochemical data) are typically supported by multiple experimental approaches and appropriate controls and offer a number of plausible hypotheses regarding the mechanism of action of C11orf46, which can be tested in a more conclusive manner using appropriately engineered mouse models.

Reviewer #2 (Remarks to the Author):

The authors identified the role of a gene C11orf46, which is encoded from 11p13 WAGR risk locus, in the interhemispheric guidance of callosal projections. they authors show that in patients with haploinsufficiency for Pax6 and C11orf46 have reduced CC thickness. They show that C11orf46 is a chromatin modifier and is an essential constituent of KAP1-SETDB1 repressor complex. This complex assist in corpus callosum formation by suppressing the expression of Sema6A. Overall, this is an excellent manuscript describing the novel role of C11orf46 chromatin modification and corpus callosum formation. However, there are few issues which need to be addressed before publishing this manuscript.

1) The main limitation of this manuscript to make it more impactful is the lack of knockout animal models for the C11orf46 gene. The authors heavily relied on using in utero electroporation guided

shRNAs, which many times create off-target effects, especially when searching for downstream effector molecules. E.g. Using shRNAs, in fig 3B, 3C, and sgRNA guided localization of C11orf46 to Sema6A locus in figure 3A- 3D, the authors show that C11orf46 along with KAP1-SETDB1 complex represses the expression of Sema6A in upper layer neurons. However, in the lymphoblastoid cell lines generated from C11orf46 +/- patients showed a decrease in the expression of Sema6A as seen in Table S3. Can the authors please address this discrepancy in data. Would it be due to off-targets effects of shRNAs or would it be because of deletion of other genes, PAX6 and BDNF? Does this indicate that the regulation of Sema6A by C11orf46 is not specific?

2) Regarding regulatory role of C11orf46 onto Sema6A, following experiments are required before publication:
a) The authors should show by in-situ hybridizations the expression pattern of Sema6A in the cortex, in the electroporated brains?
b) Sema6A mostly interacts with Plxns to regulate the cortical projection guidance. The authors should explain either by experiments or by literature support how the regulation might be happening.
c) The authors should show that if over-expressing Sema6A by in utero electroporations alone would be sufficient to block midline crossing of callosal projections.

3) In figure 1I, 1J, 4F and 4G the authors showed the role of C11orf46 in CC formation by using in utero electroporations of shRNAs into the brain and then quantifying the GFP projections in the contralateral hemisphere. The number of projections in the contralateral hemisphere will also be affected by the amount of DNA electroporated at the first place. For the quantification analyses of GFP in the contralateral hemisphere, it must be normalized to the GFP detected in the electroporated side.

Minor concerns:

1. In figure 1A and S1, the authors showed that haploinsufficiency of Pax6 and C11orf46 leads to a reduced volume of the corpus callosum(CC). Pax6 mutants or deletions are already known to induce a reduction in the size of the CC. Although C11orf46, did increase the penetration and severity of CC phenotype, the authors do not see any patients with C11orf46 deletion or mutations alone with reduced CC volume. So, can the authors please elaborate the role of Pax6 in corpus callosum formation in discussion and describe how would C11orf46 augment the defects.

2. The chromatin modifiers are influential in regulating the fate of cortical neurons. SATB2 knockout in neurons deprives them from forming callosal projections. Can the authors please check that if in C11orf46 knockdown cortices, the number of cortico-cortical projecting neurons change by staining the sections for SATB2, CUX1 and BCL11B.

3. Figure 1C is labeled as RNA, whereas it is a western blot.

4. In Figure 1E, the authors should show in situ hybridizations for C11orf46 to show its expression across the cortex. This would further consolidate the validations of their home made antibody.

5. In Figure 1G, the cell density in CAMKII stainings look different from those seen in Olig2, GFAP, and IBA1 stainings. A better way to show this image would be to show the whole cortical wall and then zoom out on specific areas. Also. Please provide quantifications showing the co-positivity of these markers with C11orf46.

6. In Figure 1G, the punctate stainings for C11orf46 are not visible in GFAP and IBA1 panel. Please use better contrast color to pseudocolor C11orf46.

7. In Figure 2E, the color of the graph is different as that described in the legend.

8. In supplementary figure3, the font size for gene names is unreadable.

9. In supplementary table S3, please define the cell lines genotypes clearly. Are the numbers 1,2,3 and 4 represent the same cell lines as in supplementary figure 1?

Reviewer #3 (Remarks to the Author):

In this study, Peter et al. reveals a role for the small nuclear protein C11orf46 in transcallosal axon development.

The authors first show transcallosal dysconnectivity in patients with C11orf46 haploinsufficiency; they describe, in mice, the endogenous expression dynamics and specificity of C11orf46 during development, and show that silencing of C11orf46 in superficial layer neurons using shRNA reduces transcallosal axon projections. Using mass spectrometry to identify C11orf46 binding partners, they find interactions with SETDB1, a chromatin regulator. Differential gene expression analysis reveals de-repression of Sema6a. Finally, using RNA-guided epigenetic editing of Sema6a gene promoters, they show that targeting Sema6a promoter with C11orf46 rescues transcallosal connectivity.

Overall, the study is well conducted and has interesting implications in the understanding of a human genetic disorder. The link between axonal development genes and epigenetic regulation is of broad interest.

Major points:

1. Expression of C11orf46 in the developing cortex (Fig.1):

-To claim that C11orf46 has a peak of expression from E15 to P0, the authors should show and compare their data to the level of expression before E15 (1c,d). Alternatively, this claim can be removed.

-To claim that C11orf46 is mostly expressed in neurons, additional cell-type specific labelings are needed (PAX6/TBR2/NeuroD2) (1e,f).

-At these early stages, NeuN labeling is notoriously unreliable as the gene is barely expressed. Neuronal markers, like TBR1, SATB2, CUX1, CTIP2 should be used.

-Expression (or lack thereof) in radial glia should be better documented at embryonic stages (e.g. E14, E17).

2. Quantification of transcallosal connectivity (Fig.1 & Fig.4)

-The electroporation site corresponding to the axonal projection illustrations should be shown.

-The data should be normalized by the size of electroporation or by the density of electroporated cells, for each animal.

-The intensity of GFP at the midline (normalized by the electroporation site) should be quantified to determine the level at which midline projections are affected.

-Finally, analyzing fluorescence intensity per bin in the contralateral hemisphere, (as in Wang et al., 2007) would provide a more precise quantification of the phenotype (i.e. are all layers equally affected?).

3. Quantification of dendritic complexity (Fig.1)

-Higher resolution images of the dendrites should be shown (in 1k, the apical dendrite is barely visible and other processes are not visibles). 10x objective is definitely not high enough for such morphological analyses.

-Dendritic morphology should be reconstructed (using Imaris sofware for example).

-The number, length and branch points of dendritic trees (Sholl analyses) should be determined.

-Alternatively, claims on dendritic morphology could be removed, since this does not appear to be a central part of the phenotype.

4. Transcriptional analyses (Fig.3):

-What are the gene ontologies of the top genes (with or without KAP1 binding sites), besides those implicated in axonal development?

-The graph in Fig.3a is not clear: y-axis is annotated as "mRNA expr level (Dox+/Dox-)", which would mean that a value of 1 corresponds to no change between two conditions. If it is the case, then C11ord46 and Ndrg1 are down-regulated (<1) and most of the other genes do not show actual alterations, while in the text it is stated that "17 axonal growth and development genes were altered in response to C11ord46 shRNA". Please clarify.

-Related to the point above, in Fig.3c: How to explain that the effect claimed for Fig.3a on Gap43 and Cdlk1 expression is not reproduced in this experimental paradigm?

5. Experiments in Fig. 4 have to be more carefully and precisely described:

-Fig.4c: In the text, the authors claim that "3 of the 4 sgRNAs to SEMA6A […], resulting in significant decrease in SEMA6A expression when co-transfected with …". The actual results show only 2 out of 4 sgRNAs with significant effect on Sema6A expression.

-Why is the positive control effect of promoter editing on Dclk1 and Gap43 (5 to 10x increased expression) is not seen with 3 out of 4 sgRNA targeting of the Sema6a? Why do the authors investigate these two genes when Fig.3 shows they are not regulated?

6. Author contributions

No less then 10 authors are described as solely having “assisted” experiments. Their role should be better explained, since some of these procedures (e.g. in utero electroporation) do not typically require experimental assistance.

Minor points:

Text:

1. Fig.1f: show low mags before high mags.

2. p.6, l.127: correct typo: “data not shown”

3. Fig.1h & Fig.4f: show these results as a stack of images from distinct animals (one color per animal), to illustrate variability in layer invasion.

4. p.7, l.152: replace “on the neuronal connectome” by “corpus callosum development”.

5. p.7, l.173: correct typo: “(Fig. 2g,h)“.

6. p.8, l.196: indicate how many genes are up/down-regulated.

7. p.9, l.223: correct typo: “CRISPR-associated protein Cas9”.

8. p.10, l.237: the “pool of four” is shown only for Sema6A condition, the sentence needs therefore to be changed to fit with the results.

9. p.10, l.237-238: correct typos and writing: “Interestingly, introduction of 3 of the 4 sgRNAs to SEMA6A, which is expressed at much higher levels as compared to the other test genes (Fig. 4c), resulteding in significant decrease in SEMA6A expression when co-transfected with the dCas9-ST10x C11orf46 (wt) systems”.

10. p.10, l.264: correct typo and wording: “…, which both wasere de-repressed after both …”

11. p.10, l.249: correct typo: “Sema6a”.

12. Uniform Sema6A wording (SEMA6A, sema6a, Sema6A) should be used all over text and figures. The consensus is as follow: GENE, PROTEIN for human, and Gene, PROTEIN for mice.

13. p.11, l.259: repetition: “development is a pivotal developmental process”.

Figures:

Fig.1j,k: Add age of animals used for analyses in the figure (like in e-g).

Fig.3a: Clarify y-axis.

Fig.4c: Add "HEK293 cells" in the panel.

Fig.4d: Uniform gene/transcript/protein wording for title and y-axis. Right graph does not have the same y-axis format as others.

Supplementary Figures:

Sup Fig.1b,d: Give full gel images for western blots.

Sup Fig.1b: Check y-axis (RNA two times).

Sup. Fig.1e: Check size of "Rescued cDNA"

Sup. Fig.3b: Chose more appropriated color scale for the heatmap. Indicate the units of "expression level" (log? normalization?)

Responses to reviewers

Reviewer #1:

We would like to thank the reviewer for this comment: "Strengths of this manuscript include the demonstration of axonal growth deficits induced by C11orf46 KD and the fact that the authors take on multiple experimental paradigms to not only delineate the cortical axonal alterations resulting from C11orf46 KD, but also offer detailed biochemical signaling based insights as to how C11orf46 deficiency may lead to axonal deficits via downstream targets" and "the results offered by the authors (especially the biochemical data) are typically supported by multiple experimental approaches and appropriate controls and offer a number of plausible hypotheses regarding the mechanism of action of C11orf46".

Weaknesses include lack of corroboration of the findings in germline knock-out and knock-in mouse models that reflect more accurately the role of C11orf46-haploinsufficency in the phenotypes associated with the WAGR syndrome. Because of this the authors are not able to address the ultimate functional consequences (if any) of the observed alterations in transcallosal connectivity at the level of circuit connectivity and local synaptic plasticity. In utero electroporation of shRNAs, in particular, is powerful technique to study axonal growth but there are many examples where the germline KO does not show the effect observed in the acute shRNA-mediated KD. A good example is Dcx and Dclk1 genes, where the single KO mice show no neocortical defects, whereas acute KD of either shows clear defects (Baek et al 2014, PMID:24945770). The authors should at least discuss this important issue and clearly explain how they excluded potential contributions of off-target effects in their findings (the sequencing data may be helpful to this end).

We appreciate the reviewer's constructive criticism and agree that it is important to investigate the effect of *C11orf46*-haploinsufficiency in genetically engineered mouse models of *C11orf46* to elucidate C11orf46-mediated mechanisms underpinning WAGR syndrome-related phenotypes. Nonetheless, as the reviewer pointed out, we respectively note that *in utero* electroporation-knockdown approach is a powerful method to examine the cell-autonomous effect of C11orf46 in the specific time critical for axonal growth – a necessary step to discern molecular mechanisms underlying development of transcallosal projection. To ensure that observed results do not originate from their off-target effects, we confirmed dose-dependent effects on the axonal phenotypes induced by two shRNAs with independent target sequences as shown in **New Fig. 2**. Rescue experiments by co-expression of RNAi-resistant wild type C11orf46, containing silent mutations in the target sequence of their shRNA, also ensured that the observed phenotypes are specifically due to its knockdown effect. Nevertheless, C11orf46-mediated mechanisms for axonal development and resulting WAGR syndrome-related phenotypes should be investigated in mice with genetic deletion of *C11orf46,* which will be considered for future studies. Following the reviewer's suggestion, we have referred to the significance of this question for future studies with additional references in the revised Discussion section (Page 14).

Another weakness is that a substantial part of the work relies on cell lines, including lymphoblastoid cell lines from WAGR cases, which may not represent adequately the transcriptional dysregulation in the brain.

We agree that lymphoblastoid cells may not represent brain-specific epigenome. Nevertheless, lymphoblastoid cells remain to be a convenient and well-characterized tool to elucidate brain disease-related molecular phenotypes (Gurwitz et al., Dialogues Clin Neurosci 2016). Importantly, our RNA-seq data sets with matched controls revealed not only lymphoid genes, but also genes critical for neuronal function; representing the disturbances of neuronal-like epigenome in these lymphoid cells. We have added these statements with additional references and description of limitation of lymphoblastoid cell lines in the Discussion section (Page 14) in the revised manuscript.

Reviewer #2:

We would like to thank this reviewer for the comment: "Overall, this is an excellent manuscript describing the novel role of C11orf46 chromatin modification and corpus callosum formation".

1) The main limitation of this manuscript to make it more impactful is the lack of knockout animal models for the C11orf46 gene. The authors heavily relied on using in utero electroporation guided shRNAs, which many times create off-target effects, especially when searching for downstream effector molecules.

The reviewer has brought up an important point which Reviewer #1 also raised. As described above, while it is important to produce *C11orf46* knockout mice models to elucidate C11orf46-mediated mechanisms underpinning WAGR syndrome-related phenotypes, *in utero* electroporation is a powerful technique to examine, in particular, the cell-autonomous knockdown effect of *C11orf46* in the specific time critical for axonal growth. To ensure that observed results do not originate from their off-target effects, we confirmed dose-dependent effects on the axonal phenotypes induced by two shRNAs with independent target sequences as shown in **New Fig. 2**. Rescue experiments by co-expression of RNAi-resistant wild type C11orf46, containing silent mutations in the target sequence of their shRNA, also ensured that the observed phenotypes are specifically due to its knockdown effect. Nevertheless, C11orf46-mediated mechanisms for axonal development and resulting WAGR syndrome-related phenotypes should be investigated in mice with genetic deletion of *C11orf46,* which will be considered in future studies. Accordingly, we have referred to the significance of this matter in the revised Discussion section (Page 14).

E.g. Using shRNAs, in fig 3B, 3C, and sgRNA guided localization of C11orf46 to Sema6A locus in figure 3A- 3D, the authors show that C11orf46 along with KAP1-SETDB1 complex represses the expression of Sema6A in upper layer neurons. However, in the lymphoblastoid cell lines generated from C11orf46 +/- patients showed a decrease in the expression of Sema6A as seen in Table S3. Can the authors please address this discrepancy in data. Would it be due to off-targets effects of shRNAs or would it be because of deletion of other genes, PAX6 and BDNF? Does this indicate that the regulation of Sema6A by C11orf46 is not specific?

As described above, based on our results of dose dependent knockdown effect and rescue effect on observed phenotypes, molecular phenotypes are unlikely due to off-target effects of shRNAs to C11orf46. As noted by the reviewer, the discrepancy of Sema6A expression in lymphoblastoid cells and neuronal conditions likely arise from the confounding effect of other genes located in WAGR risk locus, including *PAX6* transcription factor. Epigenomic signatures of lymphoid cell line and developing brain may also contribute to this discrepancy. Accordingly, we have added these statements in the Discussion section (Page 14-15) in the revised manuscript.

a) The authors should show by in-situ hybridizations the expression pattern of Sema6A in the cortex, in the electroporated brains?

According to the reviewer's request, we have performed *in situ* hybridization experiments to examine expression of *Sema6a* and *C11orf46* in the cortex of electroporated brains. We found that Sema6a and C11orf46 are highly expressed in upper cortical layers II/III at P0, within the targeted time of brain development, via *in utero* electroporation. These results are shown in **new Supplementary Fig. 6a**.

b) Sema6A mostly interacts with Plxns to regulate the cortical projection guidance. The authors should explain either by experiments or by literature support how the regulation might be happening.

Our results indicated that proper control of Sema6a expression is critical for developing neuronal connectivity. Genetic deletion of *Sema6a* resulted in impaired neuronal connectivity (Rünker et al.,

PLoS One 2011; Håkansson K et al., Neurosci Lett 2017). Sema6a is a transmembrane protein and is known as a ligand for Plexin A2 (PLXNA2) and Plexin A4 (PLXNA4) which also play critical roles for regulating axon guidance. *Plxna2* and *Plxna4* knockout mice showed impaired axonal development similar to those observed in mouse models with genetic deletion of *Sema6a* (Mitsogiannis et al., Neural Dev. 2017). Thus, dysregulation of C11orf46-mediated control of Sema6a expression may impair axonal development regulated by Plxns either in cell autonomous or non-autonomous manner, which warrants further investigation in the future. This statement with additional reference is now in the Discussion section of the revised manuscript (Page 14).

c) The authors should show that if over-expressing Sema6A by in utero electroporations alone would be sufficient to block midline crossing of callosal projections.
According to the reviewer's request, we examined the effect of overexpression of Sema6A on axonal development by *in utero* electroporation. We found that overexpression of Sema6A elicited disturbance of midline crossing of callosal projections and axonal arborization during brain development. These results are shown in **new Supplementary Fig. 6c.**

3) In figure 1I, 1J, 4F and 4G the authors showed the role of C11orf46 in CC formation by using in utero electroporations of shRNAs into the brain and then quantifying the GFP projections in the contralateral hemisphere. The number of projections in the contralateral hemisphere will also be affected by the amount of DNA electroporated at the first place. For the quantification analyses of GFP in the contralateral hemisphere, it must be normalized to the GFP detected in the electroporated side.
According to the reviewer's suggestions, we re-analyzed axonal phenotypes induced by *in utero* electroporation-mediated C11orf46 targeting by normalizing with GFP signals detected in the electroporated side. The data was also re-evaluated by bin-based analysis across all cortical layers I-V according to the previously reported method (Wang et al., J Neurosci 2007). The results of quantification analysis are shown in **new Fig. 2b, 2c, 5f, 5g, and new Supplementary Fig. 6c**.

Minor concerns:
1. In figure 1A and S1, the authors showed that haploinsufficiency of Pax6 and C11orf46 leads to a reduced volume of the corpus callosum(CC). Pax6 mutants or deletions are already known to induce a reduction in the size of the CC. Although C11orf46, did increase the penetration and severity of CC phenotype, the authors do not see any patients with C11orf46 deletion or mutations alone with reduced CC volume. So, can the authors please elaborate the role of Pax6 in corpus callosum formation in discussion and describe how would C11orf46 augment the defects.
PAX6 is a transcription factor regulating neurogenesis and rostrocaudal patterning during development (Bishop et al., J Neurosci. 2002; Schuurmans et al., EMBO J. 2004). As the reviewer described, *PAX6* haploinsufficiency is associated with corpus callosum hypoplasia in both rodents and humans (Hiraoka et al., PLoS One. 2016; Free SL et al., Neuroimage 2003; Abouzeid H et al., Mol Vis. 2009; Boretius et al., Cereb Cortex 2009). Because human subjects in our study were recruited on the basis of having aniridia, all participants were, by definition, *PAX6* haploinsufficient, and, therefore, we were unable to assess the effect of *C11orf46*+/- independent of *PAX6*+/-. However, we were able to demonstrate that patients with combined loss of both genes had more severely reduced corpus callosum volumes compared to isolated *PAX6*+/-, confirming an additional role of C11orf46 in neurodevelopment. Whether the impact of mutations in the above molecules on axonal development is additive or synergistic remains to be determined. We have expanded the discussion section, which describes the importance of these topics in future studies (Page 14-15).

2. The chromatin modifiers are influential in regulating the fate of cortical neurons. SATB2 knockout in neurons deprives them from forming callosal projections. Can the authors please check that if in C11orf46 knockdown cortices, the number of cortico-cortical projecting neurons change by staining

the sections for SATB2, CUX1 and BCL11B.
According to the reviewer's suggestion, we examined the impact of *C11orf46* knockdown on the number of neurons labeled by SATB2 and CUX1. Given that our experimental design using *in utero* electroporation in this study specifically target layer II/III neurons, for these experiments, we did not use an antibody against BCL11B (CTIP2), which is expressed in deep-layer neurons. We observed no difference in percentage of SATB2 and CUX1 positive cells in GFP-labeled neurons in *C11orf46* knockdown brains, compared to those in controls. These results are shown in **new Supplementary Fig. 3d.**

3. Figure 1C is labeled as RNA, whereas it is a western blot.
We apologize for this error and have corrected the figure.

4. In Figure 1E, the authors should show in situ hybridizations for C11orf46 to show its expression across the cortex. This would further consolidate the validations of their home made antibody.
According to the reviewer's request, we performed *in situ* hybridization probing C11orf46 mRNA. We found that the signal distribution of C11orf46 mRNA is very similar to those of C11orf46 protein in the developing cerebral cortex. These results are shown in **new Fig. 1e.**

5. In Figure 1G, the cell density in CAMKII stainings look different from those seen in Olig2, GFAP, and IBA1 stainings. A better way to show this image would be to show the whole cortical wall and then zoom out on specific areas. Also. Please provide quantifications showing the co-positivity of these markers with C11orf46.
According to the reviewer's suggestion, we re-stained all of the cell-type markers with C11orf46 antibody. In these new experiments, we chose antibody against ALDH1L1 instead of GFAP to label protoplasmic astrocyte. The low magnification images are shown in **New Supplementary Fig. 2b** and the high magnification images are presented in **New Fig. 1g**. We also conducted quantitative analysis and the results suggest that C11orf46 is mainly expressed in CaMKII-positive neurons as shown in **new Supplementary Fig. 2b.**

6. In Figure 1G, the punctate stainings for C11orf46 are not visible in GFAP and IBA1 panel. Please use better contrast color to pseudocolor C11orf46.
According to the reviewer's request, we replaced the original images to new images of C11orf46 (red) with Iba1 and AlDH1L1 which was chosen instead of GFAP to label protoplasmic astrocyte as described above. The representative high magnification images are shown in **New Fig. 1g**. The low magnification images are also shown in **new Supplementary Fig. 2b.**

7. In Figure 2E, the color of the graph is different as that described in the legend.
This has been corrected as shown in **new Fig. 3e**.

8. In supplementary figure3, the font size for gene names is unreadable.
This has been corrected (Please see **new Supplementary Fig. 5**).

9. In supplementary table S3, please define the cell lines genotypes clearly. Are the numbers 1,2,3 and 4 represent the same cell lines as in supplementary figure 1?
In supplementary table 3, cell line genotypes are now clearly defined with corresponding numbers 1, 2, 3, and 4. These numbers correspond to the same cell lines as in the **new supplementary Fig. 1**.

Reviewer #3

We would like to thank this reviewer for the comment: "Overall, the study is well conducted and has interesting implications in the understanding of a human genetic disorder. The link between axonal development genes and epigenetic regulation is of broad interest".

Major points:

1. Expression of C11orf46 in the developing cortex (Fig.1):
-To claim that C11orf46 has a peak of expression from E15 to P0, the authors should show and compare their data to the level of expression before E15 (1c,d). Alternatively, this claim can be removed.
According to the reviewer's suggestion, this claim has been removed.

-To claim that C11orf46 is mostly expressed in neurons, additional cell-type specific labelings are needed (PAX6/TBR2/NeuroD2) (1e,f).
According the reviewer's request, we have performed co-staining experiment of PAX6, TBR2, and NeuroD2 together with C11orf46. Notably, C11orf46 is expressed in a nuclear punctate pattern in NeuroD2-positive cells, but not in PAX6 and TBR2-positive cells, although we observed cytoplasmic expression of C11orf46 in these markers-positive cells, suggesting that nuclear C11orf46 is primarily expressed in post-mitotic neurons. The data are shown in **New Fig. 1f** and **New Supplementary Fig. 2a.**

-At these early stages, NeuN labeling is notoriously unreliable as the gene is barely expressed. Neuronal markers, like TBR1, SATB2, CUX1, CTIP2 should be used.
According to the reviewer's request, we have performed co-staining experiment of TBR1, SATB2, CUX1, and CTIP2 together with C11orf46. In consistent with co-staining results other markers as described above, nuclear and cytoplasmic expression of C11orf46 was observed in these neuronal markers-positive cells. The data are shown in **New Fig. 1f and New Supplementary Fig. 2a.**

-Expression (or lack thereof) in radial glia should be better documented at embryonic stages (e.g. E14, E17).
According to the reviewer's request, we have performed co-staining experiment of Nestin, a radial glia marker, together with C11orf46. We found C11orf46 is expressed in the cytoplasm, but not in the nucleus in Nestin-positive cells as shown in **New Fig. 1f and New Supplementary Fig. 2a.**

2. Quantification of transcallosal connectivity (Fig.1 & Fig.4)
-The electroporation site corresponding to the axonal projection illustrations should be shown.
According to the reviewer's request, we have added a new image containing the electroporation site corresponding to the axonal projection in **New Fig. 2a**.

-The data should be normalized by the size of electroporation or by the density of electroporated cells, for each animal.
Reviewer #2 raised a similar request. As described above, we re-analyzed axonal phenotypes induced by *in utero* electroporation-mediated C11orf46 targeting by normalizing with GFP signals detected in the electroporated side. The data was also re-evaluated by bin-based analysis across all cortical layers I-V according to the previously reported method (Wang et al., J Neurosci 2007). The results of quantification analysis are shown in **new Fig. 2b, 2c, 5f, 5g, and new Supplementary Fig. 6c**.

-The intensity of GFP at the midline (normalized by the electroporation site) should be quantified to determine the level at which midline projections are affected.
According to the reviewer's request, GFP signal intensity at the midline has been quantified and normalized by the GFP signals in the elecrtoporated site in *C11orf46* knockdown experiments. We found reduction in thickness of corpus callosum elicited by knockdown of *C11orf46*, which is reversed by co-expression of RNAi-resistant wild type C11orf46, but not by C11orf46 (R236H) mutant. These results are shown in **New Supplementary Fig. 3c**.

-Finally, analyzing fluorescence intensity per bin in the contralateral hemisphere, (as in Wang et al., 2007) would provide a more precise quantification of the phenotype (i.e. are all layers equally affected?).
As described above, the data was re-evaluated by bin-based analysis across all cortical layers I-V according to the method in Wang et al., J Neurosci 2007. We found that *C11orf46* knockdown and overexpression of *Sema6a* equally affect axonal arborization in the all layers. The results of quantification analysis are shown in **new Fig. 2c, 5g, and new Supplementary Fig. 6c**.

3. Quantification of dendritic complexity (Fig.1)
-Higher resolution images of the dendrites should be shown (in 1k, the apical dendrite is barely visible and other processes are not visibles). 10x objective is definitely not high enough for such morphological analyses.
-Dendritic morphology should be reconstructed (using Imaris sofware for example).
-The number, length and branch points of dendritic trees (Sholl analyses) should be determined.
-Alternatively, claims on dendritic morphology could be removed, since this does not appear to be a central part of the phenotype.
According to the reviewer's suggestion, the data of dendrite morphologies has been removed in the revised manuscript.

4. Transcriptional analyses (Fig.3):
-What are the gene ontologies of the top genes (with or without KAP1 binding sites), besides those implicated in axonal development?
We appreciate this comment but feel that the fairly small number of genes altered (N=227) would not allow for a rigorous GO analysis. Instead, we used KAP-1 sites as additional filter and now revised, in order to address the Reviewer's point, the text as follows: "We identified 227 differentially expressed genes (182 down-regulated genes and 45 up-regulated genes) (FDR <0.05, see Methods; **Supplementary Fig. 5 and Supplementary Table 3**) that include 41 genes which have KAP1 binding sites (**Supplementary Table 4**). Notably, among these 41 genes, we found that 17 genes are reportedly involved in axonal development. (**Fig. 4a, Supplementary Table 4** genes marked in bold), while the remaining 24 genes, such as CAP2 encoding an adenylyl-cyclase associated protein, are linked to various other cellular functions (**Supplementary Table 4**)."

-The graph in Fig.3a is not clear: y-axis is annotated as "mRNA expr level (Dox+/Dox-)", which would mean that a value of 1 corresponds to no change between two conditions. If it is the case, then C11ord46 and Ndrg1 are down-regulated (<1) and most of the other genes do not show actual alterations, while in the text it is stated that "17 axonal growth and development genes were altered in response to C11ord46 shRNA". Please clarify.
We thank the Reviewer for this important comment and apologize for the unfounded statement in the Results section. In the revised version of our paper, we now write: "Nonetheless, we found that expression levels of the 17 axonal growth and development genes were not consistently affected in response to C11orf46 shRNA-mediated *C11orf46* knockdown in the neuroblastoma cell line. However, multiple putative C11orf46 target genes, including *Semaphorin 6a* (*Sema6a)*, *Doublecortin-*

like kinase 1 (*Dclk1*), *SLIT-ROBO Rho GTPase activating protein 3* (*Srgap3*), and others showed increased expression after *C11orf46* knockdown in the NSC-34 (**Fig. 4a**). Therefore, we wanted to confirm in neurons, *in vivo*, C11orf46's role as a transcriptional regulator during brain development, focusing on the three candidate genes with KAP binding sites proximal to transcription start site (TSS) (+/-750b), including two genes (*Sema6a, Dclk1*) with expression changes in response to *C11orf46* knockdown in the NSC-34."

-Related to the point above, in Fig.3c: How to explain that the effect claimed for Fig.3a on Gap43 and Cdlk1 expression is not reproduced in this experimental paradigm?
The results shown in the original Fig. 3a were obtained from NSC34 cell lines, a hybrid cell line, produced by fusion of motor neuron enriched, embryonic mouse spinal cord cells with neuroblastoma. Although *in vitro* neuronal cell lines such as NSC34 is a convenient model for initial screening to test whether loss of function of C11orf46 induces changes in transcriptome associated with neural development, we certainly acknowledge that cellular and epigenetic mechanisms in NSC34 cells may be different from those in developing pyramidal neurons in the mouse cerebral cortex.

5. Experiments in Fig. 4 have to be more carefully and precisely described:
-Fig.4c: In the text, the authors claim that "3 of the 4 sgRNAs to SEMA6A […], resulting in significant decrease in SEMA6A expression when co-transfected with …". The actual results show only 2 out of 4 sgRNAs with significant effect on Sema6A expression.
We thank the reviewer for their critical observations which have now been corrected (Page 11).

-Why is the positive control effect of promoter editing on Dclk1 and Gap43 (5 to 10x increased expression) is not seen with 3 out of 4 sgRNA targeting of the Sema6a?
As shown in **new Fig. 5c** (original Fig. 4c), *DCLK1* and *GAP43* are expressed at very low level in these cell lines, compared to *SEMA6A*. Thus, it is likely that further repression by C11orf46-mediated promoter editing may not be detectable. Nonetheless, the results obtained a VP64-mediated positive activation which indicates that these shRNAs are functional on the promoters of these genes.

Why do the authors investigate these two genes when Fig.3 shows they are not regulated?
We included the results of *DCLK1* and *GAP43* as controls for the experiment shown in **New Fig. 5d** (original Fig. 4d). sgRNAs to DCLK1 and GAP43 up-regulated expression of these genes when overexpressing dCas9-^{ST10xVP64}, whereas no significant expression changes were observed when overexpressing dCas9-ST$^{10x\ C11orf46\ (wt)}$, which may be due to no detectable expression level of *DCLK1* and *GAP43* as shown in **New Fig. 5c** (original Fig. 4c).

6. Author contributions

No less then 10 authors are described as solely having "assisted" experiments. Their role should be better explained, since some of these procedures (e.g. in utero electroporation) do not typically require experimental assistance.
According to the reviewer's suggestion, we revised the Author Contribution to clarify how each author contributed to this study. We also have added an additional author (Mohika Nagpal) who significantly contributed to the experiments for this revision. In addition, we acknowledged Dr. Elliot Androphy for providing NSC34 cells, Mr. Tomonori Matsushita for technical and intellectual assistance of experiments, and Dr. Scot C. Kuo for use of confocal microscope LSM 700 in the Acknowledgement section.

“C.J.P., A.S., S.A., and A.K. designed the study. C.J.P. performed biochemical and cellular experiments. A.S. performed biochemistry and *in vivo* experiments. T.F., C.R., A.D., A.G., L.D. performed biochemical experiments. A.S. performed *in utero* electroporation surgery. Y.H., Y.T., M.N., and E.A. performed the data analysis of axonal phenotypes and qPCR. G.P. maintained and prepared animals for *in utero* electroporation. J.G.P conducted gene search using Decipher database. A.D. and S.E. analyzed RNA-sequencing data. J.C.H. obtained human samples and generated lymphoblastoid cell lines. J.C.H., F.M.L., J.A.B. obtained and analyzed structural MRI data. C.J.P., A.S., S.A., and A.K. wrote the manuscript. All authors contributed to and have approved the final manuscript.”

Minor points:
Text:

1. Fig.1f: show low mags before high mags.
Low magnification images are placed in **New Supplementary Fig. 2a**.

2. p.6, l.127: correct typo: “data not shown”
This has been corrected (Page 6).

3. Fig.1h & Fig.4f: show these results as a stack of images from distinct animals (one color per animal), to illustrate variability in layer invasion.
According to the reviewer’s suggestion, we have made a stack images from distinct animals. However, as you can see the image below (presented as **Figure #1**), the image does not provide additional meaningful information. Furthermore, previous studies (e.g., Wang et al., J Neurosci 2007; Ageta-Ishihara et al., J Neurosci 2009) presented representative images of axonal projection from a single animal as we presented in this manuscript. Thus, we respectively note that the current style of image representation is useful for comparing results in other studies reporting axonal development mechanisms.

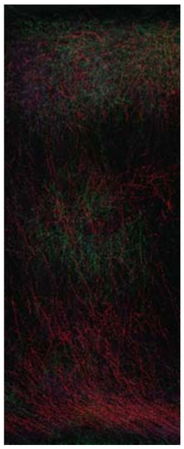

Figure #1 A stack of images from distinct five animals (Axons of distinct animals were depicted by different colors, including red, green, magenta, yellow, and cyan per animal).

4. p.7, l.152: replace “on the neuronal connectome” by “corpus callosum development”.
This has been corrected (Page 7).

5. p.7, l.173: correct typo: “(Fig. 2g,h)“.
This has been corrected (Page 8).

6. p.8, l.196: indicate how many genes are up/down-regulated.
These information has been included (Page 9).

7. p.9, l.223: correct typo: "CRISPR-associated protein Cas9".
This has been corrected (Page 10).

8. p.10, l.237: the "pool of four" is shown only for Sema6A condition, the sentence needs therefore to be changed to fit with the results.
This has been corrected (Page 11).

9. p.10, l.237-238: correct typos and writing: "Interestingly, introduction of 3 of the 4 sgRNAs to SEMA6A, which is expressed at much higher levels as compared to the other test genes (Fig. 4c), resulteding in significant decrease in SEMA6A expression when co-transfected with the dCas9-ST10x C11orf46 (wt) systems".
This has been corrected (Page 11).

10. p.10, l.264: correct typo and wording: "…, which both wasere de-repressed after both …"
This has been corrected (Page 11).

11. p.10, l.249: correct typo: "Sema6a".
This has been corrected (Page 11).

12. Uniform Sema6A wording (SEMA6A, sema6a, Sema6A) should be used all over text and figures. The consensus is as follow: GENE, PROTEIN for human, and Gene, PROTEIN for mice.
All mentions of Sema6A in the revised manuscript have been corrected according to the reviewer's advice.

13. p.11, l.259: repetition: "development is a pivotal developmental process".
We have deleted "developmental" from this sentence.

Figures:

Fig.1j,k: Add age of animals used for analyses in the figure (like in e-g).
We have added age of animals in the figures (**New Fig. 2d**)

Fig.3a: Clarify y-axis.
We have clarified it.

Fig.4c: Add "HEK293 cells" in the panel.
This has been corrected in the figure.

Fig.4d: Uniform gene/transcript/protein wording for title and y-axis. Right graph does not have the same y-axis format as others.
It has been corrected in the figure.

Supplementary Figures:

Sup Fig.1b,d: Give full gel images for western blots.
Previous images in Supplementary Fig. 1d have been replaced to the full gel images (Please see **New Supplementary Fig. 3a**). Regarding Supplementary 1b, we unfortunately missed the original full gel image. Thus, we deleted the data and kept the data of C11orf46 mRNA expression in the revised manuscript (**New Supplementary Fig. 1b**).

Sup Fig.1b: Check y-axis (RNA two times).
This has been corrected in the figure.

Sup. Fig.1e: Check size of "Rescued cDNA"
This has been corrected in the figure.

Sup. Fig.3b: Chose more appropriated color scale for the heatmap. Indicate the units of "expression level" (log? normalization?)
Color scale of **New Supplementary Figure 5b** (original Sup. Fig.3b) was changed to properly infer the transformation of data. The heatmap represents scaled rpkm values of the different conditions (also found in **Supplementary Table 3**), the transformation of the data consisted on scaling to the mean (or centering) using the scale R function (https://www.rdocumentation.org/packages/base/versions/3.6.0/topics/scale) to avoid underrepresented very high rpkm values to be extremely highlighted in the heatmap.

We appreciate your thoughtful handling of our paper and believe that this revised manuscript has addressed all the concerns raised by the reviewers. We hope the revised paper is now suitable for publication in Nature Communications.

REVIEWERS' COMMENTS:

Reviewer #3 (Remarks to the Author):

In this revised version, the authors have satisfactorily addressed all the points raised. I thank them for their work and consider that their article is now suitable for publication.

Dr. Jerome Staal
Associate Editor
Nature Communications

July 30, 2019

Responses to reviewers
Reviewer #3:

We would like to thank the reviewer for this comment: “the authors have satisfactorily addressed all the points raised. I thank them for their work and consider that their article is now suitable for publication”.

We hope the revised paper is now suitable for publication in Nature Communications.

Sincerely yours,

Atsushi Kamiya, M.D., Ph.D.
Schahram Akbarian, M.D., Ph.D.

Cc: Co-Authors
Enclosure

Atsushi Kamiya, M.D., Ph.D.
Associate Professor
Johns Hopkins Schizophrenia Center
Department of Psychiatry and Behavioral Sciences
Graduate Training Program in Cellular and Molecular Medicine
Johns Hopkins University School of Medicine

Schahram Akbarian, M.D., Ph.D.
Professor and Chief
Division of Psychiatric Eigenomics
Department of Psychiatry and Neuroscience
Friedman Brain INstitute
Icahn School of Medicine at Mount Sinai
